# Quantifying techno-functional properties of ingredients from multiple crops using machine learning

**DOI:** 10.1016/j.crfs.2023.100601

**Published:** 2023-09-22

**Authors:** Anouk Lie-Piang, Jos Hageman, Iris Vreenegoor, Kai van der Kolk, Suzan de Leeuw, Albert van der Padt, Remko Boom

**Affiliations:** a Food Process Engineering, Wageningen University, P.O. Box 17, 6700 AA, Wageningen, the Netherlands; b Biometris, Applied Statistics, Wageningen University, P.O. Box 16, 6700 AA, Wageningen, the Netherlands; c FrieslandCampina, Stationsplein 4, 3818 LE, Amersfoort, the Netherlands

**Keywords:** Food ingredients, Food formulation, Mild fractionation, Techno-functional properties, Machine learning

## Abstract

Food ingredients with a low degree of refining consist of multiple components. Therefore, it is essential to formulate food products based on techno-functional properties rather than composition. We assessed the potential of quantifying techno-functional properties of ingredient blends from multiple crops as opposed to single crops. The properties quantified were gelation, viscosity, emulsion stability, and foaming capacity of ingredients from yellow pea and lupine seeds. The relationships were quantified using spline regression, random forest, and neural networks. Suitable models were picked based on model accuracy and physical feasibility of model predictions. A single model to quantify the properties of both crops could be created for each techno-functional property, albeit with a trade-off of higher prediction errors as compared to models based on individual crops. A reflection on the number of observations in each dataset showed that they could be reduced for some properties.

## Highlights

• Techno-functional properties of mildly refined ingredients were quantified.
• Machine learning was used to quantify and predict techno-functional properties.
• Properties of ingredients from multiple crops were quantified with single models.
• The number of observations to quantify techno-functional properties can be reduced.

## Graphical abstract

**Figure:**
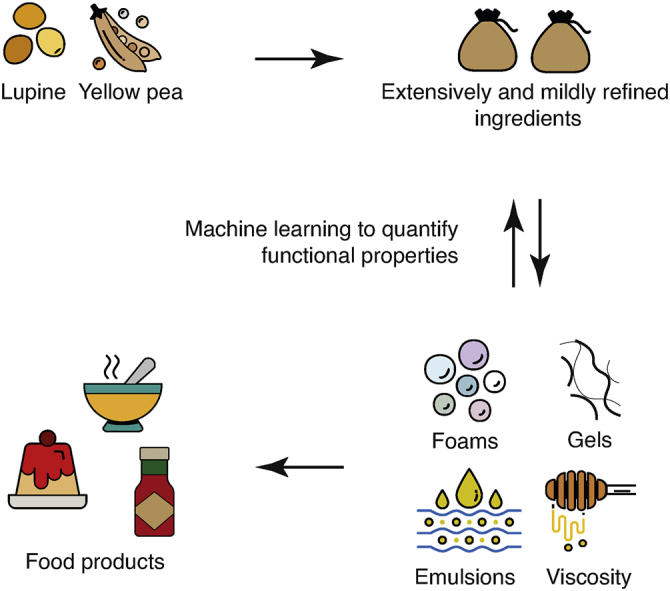

## 1 Introduction

Food ingredients with a lower extent of refining are more resource use efficient (Geerts et al., 2018; Lie-Piang et al., 2021), which in turn reduces the environmental footprint of food products. Less refined ingredients are alternative ingredients that may replace conventional isolates, such as protein or starch, that are commonly used to texturize food products. Air classification, electrostatic separation, and mild aqueous fractionation are all examples of methods that deliver functional ingredients, rather than pure conventional isolates (Cai et al., 2001; Schutyser and van der Goot, 2011; Zhu et al., 2021). As these fractions are not pure, it was proposed earlier to blend the ingredients to match the final composition of food products (Jonkman et al., 2020). Unconventional ingredients could not always be matched due to their complex compositions. This may not be necessary if a different composition could result in a product with the same functional properties. Therefore, food formulation practices would benefit from ingredient selection based on their functional properties, instead of composition.

Mathematical modelling or machine learning can be employed to quantify functional properties and subsequently formulate products, as was shown for example for chocolate cookies (Zhang et al., 2019). Ingredients can also be selected for formulations based on their structural contribution to food products, which was shown for the thickening capacity of yellow pea ingredients (Lie-Piang et al., 2022). In this study, formulations with different compositions and environmental impacts could be generated using the functionality-driven formulation approach using multiple linear regression with the composition of the ingredients as independent variables. To quantify other functional properties that are not linear, a framework was proposed to select a suitable machine learning algorithm (Lie-Piang et al., 2023). The models were subjected to the condition that the predictions were physically feasible, such as the absence of artefacts that could impede with the formulation. Since it could not be expected that one single algorithm always results in an acceptable quantification, a range of methods was explored.

While the previous work did demonstrate the principle of functionality-driven formulation, it was based on just one raw material. In practice, generally, more than one raw material is used to create a set of ingredients with suitable properties. Therefore, the proof of principle of the functionality-driven formulation should be extended to include multiple crops rather than one. Frequently used crops for ingredients are for example grains or legumes. Although most crops consist of the same basic building blocks protein, starch, fibre, oil, and sugars, they can yield different techno-functional properties. For example, fibres from different sources have different solubilities and water holding capacities (Elleuch et al., 2011). This complicates the optimization of ingredient blends made with different crops. Functionality-driven food product formulation would benefit from models that can quantitatively predict a functional property from multiple crops and hence have more general validity.

This study therefore assesses the potential to quantify and predict a techno-functional property of ingredients from multiple crops using a single model. The properties that will be assessed are gelation, viscosity, emulsion stability, and foaming capacity. This will be exemplified with conventional, high-purity ingredients and with mildly refined ingredients from yellow peas and from lupine seeds. First, the relation between the techno-functional properties and the composition of the ingredients will be discussed. Next, the relation between the composition of the ingredients and each techno-functional property will be quantified for each crop individually using machine learning. To assess whether models for multiple crops can be created for each property, new single models will be fitted to the data from both crops. These will be compared to the models based on the individual crops. We conclude with a critical discussion on the accuracy of the results as well as a reflection on the size of the dataset that is required to quantify each techno-functional property.

## 2 Materials and methods

### 2.1 Ingredients

Pre-dried yellow peas (*Pisum sativum* L.) were purchased from Alimex (The Netherlands). Pre-dried lupine seeds (*Lupinus angustifolius*) were obtained from InvejaFood (The Netherlands). Both yellow pea and lupine seed ingredients were produced using a pin mill (LV 15M, Condux-Werk, Germany), ZPS50 impact mill (Germany), and an ATP50 classifier (Hosokawa-Alpine, Germany) in a room with a controlled relative humidity of 30% and room temperature. A thermometer inside the mill indicated a temperature between 16 and 34 °C. The water used in all experiments was deionized in a Milli-Q purification system (Merck Millipore, Burlington, USA). Sunflower oil for the emulsions was purchased from a local supermarket. An overview of all ingredients is provided in Appendix A.1.

#### 2.1.1 Commercial isolates

Commercial isolates from yellow pea were a pea protein isolate (YPI) (Nutralys F85G) and a pea fibre isolate (YFI) (PEA FIBRE I 50 M), purchased from Roquette Frères S.A (St. Louis, USA) and a starch isolate from Emsland Stärke GmbH (Germany). A commercial protein isolate from lupine seeds (LPI) was obtained from Prolupin GmbH (Germany) and a fibre isolate (LFI) slurry as well. The fibre isolate slurry was freeze dried in a Pilot freeze dryer (Christ Epsilon 2-6D, Germany). The dried fibre isolate was subsequently milled in an impact mill with a mill speed set at 8000 rpm and an airflow of 75 m^3^/h and a classifier wheel speed of 2200 rpm. The feed rate was set at 7 rpm.

#### 2.1.2 Mildly refined ingredients

The hulls were first removed from the yellow peas with a Satake TM05 pearling machine (Satake Corporation, Japan). A yellow pea flour (YPF) was then produced by pre-milling the peas into grits using a pin mill at room temperature and then milling using an impact mill with an impact mill speed of 8000 rpm, airflow at 52 $m^{3}/h$, and classifier wheel speed at 4000 p.m., and a feed rate of 2 rpm (method adopted from Pelgrom et al. (2013)). The flour was subsequently air classified into a fine, protein-enriched fraction (YFF) and a coarse, starch enriched (YCF) fraction with a fixed airflow at 52 $m^{3}/h$, classifier wheel speed at 5000 rpm, and feed rate at 20 rpm.

Lupine seeds were used to prepare lupine flour (LF) from the non-dehulled seeds. First, the seeds were pre-milled with the pin mill, after which they were milled with a feed rate of 7 rpm in the impact mill at a milling speed of 8000 rpm and classifier wheel speed of 2200 rpm and an airflow of 75 $m^{3}/h$. A fine protein-rich fraction (LFF) and a coarse fibre-rich fraction (LCF) were subsequently produced with an air classifier at a feed rate of 20 rpm, classifier speed of 10,000 rpm, and an airflow of 80 $m^{3}/h$ (method adapted from Pelgrom et al. (2014)). For all in-house produced lupine seed ingredients, larger pieces of the hull were removed using a 0.16 mm grid sieve (Retsch, Germany) and later with an air jet sieve (Hosokawa-Alpine, Germany) with a grid size of 0.4 mm.

#### 2.1.3 Compositional analysis

The Kjeldahl method with a nitrogen conversion factor of 5.52 (Holt and Sosulski, 1979) for pea protein and 6.25 for lupine seed protein (Hove, 1974) were used to determine the protein content. The starch concentration in the samples was determined with a Total Starch Amyloglucosidase – α-Amylase Assay Kit, AOAC Method 996.11 (Megazyme International Ireland Ltd, Bray, Ireland). Next, AOAC method 985.29 was used to determine the total dietary fibre. The solids were determined by drying the ingredients in a vacuum oven at 70 °C. The rest fraction was determined as the difference between the dry matter and the protein, starch, and fibre content and contains among others oil, ash, salts, and sugars (Table 1).

**Table 1:** Composition of ingredients based on g/100 g dry matter and nitrogen solubility index (NSI%) with mean and standard deviation. Please note that there is a yellow pea flour only used for the viscosity measurements, due to different batches.

	Protein	+/−	Starch	+/−	Fibre	+/−	Rest	+/−	Solids	+/−	NSI%	+/−
YPI	74.1	1.8	0.4	0.0	2.2	1.2	23.3	1.6	93.7	0.5	16.4	5.8
YSI	0.5	0.0	91.4	2.9	0.8	0.8	7.3	0.0	88.4	0.5	0.0	0.0
YFI	7.8	0.5	33.8	0.5	53.6	4.6	4.8	1.1	95.2	0.5	34.7	8.9
YFF	44.4	1.1	6.9	0.2	18.9	2.9	29.8	2.1	93.5	0.5	48.0	0.8
YCF	10.5	0.5	66.6	1.9	4.6	1.5	18.3	1.6	90.9	0.5	55.3	7.5
YPF^a^	22.3	0.8	47.9	7.1	10.6	0.1	19.2	0.4	90.6	0.5	61.3	0.9
YPF	25.2	0.7	48.2	0.5	9.3	2.1	17.4	0.0	90.6	0.5	61.3	0.9
LPI	92.2	2.3	0.2	0.0	0.2	0.0	7.5	0.7	97.7	0.5	60.1	0.9
LFI	16.3	0.6	1.2	0.0	79.6	5.7	3.2	0.5	93.5	0.5	47.5	5.4
LF	36.8	1.0	0.2	0.0	39.3	4.1	23.7	2.2	92.8	0.5	21.7	1.5
LFF	55.1	1.4	0.2	0.0	14.6	2.5	30.1	2.4	94.4	0.5	19.4	0.5
LCF	30.4	0.9	0.2	0.0	49.0	4.6	20.4	2.0	92.2	0.5	33.3	3.7

a yellow pea flour batch used for the viscosity measurements.

The solubility of the protein in each ingredient was expressed with the nitrogen solubility index (NSI%) and was determined using an adopted protocol (Lie-Piang et al., 2023). Each ingredient was mixed thoroughly in a 1 wt% dispersion and rotated for 30 min, after which they were centrifuged (30 min, 10,000 g, at 20 °C). The pellet and supernatant were carefully separated. The pellet was further weighted in the tube and subsequently dried in an oven at 105 °C for 24 h. From the dried pellet the dry matter and protein content were determined. With the latter, the soluble protein was expressed in the NSI% as a percentage of soluble protein of the total protein present in the ingredients. All protein in yellow pea is considered denatured in the isolates (Tanger et al., 2020) and native in the mildly refined fractions. As the distinction in nativity between isolates and mildly refined ingredients in lupine seed ingredients was not that clear, this was not considered in the study (data not shown).

### 2.2 Selection of ingredient blends

The models in this study should be able to predict the techno-functional properties of all possible ingredient blends. The ingredient blends were chosen in such a way that the compositional space in which ingredients can be formulated was covered as best as possible (high and low concentration of starch, fibre, protein and the rest of the components) as described in another study (Lie-Piang et al., 2022). All yellow pea (YPI, YSI, YFI, YFF, YCF, and YPF) and lupine seed ingredients (LPI, LFI, LFF, LCF, LF) were therefore blended in the following combinations:

• yellow pea mixtures (isolates and mildly refined ingredients individually and mixed)
• lupine seed mixtures (isolates and mildly refined ingredients individually and mixed)
• yellow pea and lupine seed mixtures (isolates and mildly refined ingredients individually and mixed)

**Fig. 1:**
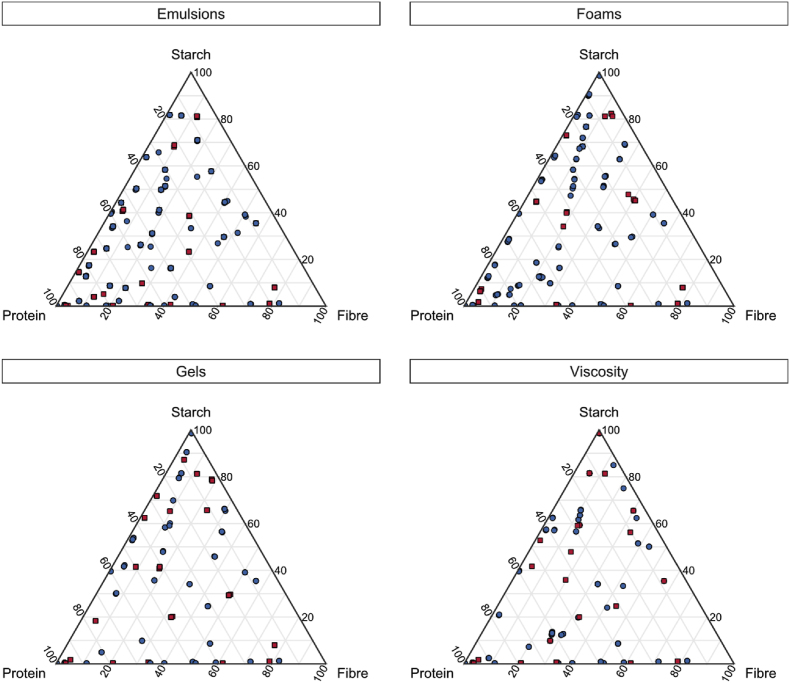
Ternary plots of the ingredient blends selected in this study, presenting their composition (ratio protein, starch, and fibre content) for measuring the four techno-functional properties with train (●) and test (■) observations. All determined in a concentration range of 1–25 wt%.

Fig. 1 depicts the ratio between the protein, starch, and fibre content for the three categories described above (with rest fraction in Appendix A.2). The rest fraction represents all dry components besides protein, starch, and fibre. All ingredient blends depicted in these Figures are measured between 1 and 25 wt%, which is discussed in section 2.4.1.2 in more detail.

### 2.3 Sample preparation and techno-functional properties

The emulsion separation velocity, foaming capacity, gel stiffness, and final viscosity were measured for all selected ingredient blends. Dry powders for all samples were always premixed and dispersed in deionized water at room temperature. All ingredient blends were measured once, with the exception of some duplicates or triplicates for protocol development.

#### 2.3.1 Emulsion separation velocity

The emulsion separation velocity (μm/s) was measured for 10 wt% (sunflower) oil emulsions with a total weight of 150 g. The aqueous phase was a dispersion of the selected ingredient blends, which were hydrated for 30 min under gentle agitation while covered with parafilm. The emulsion preparation was adapted from another study (Hinderink et al., 2021). The coarse emulsion was made using a rotor-stator homogenizer (Ultraturrax IKA T18 basic, Germany) at 11,000 rpm for 1 min. This pre-emulsion was further homogenized in a colloid mill (IKA Magic Lab, Germany) with a gap width of 0.16 for 2 min at 15,000 rpm. The separation (or creaming) velocity was measured with an adapted protocol with a LUMiFuge (LUM LUMGmbH, Germany) at constant gravitational acceleration of 2,227 rpm at room temperature with a light factor of 1.0 (Zhou et al., 2020). Measurements were taken every 15 s with 240 measuring points in total, which is equivalent to a storage time of approximately 30 days at 1g. The separation velocity (μm/s) was calculated using the LUMiFuge Front Tracking module at a transmission of 25% with equation (1) in which ΔL is the change of the position of the layer measured at 25% transmission for a time period Δt, only in the linear part.

(1) $$separation\;velocity=\frac{\left|\Delta L\right|}{\Delta t}$$

Since the LUMiFuge system measures the change of a creaming layer over time, we cannot exclude any effect of sedimentation on the position of the layer at 25%. Sedimentation can be observed by a movement downwards instead of upwards and measurements that clearly showed this behaviour were excluded. Dispersions that were too thick to measure were considered ‘stable’ in this method, i.e., having a separation velocity of 0 μm/s. Every emulsion was inspected using light microscopy (Axioscope, Zeiss, Germany) and captured with magnifications of 20x and 40x using a camera (AxioCam Mrc5, Zeiss, Germany) (data not shown).

#### 2.3.2 Foaming capacity

The foaming capacity, or overrun (%), was determined using an adapted protocol (Yang et al., 2022). The dry powders were dispersed using gentle agitation with a magnetic stirrer for 30 min. The samples were always covered with parafilm. 10 mL of the dispersion was subsequently foamed in a plastic tube (diameter 33.5 mm) using an overhead stirrer for 2 min at 2000 rpm with a froth (Aerolatte, UK). The foaming tubes were fixated at an angle of 45° to facilitate the incorporation of air. The fixation of the tube increases the reproducibility of the measurements. The foam height was directly captured by a camera and was recalculated to the volume. The foam overrun was determined with equation (2).

(2) $$Foam\;overrun\;\left(\%\right)=\frac{Foam\;volume\;\left(mL\right)}{Liquid\;volume\;\left(mL\right)}*100$$

#### 2.3.3 Gel stiffness

The gel stiffness in terms of Young's modulus was measured according to an existing protocol (Lie-Piang et al., 2023). The ingredients blends were initially dispersed for 5–10 min and then hydrated for 1 h while stirring gently and covered with parafilm. The dispersions were subsequently transferred to Teflon tubes (20 mm diameter and 100 mm length) and heated in a rotating water bath at 90 °C for 30 min at a speed of 30 rpm. After heating the tubes were removed from the water bath and left to rotate at room temperature for 1 h to cool down and prevent sedimentation. The tubes were further cooled overnight in the fridge at 4 °C and then again removed from the fridge 1 h before measurement. The gels were released from the tubes and cut into 3x20 mm long cylinders. The Henky's stress and strain (Urbonaite et al., 2014) were measured and calculated for all three cylinders and averaged.

#### 2.3.4 Final viscosity

The heated final viscosity was measured according to the method described in another study (Lie-Piang et al., 2022), in which the dispersions were first hydrated for 5–10 min, depending on the consistency. The viscosity was determined with an Anton Paar Rheometer MCR301 equipped with a starch cell C-ETD160/ST (Anton Paar, Austria). The dispersions were stirred at 600 rpm for 10 s at 50 °C to reassure a thorough dispersion prior to starting the measurement. The viscosity measurements were performed at a constant rotational speed of 160 rpm, and a temperature–time profile was used to heat all samples to 95 °C and subsequently cool them to 50 °C in a total of 25 min. Measurement of the unheated final viscosity was performed according to the same protocol without the heating ramp.

### 2.4 Data analysis

#### 2.4.1 Quantification of techno-functional properties

##### 2.4.1.1 Model training, testing, and selection

The relations between the selected ingredient blends and the techno-functional properties were quantified using a range of machine learning methods according to the framework presented in a previous study (Lie-Piang et al., 2023). For a detailed description of each method we refer to this article. The selection of these methods was based on creating a range of models that can describe linear and non-linear relations, to find the model that has the best physically plausible prediction with the highest prediction accuracy. 1) a stepwise AIC multiple linear regression, 2) the same stepwise AIC linear regression but with a log transformed output, 3) polynomial linear regression (power 2–4), 4) a regularized form of the polynomial linear regression to reduce parameters, 5) a spline regression, 6) a random forest, and 7) a neural network. For the neural network, the dataset was centred and scaled and fitted with a Sigmoid function. All models were fitted using adopted stepwise linear regression or the Caret package version 6.0.92 (Kuhn, 2022) in RStudio V4.1.0 with the code published on Git@WUR (Lie-Piang, 2022).

All models were trained and tested using independent datasets, which is explained in more detail below. The (regularized) polynomial linear regression, spline regression, random forest, and neural network models contain hyperparameters, which set the architecture of the models. These are respectively the degree polynomial and the alpha and lambda regularization parameters, the number of splines and the degree polynomial, the number of predictors, and the number of hidden nodes. These hyperparameters were chosen (tuned) based on the average error obtained from 5 times repeated 10-fold cross validation from only the training set. Next, all models were tested again on the separate test dataset.

The foam dataset was initially filtered for dispersions that had a solid structure since the foam overrun could not be measured precisely. These solid and liquid foams were distinguished using a simple decision tree (Therneau and Atkinson, 2019) with the unheated viscosity as the input variable. The accuracy and resulting confusion matrices were provided to compare the performance of the classification, with the addition of the kappa value. The kappa value provides a correction for the random chance of values ending up as either liquid or solid foams (McHugh, 2012). Only the liquid foams were extracted from the dataset and subsequently used in further modelling steps.

For each functional property, a final model was selected based on three conditions: the selected model 1) does not return unfeasible negative values 2) shows physically feasible behaviour of each component without any artefacts and 3) has acceptable model metrics. The metrics evaluated are Root Mean Square Error (RMSE), R^2^ (Pearson correlation coefficient), and Mean Absolute Error (MAE). Model metrics based on transformed data (log metrics for the log scale linear regression model and centering and scaling for the neural network) are based on the back-transformed data to ensure a fair comparison between the different models (Kvålseth, 1985). The physical feasibility of each component in each model was assessed by applying the model to a theoretical dataset in which each component varies between 1 and 15 wt%, while the others remain constant.

##### 2.4.1.2 Datasets and variables used for modelling

In section 2.2 we discussed the selected ingredients blends that are required to cover the full formulation matrix. To answer the main question of this study, which is to assess whether a single model can quantify each techno-functional property of multiple crops, different datasets were modelled:

1. yellow pea data only: ingredient blends made from yellow pea commercial isolates and mildly refined ingredients
2. lupine seed data only: ingredient blends made from lupine commercial isolates and mildly refined ingredients
3. yellow pea and lupine seed data combined: datasets number 1 and 2 with the addition of ingredient blends from yellow pea and lupine seed ingredients mixed together.

For each techno-functional property, 218–508 observations were collected (Table 2). Sizes may vary as the datasets were developed individually and some sets were used for protocol development.

**Table 2:** Sizes of total datasets (n) for each functional property and the percentage of the dataset that was used as a test set. The measured concentrations at which the ingredient blends are trained and tested, as well as only tested are indicated.

	All data	Yellow pea	Lupine seed	Mix	Concentrations (wt%)	
Functional property	Total dataset n (%test)	Total dataset n (%test)	Total dataset n (%test)	Total dataset n (%test)	Training + test	Extra test
Emulsions	508 (38)	364 (35)	88 (44)	56 (48)	1, 1.5, 2, 2.5, 4, 9, 15, 21	6, 7, 12, 18
Foams	423 (29)	267 (22)	99 (37)	57 (47)	1, 2, 4, 5, 9, 15, 21	7, 12, 18
Gelation	242 (47)	163 (45)	52 (48)	27 (44)	13, 18, 25	15, 21
Heated viscosity	225 (24)	110 (14)	85 (34)	30 (37)	2-22^a^; 2, 6, 10, 14, 18, 22^b^	12^b^, 16^b^
Unheated viscosity	218 (25)	101 (14)	78 (36)	39 (33)	2-22^a^; 2, 6, 10, 14, 18, 22^b^	12^b^, 16^b^

a Measured for yellow pea ingredients blends.

b Measured for lupine seed ingredient blends.

Observations were divided into a training and a test set, in such a way that both sets had an equal representation with respect to the composition of protein, starch, fibre (Fig. 1). This could not be done randomly, since the spread of training and test data had to be even throughout the composition space. In addition, the selected ingredient blends are measured at specific concentrations between 1 and 25 wt%. To check for interpolation, some concentrations within the range are only tested, which is also indicated in Table 2. The viscosity dataset of yellow pea ingredients was not obtained in the same systematic way as the other functional properties. Therefore, it does not include specific training and testing concentrations and is rather measured throughout the concentration range.

The main macro components (MC) (protein, starch, fibre, and the rest of the components) represented in concentration (wt%) were always input variables. The output variables were always the techno-functional properties, being the final viscosity (mPa.s at 160 rpm), the gel stiffness (kPa), foaming capacity (overrun %), and the emulsion separation velocity (μm/s). Potential effects of the processing history on protein functionality were considered by splitting the input variable ‘protein’ according to its solubility or nativity where possible (e.g., denatured and native protein). This results in three sets of input variables: 1) the MCs, 2) MCs with protein split into soluble and insoluble protein, and 3) MCs with protein split into native and denatured protein. For the models based on all data, the input variables were also split according to crop for some properties (e.g., protein from yellow pea and protein from lupine seed).

Significant differences in model metric performance between the different sets of independent variables were assessed for models that used a cross validation (regularized polynomial regression, neural network, random forest, and spline regression). The models were run five times and significant differences were assessed using a Two-Way ANOVA and Tukey's post-hoc test (p ≤ 0.05) to aid in the selection using RStudio V4.1.0. When a large variation between the repetitions was detected, the model with the smallest test errors and acceptable behaviour was used for variable set selection and further analyses.

#### 2.4.2 Dataset size reduction

To examine the importance the size of the dataset, it is of interest to estimate the minimum sample size required to model the selected techno-functional properties and reach the optimal prediction accuracy. The relevance of the size of the datasets is assessed by running models on a subset of observations from the complete dataset. This is only performed on the final model selected based on all data. The number (*n*) of selected observations ranges from *n* = 10 (the minimum needed to create a model) to the size of the complete dataset. The selection of observations is done using the Kennard-Stone algorithm, which selects observations with a uniform distribution over the whole compositional space, starting with the indicated *n* number of observations spread out as possible based on the Euclidian distance (Kennard and Stone, 1969).

For all datasets (from *n* = 10 to the maximum possible number of observations), the prediction accuracy is determined by a (nested) leave-one-out-cross validation (LOOCV) (Hageman et al., 2017). This entails that for each dataset with length *n*, in an outer loop observation *i* is taken out of the dataset and the models are trained on *n-i* observations in an inner loop, using the regular model training and cross validation as described earlier, which is called the inner cross validation. The model cross validation was reduced to two times repeated 5-fold cross validation to reduce computing time. After the models in the inner loop were trained, the left-out observation *i* in the outer loop was predicted by the model. This is repeated for all *n* observations in the dataset until all have been left out and predicted. The prediction error ($Q^{2}$) is determined by equation (3) with $y_{i}$ being the observed value, $\hat{y}$ the predicted value from the outer loop, and $\bar{y}$ the mean of all observed values.

(3) $$Q^{2}=1-\frac{\sum {\left(y_{i}-\hat{y}\right)}^{2}}{\sum {\left(y_{i}-\bar{y}\right)}^{2}}$$

## 3 Results

### 3.1 Experimental data techno-functional properties

**Fig. 2:**
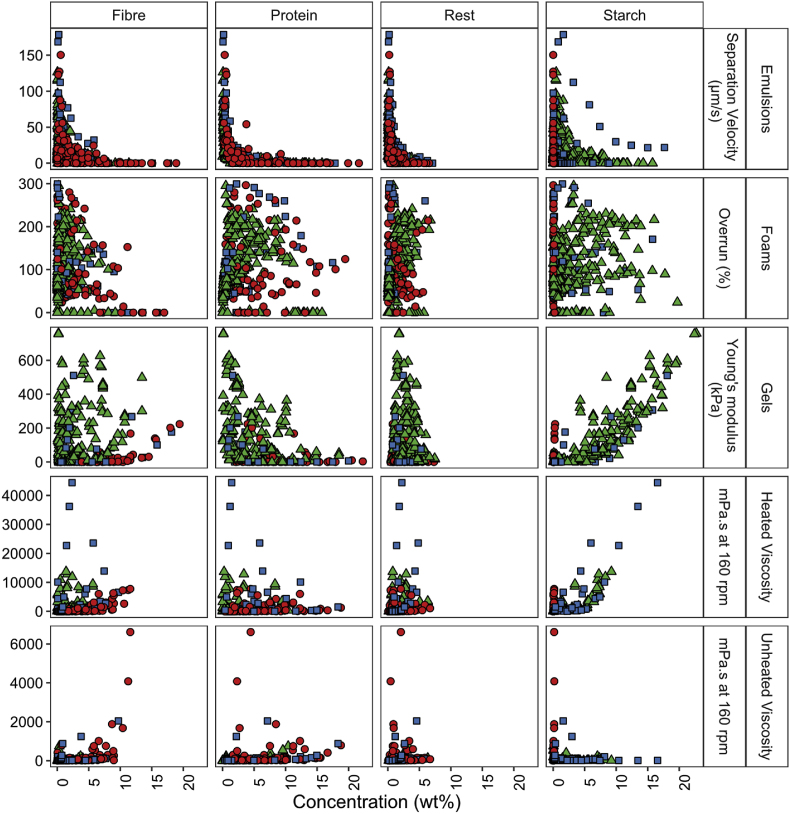
Scatterplots showing the relation between the techno-functional properties and the selected ingredient blends. The emulsion separation velocity (μm/s), foam overrun (%), gel Young's modulus (kPa), heated and unheated viscosity (mPa.s) are presented as a function of the concentration (wt%) of each component (protein, starch, fibre, and rest). The colour and shape indicate the crop: yellow pea▲, lupine seed ●, and mixtures of both ■. (For interpretation of the references to colour in this figure legend, the reader is referred to the Web version of this article.)

Fig. 2 shows the data of all ingredients blends obtained for the techno-functional properties selected in this study. The emulsion stability, foaming capacity, gel stiffness, and viscosity (heated and unheated) are presented as a function of the composition of the ingredients blends.

The gel stiffness ranges from around 0 to 800 kPa and is mainly dominated by the presence of starch, which was also found in another study (Pelgrom et al., 2015). The gel stiffness of gels made with ingredients from lupine seeds is lower than with yellow pea and mixtures of both crops due to the low starch concentration in lupine seeds. The heated viscosity ranges from 1 to approximately 45,000 mPa s and is also mainly dependent on starch gelatinisation (Singh, 2020, para. 5.5.1). The unheated viscosity of all pastes is more dependent on the capacity of the insoluble material to absorb water. This was found relatively high for fibre and protein in yellow pea in another study for yellow pea, as opposed to starch (Pelgrom et al., 2015). The dominating effect of fibre in lupine seeds was also hypothesized to be largely responsible for the viscosity in suspensions (Pelgrom et al., 2014). The unheated viscosity is significantly lower than in the heated variant, ranging from 1 to 6,000 mPa s.

The stability of emulsions is influenced by a combination of interfacial stabilisation as well as viscosity (McClements, 2005). The emulsion separation velocity of the measured emulsions ranges from 0 to approximately 160 μm/s, with 0 μm/s being completely stable in the theoretical period of one month at 1g. The separation velocity of the measured emulsions is highly dependent on the total concentration of the ingredients. Subtler differences in the ability of the components to stability interfaces are difficult to observe in these plots and are also not in the scope of this study.

The foaming capacity of the measured foams can be divided into two regimes: 0–2.5 wt% protein and >2.5 wt% protein. In the first regime, the foaming capacity increases with protein concentration. This can be attributed to interfacial stabilisation by proteins and increasing viscosity due to the generally higher concentration of the ingredients (Townsend and Nakai, 1983). In the second regime, the foaming capacity reaches a plateau or decreases again with increasing concentration of ingredients in the foams. We hypothesized that the latter occurs due to the increase in viscosity, which complicates the incorporation of air in the foams. In some cases, the viscosity becomes so high that the dispersion had no liquid layer (free water) in the measuring tube anymore. As a result, for these solid-like foams there is no or little incorporation of air and therefore the height of the foam cannot be determined anymore. Hence, the foaming capacity is registered as 0%. However, the foam overrun can also be 0% due to a lack of components that can form a foam in a more liquid foam. To prevent any confusion in the interpretation of a 0% foam overrun in a liquid or solid-like foam, the latter is filtered out of the dataset using a decision tree using their unheated viscosity, which will be explained further in a later section.

### 3.2 Quantification of the techno-functional properties

**Fig. 3:**
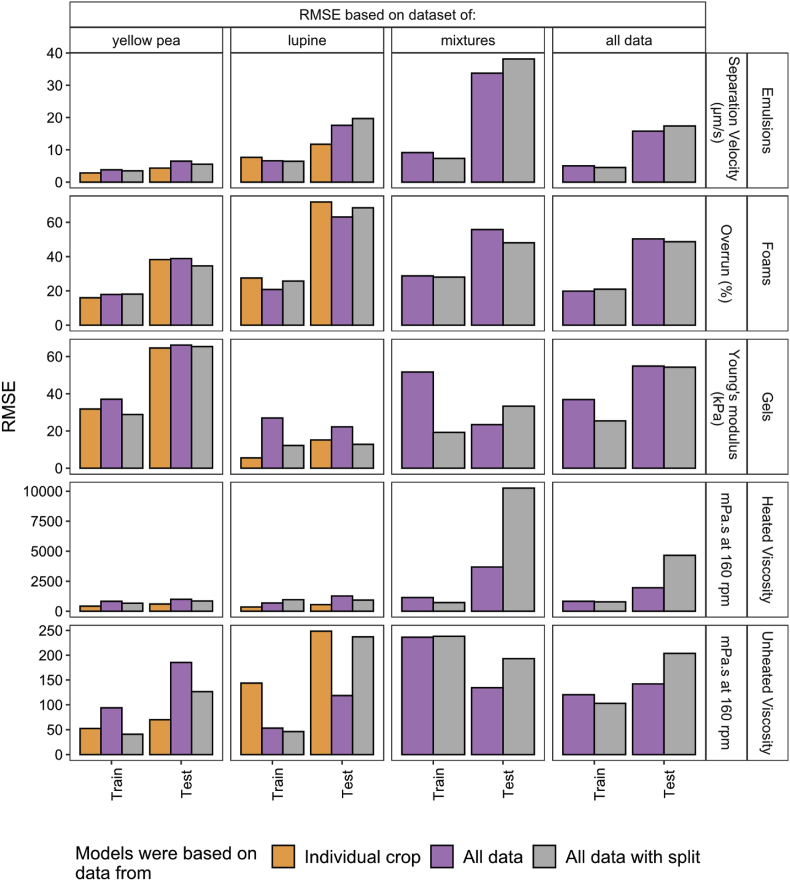
RMSE of the models based to predict the emulsion separation velocity (μm/s), foam overrun (%), gel Young's modulus (kPa), heated and unheated viscosity (mPa.s). Models were trained on the data from individual crops (yellow pea and lupine seed) and on all data (yellow pea, lupine seed, and mixtures of those) and on all data with a split in protein and fibre according to origin. The RMSE is calculated for the samples of yellow pea, lupine seed, mixtures, and all data together. (For interpretation of the references to colour in this figure legend, the reader is referred to the Web version of this article.)

**Fig. 4:**
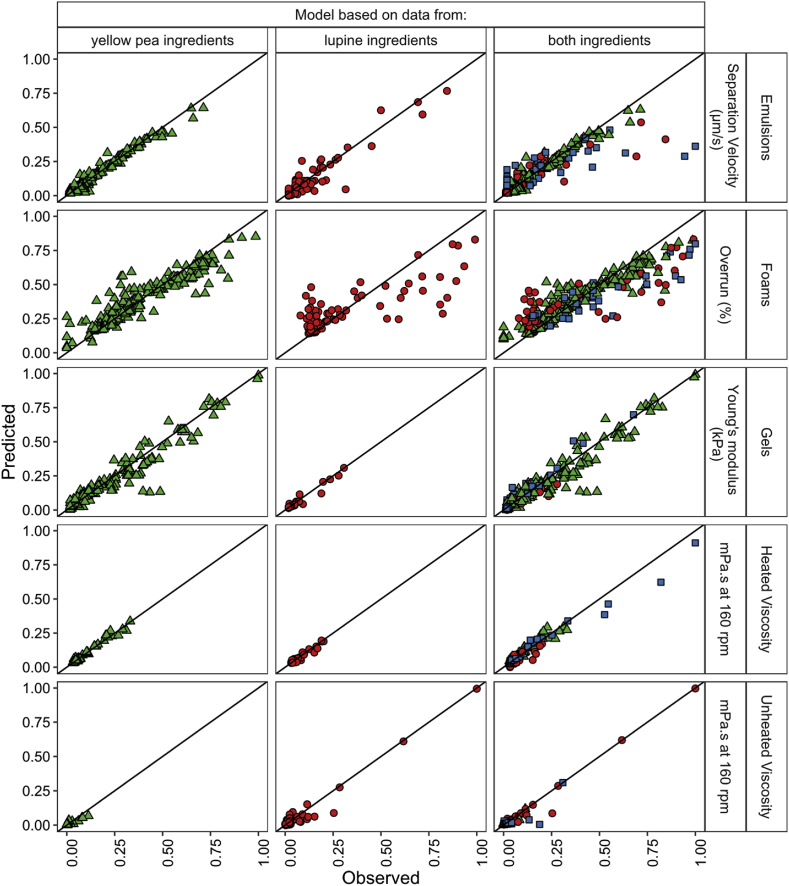
Normalized parity plots for three models based on datasets from yellow pea, lupine seed, or all ingredients. Visualized for the emulsion separation velocity (μm/s), foam overrun (%), gel Young's modulus (kPa), heated and unheated viscosity (mPa.s) for both training and test observations. The colour and shape indicates the origin of the sample: yellow pea▲, lupine seed ●, and mixtures of both ■. (For interpretation of the references to colour in this figure legend, the reader is referred to the Web version of this article.)

Due to the large number of results that are generated during the selection of the appropriate model to quantify the relationships, the tables and figures of all models are supplied in the Supplementary Information with a short explanation of why certain models were selected. An example is provided in Appendix A.3. Table 3 summarizes the models that are selected to quantify the techno-functional properties. Fig. 3 depicts the RMSE of the models trained on the three datasets based on 1) yellow pea and lupine seed ingredients separately, 2) both ingredients as well as mixtures of those, and 3) both ingredients and mixtures but with the input variables split according to the origin of the crop. The raw data of the RMSE and also the R^2^ and MAE of these models are provided in Appendix A.4 with the tuned hyperparameters. Fig. 4 shows the parity for all selected models.

**Table 3:** Summary of models selected to quantify the relation between the ingredients and techno-functional properties with the main macro components (MC) or with the main macro components with the protein separated according to solubility (SL) as input variables. The split column indicates whether models containing data from all crops included a split in independent variables according to crop.

Functional property	Crop	Best model	Variable set	Split crop
Emulsion stability	Yellow pea	Random forest	SL	–
	Lupine seed	Neural network	MC	–
	All	Random forest	MC	Yes
Foaming capacity	Yellow pea	Random forest	MC	–
	Lupine seed	Random Forest	MC	–
	All	Random Forest	MC	Yes
Gelation	Yellow pea	Neural network	MC	–
	Lupine seed	Neural network	MC	–
	All	Neural network	MC	No
Heated viscosity	Yellow pea	Spline regression	MC	–
	Lupine seed	Neural network	SL	–
	All	Spline regression	MC	No
Unheated viscosity	Yellow pea	Regularized polynomial linear regression	MC	–
	Lupine seed	Neural network	MC	–
	All	Neural network	MC	Yes

#### 3.2.1 Pre-processing foaming dataset

The model for foaming capacity was trained using only the liquid foams. Liquid and semi-solid foams were classified using a decision tree with the unheated viscosity (quantified in this study) as the predictive variable for the separate crop and combined datasets (Table 4). The foams of the individual crops were classified as liquid or semi-solid foams with high test accuracy (>93%) and with a moderate to a strong level of agreement (κ of 0.63 and 0.84 for yellow pea and lupine seed respectively) with the test set (McHugh, 2012). The κ value indicates the probability of a sample ending up in one category by chance. The combined dataset, which includes yellow pea, lupine seed and mixtures of those, has a similar test accuracy (94%) and also a strong level of agreement (κ = 0.82). The difference between the datasets can be attributed to the classification threshold, which is dependent on the model of the unheated viscosity and can therefore vary among crops.

**Table 4:** Performance of classification of liquid and semi-solid foams.

Crop	κ - Train	Accuracy Train (%)	κ - Test	Accuracy Test (%)	Classification threshold (mPa.s)
Yellow pea	0.85	97	0.63	93	226
Lupine seed	0.94	98	0.84	95	412
All data	0.82	96	0.82	94	158

#### 3.2.2 Models fitted on datasets from individual crops

The relations for each techno-functional property based on datasets from individual crops were quantified using a regularized polynomial linear regression, spline regression, random forest, and neural network. These models were found to have best prediction metrics as well as the most physically feasible behaviour. The models fitted from yellow pea ingredients for the emulsion stability as well as the lupine seed ingredients for heated viscosity benefited from splitting the independent variable protein according to their solubility. A considerable number of models have a stable prediction, which means that the train and test errors are close to each other. For example with the heated viscosity and emulsion stability. In some cases, the test error is considerably higher than the training error, for example in the case of foaming capacity. The parity plots in Fig. 4 indicate that the foaming capacity is overestimated at lower concentrations and underestimated at higher concentrations for yellow pea and (especially) lupine seed ingredients. The plotted behaviour of the random forest for foaming capacity also show discontinuous behaviour, which is undesired. Yet, there is at this point no better alternative. Besides the foaming capacity, the parity plots depict that all other techno-functional properties are still quantified within an acceptable range using the selected models for the individual crops.

#### 3.2.3 Models fitted on combined dataset

Next, the observations of yellow pea and lupine seed, as well as mixtures of those were combined and modelled together. As the same component from different crops can have different contributions to the final functional properties, we also considered whether we would need to split protein and fibre according to their crop origin as input variables (e.g., protein from yellow pea and lupine seed). We focus only on protein and fibre since starch is only present in considerable concentrations in yellow pea ingredients. In addition, no large structural contribution from the rest fraction is expected as these are mostly well-soluble solids.

Overall, the prediction error of some models fitted on all data combined is in the same range as the error from the models fitted on data from single crops only (Fig. 3). For example, in the case of foaming capacity of both datasets of yellow pea and lupine seed ingredients. For other properties, the model errors are considerably worse when fitting based on combined datasets from yellow pea and lupine seed ingredients. Splitting protein and fibre according to crop origin improved the prediction error in some cases, for example for the unheated viscosity of yellow pea ingredients. As a result, the errors of the models based on the combined datasets are now in almost all cases in the same range as the models based on the individual datasets, with or without split of protein and fibre according to their origin. This is not the case for the heated viscosity, for which the error is substantially higher when modelling a combined dataset compared to an individual. This is attributed to the poor prediction of observations that were based on mixtures of yellow pea and lupine seed ingredients.

**Fig. 5:**
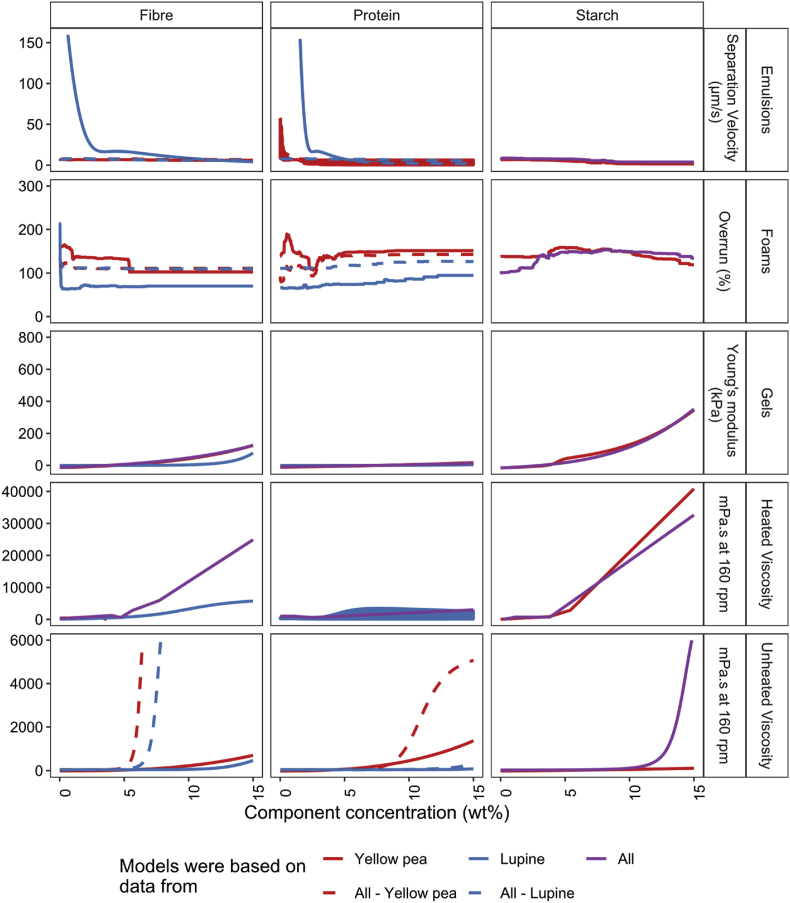
Behaviour of each component in the fitted models based on yellow pea, lupine seed, or all (with or without split in protein and fibre). The models were used to predict theoretical datasets in which each component increases from 1 to 15wt% while the remaining component are set constant to 2wt%. In case a model required the soluble and insoluble protein is as input variables, the solution space is given as shaded area. (For interpretation of the references to colour in this figure legend, the reader is referred to the Web version of this article.)

Yet, based on the model metrics, it is not clear which independent variables are most suitable since the model metrics do not consistently improve when predicting all three datasets using combined models with a split according to crop origin. To facilitate the decision on a final model, the behaviour of each component in each model is evaluated. The trends of the fitted individual models were revealed using a theoretical dataset, in which one component increases, while the other components remain constant at 2wt% (Fig. 5) (Lie-Piang et al., 2023). From this Figure, it can be derived that protein and fibre in lupine seed have a very different contribution to the foaming capacity, and to a lesser extent for unheated viscosity and emulsions stability. Protein and fibre from yellow pea and lupine seed have a similar contribution to the gel stiffness and heated viscosity. Therefore, it is suggested that the most suitable models for the emulsion stability, foaming capacity, and unheated viscosity are based on independent variables that are split according to crop origin. The behaviour of the models based on all data (split or without split) is also plotted in Fig. 5.

In summary, models can be fitted such that they predict the selected techno-functional properties of both yellow pea and lupine seed samples and mixtures of those although with sometimes a reduction in model accuracy. The decision on the most suitable model is also not always straightforward as there can be sets of variables that result in the same range regarding the metrics. Considering the behaviour of the components in each model as well can aid in selecting the right predictive variables.

## 4 Discussion

### 4.1 Reflection on the independent variables

Most of the selected techno-functional properties could be described using the main macro components as input variables. This implies that the effect of the processing history (i.e. conventionally or mildly fractionated) on the state of the protein and functional property is negligible compared to other components or effects (i.e. interactions). For gelation and heated viscosity this was expected due to the dominance of starch gelatinisation (Singh, 2020, para. 5.5.1). A difference was expected based for the foaming capacity, emulsion stability, and unheated viscosity. We hypothesize that due to the large range of measured components and hence, the large range of for example foaming capacities, these differences become negligible. Another effect may be that to measure some properties, the product is heated. This eliminates any differences in the degree of denaturation of the proteins, in case of yellow peas above about 83 °C (Kornet et al., 2021; Pelgrom et al., 2015). In addition, the protein from the mildly refined protein-rich fraction is only present at lower purity (<55 wt%), and therefore at these low concentrations other components and/or their interactions could be dominant.

The models that were fitted on the combined datasets of yellow pea and lupine seed ingredients as well as mixtures of those had in most cases a similar prediction error to the models based on individual crops. Based on the model metrics and the behaviour plots, we suggest that the foaming capacity, emulsion stability, and unheated viscosity benefit from the separation of the independent variables according to the crop origin. These three properties were all based on samples containing unheated powders. As opposed to the heated samples, e.g. viscosity, the unheated viscosity is mainly dependent on the size of the protein aggregates (Kornet et al., 2020) and the water holding capacity of the protein (Osen et al., 2014). With an increase in fibre the amount of free water is also reduced, which increases the viscosity (Pelgrom et al., 2015). Since these properties can differ among different legumes, a split may be required. In contrast, starch gelatinisation is dominant in the observations of heated viscosity and gelation. As starch is present only in very low concentrations in lupine seed, it is hypothesized that there is no need for a split in components according to the source.

The results indicate that not all techno-functional properties could be predicted from the same variables. In the case of two crops the independent variables can still be split, as was indeed done in this study. Yet, expansion of these models to more than two crops leads to a large number of variables. Even though some machine learning algorithms can cope with a large number of variables, it can be of more interest to investigate whether another approach could be of value. For example, one could identify an intermediate layer of physical properties that would have a better, more generic relation with the ultimate technical functionalities. Description of the functional properties by their physical properties such as the water holding capacity or particle size, would make the formulation of products more generic, than using the conversion to composition and back, which is what we applied here.

### 4.2 Quality of the model predictions

Although the training errors from the fitted models on all data are within a reasonable range, the test errors are considerably higher for some functional properties. While it is common that the test error is larger than the training error (James et al., 2021), a large difference could point to an overfitted model. For example, the RMSE of the test set of emulsion stability is more than twice as large as the RMSE of the training set. This means that the trained models may not be suitable to generalize to other unseen data. This can be the result of having too many predictive independent variables or having too complex models (e.g., too many neural nodes or high degree polynomial) (Ghojogh and Crowley, 2019; James et al., 2021). Another reason could be that there is not enough data to train the model properly (Hawkins, 2004). However, having too complex models is most likely not relevant here since we carefully reduced the model complexity by using cross validation during model training. The latter reason could be more relevant in this case. Although the total sizes of the datasets are fairly large, the test sets contain in some cases up to 40% of all observations. This leaves us with a relatively sparse training. Therefore, we evaluated whether the size of the dataset is sufficient.

We assessed the model performance of data subsets with sizes ranging from 10 to the total number of observations that were measured in this study. Due to the small size of the datasets, we applied a nested leave-one-out cross-validation (LOOCV). In this way, every sample becomes a test sample one time while the other observations are used to train a model. The model performance was expressed with Q^2^, which is similar to the R^2^ of the test value but calculated with the equation that expresses the explained variance of the residuals (Equation (3)). The LOOCV can be known for overfitting due to the large training set as opposed to the test set (Kohavi, 1995). As a control, we have plotted the Q^2^ of the original division in the training and test set (up to 40% test set) from this study.

**Fig. 6:**
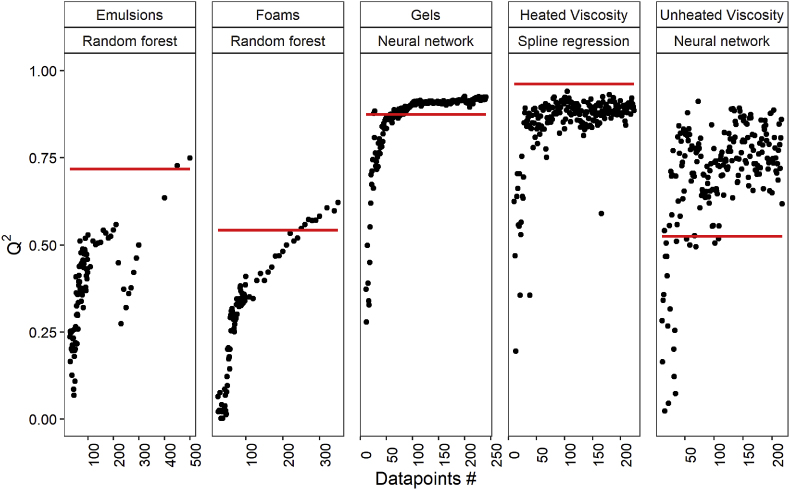
Accuracy (Q^2^) of models generated for each functional property with a dataset size ranging from 10 to the maximum number of observations measured. The red line indicates the Q^2^ of the original test dataset used in this study. (For interpretation of the references to colour in this figure legend, the reader is referred to the Web version of this article.)

Fig. 6 shows that with the addition of more observations the prediction quality of emulsion stability and foams keeps on increasing. This means that indeed the dataset size for these was not sufficient. At around 227 observations, there is a sudden decline in Q^2^. A closer look into the additional observation added here (observation nr. 227) shows that this point is related to a relatively high emulsion separation velocity of 168 μm/s, while the observations before this point had a maximum separation velocity of 112 μm/s. We expect that this magnitude of separation velocity is undertrained and therefore reduces the overall accuracy of the model. After the addition of the 227th point, the accuracy starts to improve again. Therefore, besides having a homogenous distribution of the compositional space, the distribution of the output variables should also be homogenous.

The Q^2^ of the prediction for gel stiffness as well as heated and unheated viscosity reaches a plateau after around 100 observations. As the Q^2^ for the prediction of gel stiffness and heated viscosity is quite high already (>0.8), we conclude that we do not always need the complete dataset to achieve this model performance. If the train error is still a lot lower than the test error, this could be the result of unsuitable or too many predictive variables. The Q^2^ of the unheated viscosity reaches a plateau, yet the variation of the Q^2^ is large. This could indicate that the data of the viscosity is not spread homogenously either among the compositional space or in terms of the output, in this case, final viscosity. Since the plateau from the LOOCV is slightly higher than the one of the original dataset, there is a chance that the LOOCV slightly overestimates the Q^2^. This means that the Q^2^ of the plateau in fact could be slightly lower.

In summary, some overfitting takes place in the fitted models in this study which can be attributed to a lack of samples in the case of emulsion stability and foaming capacity. The analysis for emulsion stability also showed that besides having well distributed data over the composition space, it is also important to have a proper distribution in the model output, in this case, separation velocity. For the other properties overfitting can be attributed to for example the selection of predictive variables. Using intermediate physical properties as independent variables can result in models with lower overfitting and possible fewer samples. We suggest that when measuring the functional properties of a new crop, analyses like these should be executed in parallel to the experimental measurements, such that the experimental work can be stopped as soon as the plateau is reached. This could ultimately be paired with an automated sampling method.

## 5 Conclusions

The usage of milder refined ingredients into food formulations is facilitated by selection based on techno-functional properties rather than composition. The properties that are important in food product formulations were therefore quantitatively modelled using machine learning. These properties were the thickening behaviour (viscosity and gelling), emulsions stability, and foaming capacity. We evaluated whether a single model could predict these properties from multiple crops. The models were therefore fitted on individual datasets from yellow pea and lupine seed ingredients as well as on a combined dataset that contained both ingredients from yellow pea and lupine seed as well as mixtures of those two.

The relations between ingredients from single crops and their functional properties were described using a regularized polynomial linear regression, spline regression, random forest, and neural network. The latter three models were selected to predict the functional properties from all data of yellow pea, lupine seed, and mixtures of those. For some properties, it was necessary to split the protein and fibre according to the origin of the crop to obtain better model performance and physically plausible behaviour of each component. The test errors of the models based on the combined datasets were in the same range as those of the models based on individual datasets. Only the heated viscosity was predicted worse using a model based on combined data. Some overfitting was detected in the fitted models, which was later attributed to a lack of samples or the selected predictive variables. This analysis indicated as well that not all observations were necessary predict all properties.

This study demonstrates the possibility to extend the functionality-driven selection of food products to multiple crops and presents a method to evaluate the relevance of increasing the size of the dataset. The presented method aids in designing food formulations that may help in significantly reducing the overall chain environmental impact of the production of the foods, by reducing the amount of refining necessary to acquire the right product properties. Future studies should focus on improving the accuracy of these models by exploring other predictive variables as well as improving sample selection (e.g. using Kennard Stone) and collection. Ultimately these models should be extended to more complex food products that also include other conditions like salt or pH. The latter can be facilitated by striving for better genericity by using hybrid models that use a combination of mechanistic and empirical models.

## Credit author statement

Anouk Lie-Piang: Conceptualization, Methodology, Formal analysis, Validation, Software, Investigation, Visualization, Writing – Original Draft.

Jos Hageman: Conceptualization, Formal analysis, Writing – Review and Editing,

Iris Vreenegoor: Methodology, Investigation.

Kai van der Kolk: Methodology, Investigation.

Suzan de Leeuw: Methodology, Investigation.

Remko Boom: Supervision, Conceptualization, Visualization, Writing – Review and Editing, Project administration.

Albert van der Padt: Supervision, Conceptualization, Visualization, Writing – Review and Editing, Project administration, Funding acquisition.

## Declaration of competing interest

The authors declare no conflict of interest.

## Figures and Tables

**Table A 1:** Overview of the ingredients used in this study.

	Crop	Commercial name	Source	Processing procedure of in-house produced ingredients.
Yellow pea protein isolate (YPI)	Yellow pea (*Pisum sativum L.)*	Nutralys F85G	Roquette Frères S.A (St. Louis, USA)	–
Yellow pea starch isolate (YSI)	Yellow pea (*Pisum sativum L.)*	Erbenstärke Premium Qualität	Emsland Stärke GmbH (Germany)	–
Yellow pea fibre isolate (YFI)	Yellow pea (*Pisum sativum L.)*	PEA FIBRE I 50 M	Roquette Frères S.A (St. Louis, USA)	–
Yellow pea fine fraction (YFF)	Yellow pea (*Pisum sativum L.)*	–	Peas delivered by Alimex (The Netherlands)	Peas were dehulled, milled into a flour, and separated by air classification into a fine and coarse fraction.
Yellow pea coarse fraction (YCF)	Yellow pea (*Pisum sativum L.)*	–	Peas delivered by Alimex (The Netherlands)	Peas were dehulled, milled into a flour, and separated by air classification into a fine and coarse fraction.
Yellow pea flour (YPF)	Yellow pea (*Pisum sativum L.)*	–	Peas delivered by Alimex (The Netherlands)	Peas were dehulled and milled into a flour.
Lupine protein isolate (LPI)	Lupine seeds (*Lupinus angustifolius*)	Lupine protein isolate	Prolupin GmbH (Germany)	–
Lupine fibre isolate (LFI)	Lupine seeds (*Lupinus angustifolius*)	–	Prolupin GmbH (Germany)	Slurry was freeze dried and milled.
Lupine flour (LF)	Lupine seeds (*Lupinus angustifolius*)	–	Seeds delivered by InvejaFood (The Netherlands)	Seeds were milled into a flour. Large pieces of hulls were removed by sieving.
Lupine fine fraction (LFF)	Lupine seeds (*Lupinus angustifolius*)	–	Seeds delivered by InvejaFood (The Netherlands)	Seeds were milled into a flour and separated by air classification into a fine and coarse fraction. Large pieces of hulls were removed by sieving.
Lupine coarse fraction (LCF)	Lupine seeds (*Lupinus angustifolius*)	–	Seeds delivered by InvejaFood (The Netherlands)	Seeds were milled into a flour and separated by air classification into a fine and coarse fraction. Large pieces of hulls were removed by sieving.

**Fig. A 1:**
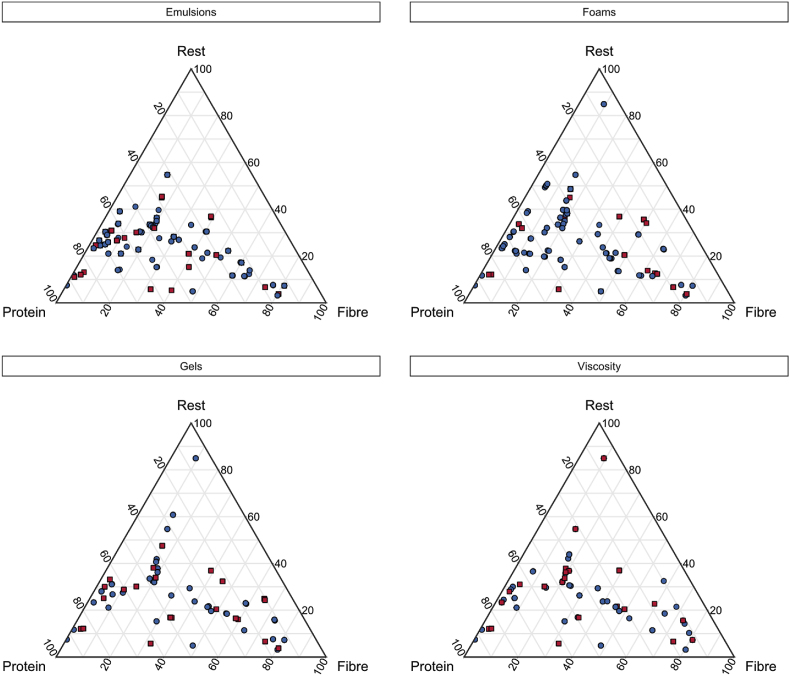
Ternary plots for samples (ratio protein, starch, and rest) of all techno-functional properties with train (●) and test (■) data points All determined in a range of 1–25 wt%.

**Table A 2:** Model metrics models for quantifying unheated viscosity with main macro components as independent variables for lupine seeds ingredients.

Model	RMSE Train	R2 Train	MAE Train	RMSE Test	R2 Test	MAE Test
Linear Model	494.73	0.80	297.56	482.37	0.59	333.00
Log Linear Model	721.84	0.59	179.01	295.52	0.65	94.52
Poly Model	212.58	0.96	158.54	349.74	0.78	215.63
Regularisation Model	445.65	0.84	214.43	467.02	0.64	278.70
Spline Model	220.66	0.96	124.12	352.40	0.68	201.38
Random Forest	308.43	0.95	92.37	344.78	0.80	130.84
Neural network	143.59	0.98	77.99	248.52	0.92	115.63

**Fig. A 2:**
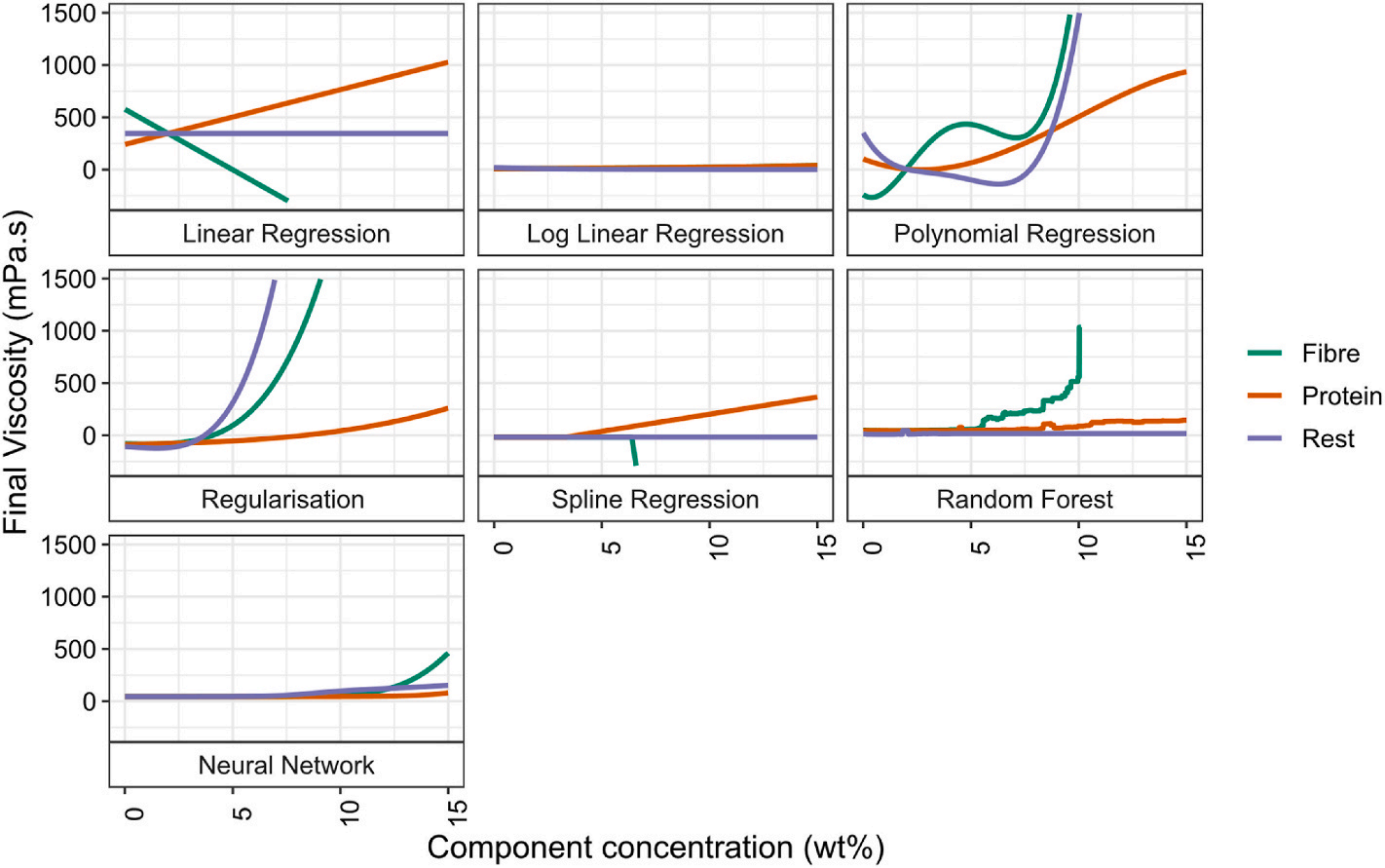
Scatterplot of the behaviour of each component in the evaluated models for quantifying the unheated viscosity of lupine seed ingredients with the main macro components as independent variables. The composition of each component increases from 1 to 15 wt% while the other stay constant at 2%.

**Fig. A 3:**
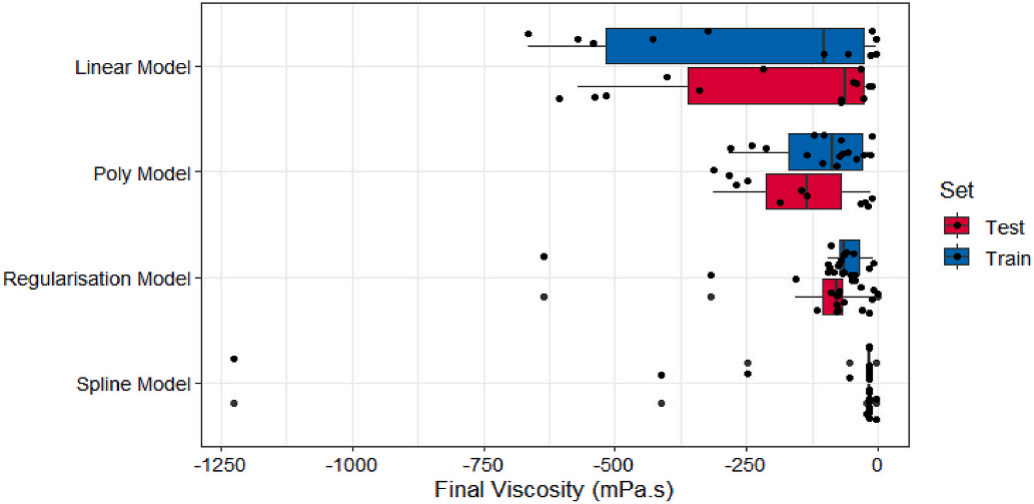
Boxplot of negative values predicted by the evaluated models to for quantifying the unheated viscosity of lupine seed ingredients with the main macro components as independent variables.

**Fig. A 4:**
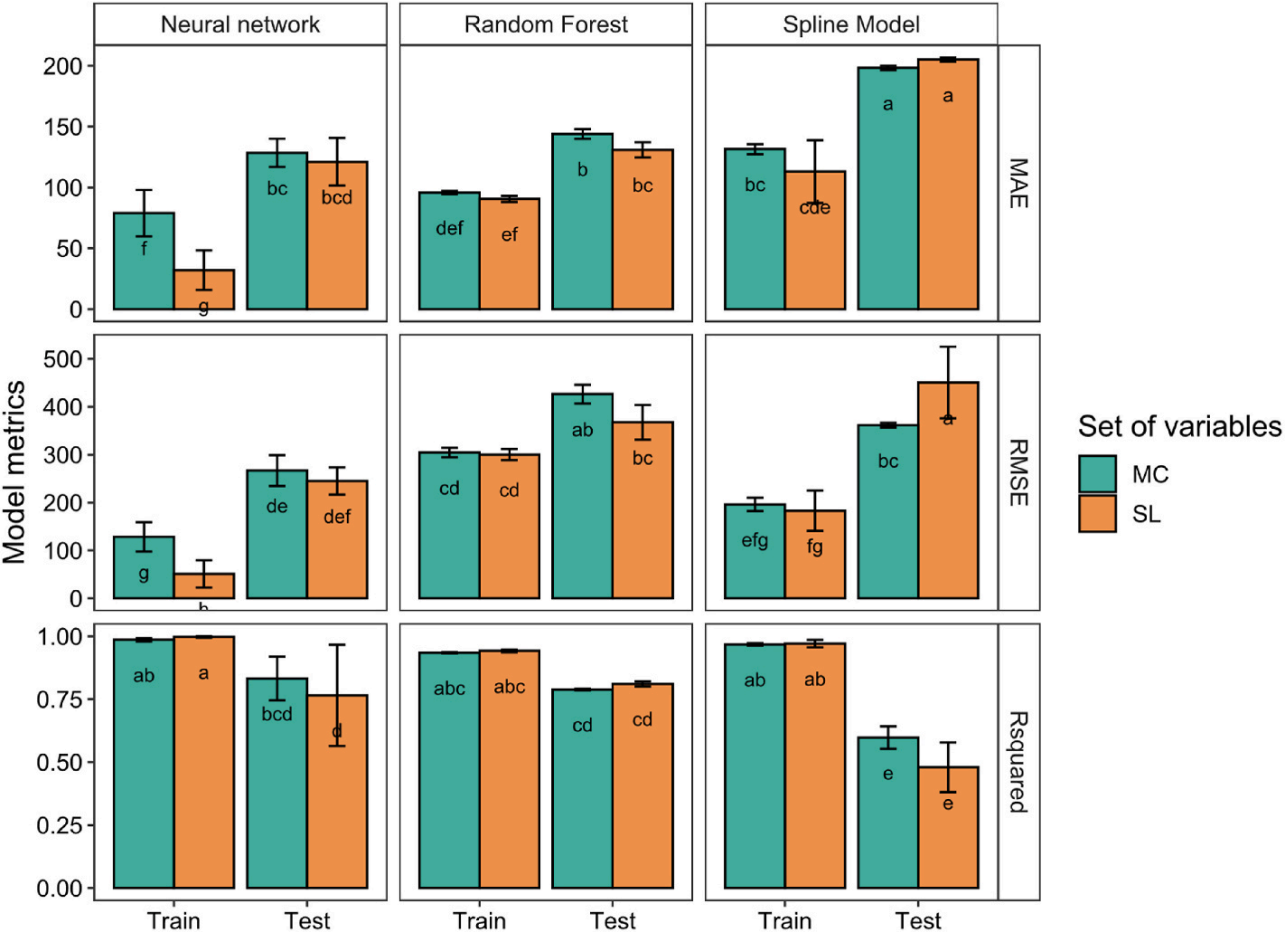
Bar chart containing the model metrics to predict the unheated viscosity (mean absolute error (MAE), root mean square error (RMSE), and R^2^) generated five times for the neural network, random forest, and spline regression for lupine seed ingredients with the main macro components (MC) and main macro components with a split according to native proteins (ND) and soluble protein (SL) as independent variables. Letters indicate a significant different (P < 0.05).

**Table A 3:** Summary and metrics (RMSE, R^2^, and MAE) of models selected to quantify the relation between the ingredients and techno-functional properties with the main macro components (MC) or with the main macro components with the protein split according to solubility (SL) as input variables. The tuned hyper parameters are indicated for the random forest (# predictors used), neural network (hidden nodes in layer 1, layer 2, layer 3), spline regression (number of splines and the degree), and the regularized polynomial regression (alpha and lambda). The split column indicates whether models containing data from all crops included a split in independent variables according to crop.

Functional property	Crop	Best model	Tuned hyperparameters	Variable set	Split	RMSE Train	R^2^ Train	MAE Train	RMSE test	R^2^ Test	MAE Test
Emulsion stability	Yellow pea	Random forest	3 predictors	SL	NA	3.03	0.99	1.6	4.3	0.88	2.71
	Lupine seed	Neural network	1, 2, 0 hidden nodes	MC	NA	7.66	0.9	5.21	11.72	0.87	7.25
	All	Random forest	4 predictors	MC	Yes	4.53	0.97	2.49	17.38	0.62	7.72
Foaming capacity	Yellow pea	Random forest	3 predictors	MC	NA	15.96	0.94	11.7	38.29	0.65	26.84
	Lupine seed	Random Forest	3 predictors	MC	NA	27.48	0.9	17.95	71.83	0.18	56.77
	All	Random Forest	3 predictors	MC	Yes	20.16	0.94	14.02	47.66	0.55	35.2
Gelation	Yellow pea	Neural network	2, 3, 0 hidden nodes	MC	NA	31.82	0.97	22.57	64.6	0.88	42.05
	Lupine seed	Neural network	1, 1, 0 hidden nodes	MC	NA	5.54	0.99	3.27	15.14	0.92	7.52
	All	Neural network	1, 2, 0 hidden nodes	MC	No	36.88	0.96	26.7	54.87	0.89	33.63
Heated viscosity	Yellow pea	Spline regression	15 splines, 2^nd^ degree	MC	NA	420.6	0.98	234.3	597.53	0.95	457.04
	Lupine seed	Neural network	3, 1, 0 hidden nodes	SL	NA	353.93	0.96	224.5	547.07	0.93	280.73
	All	Spline regression	20 splines, 2^nd^ degree	MC	No	822.83	0.94	554.89	1960.03	0.96	1076.79
Unheated viscosity	Yellow pea	Regularized polynomial regression	alpha = 0.1 and lambda = 12.4.	MC	NA	52.47	0.71	21.95	69.86	0.99	36.05
	Lupine seed	Neural network	3, 3, 0 hidden nodes	MC	NA	143.59	0.98	77.99	248.52	0.92	115.63
	All	Neural network	2, 1, 0 hidden nodes	MC	Yes	102.93	0.98	32.29	203.66	0.79	110.86

**Fig. A 5:**
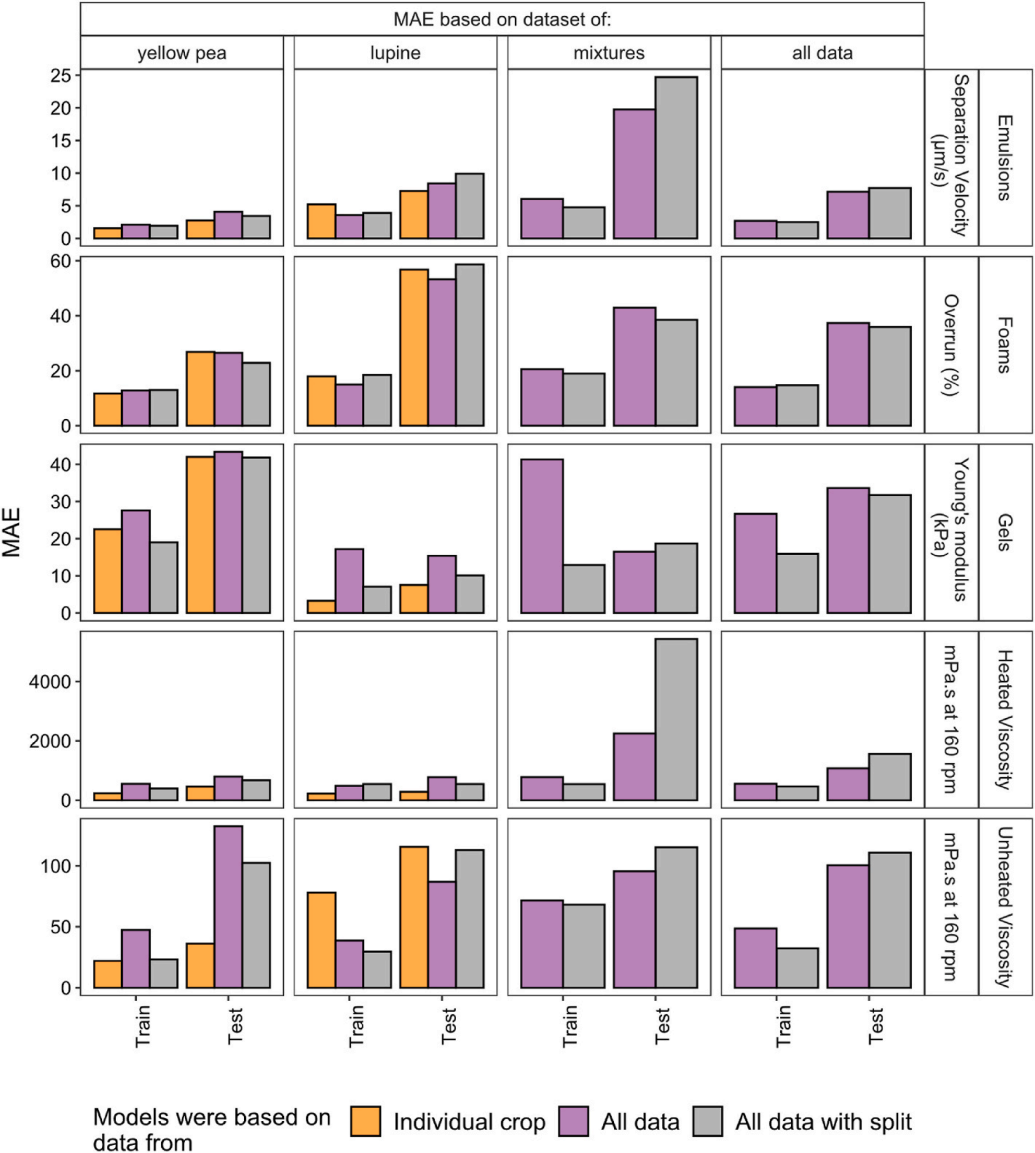
MAE of the models based to predict the emulsion separation velocity (μm/s), foam overrun (%), gel Young's modulus (kPa), heated and unheated viscosity (mPa.s). Models were trained on the data from individual crops (yellow pea and lupine seed) and on all data (yellow pea, lupine seed, and mixtures of those) and on all data with a split in protein and fibre according to origin. The RMSE is calculated for the samples of yellow pea, lupine seed, mixtures, and all data together.

**Fig. A 6:**
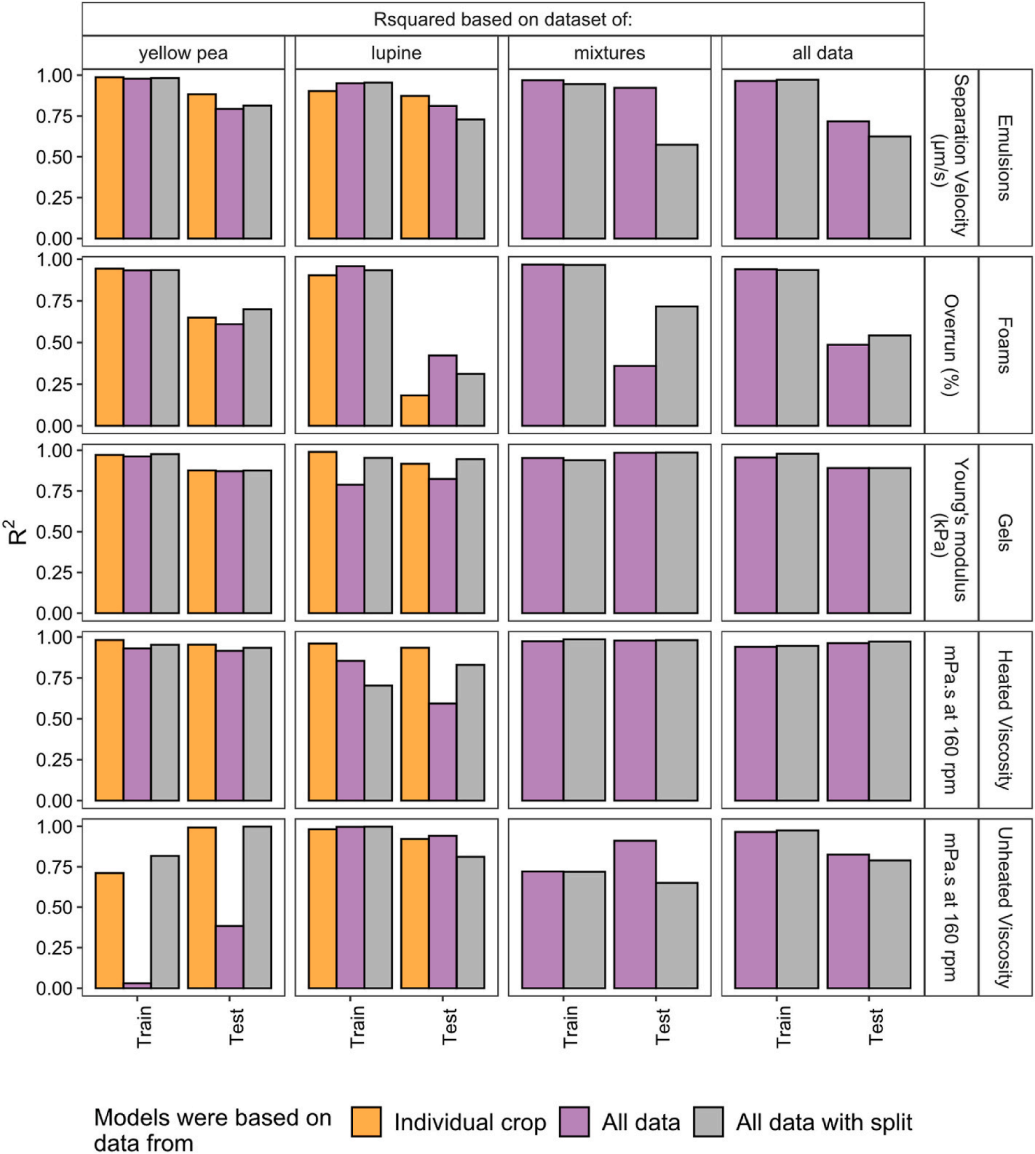
R^2^ of the models based to predict the emulsion separation velocity (μm/s), foam overrun (%), gel Young's modulus (kPa), heated and unheated viscosity (mPa.s). Models were trained on the data from individual crops (yellow pea and lupine seed) and on all data (yellow pea, lupine seed, and mixtures of those) and on all data with a split in protein and fibre according to origin. The RMSE is calculated for the samples of yellow pea, lupine seed, mixtures, and all data together.

## Data Availability

Data will be made available on request.

## Appendix

### A.3 Example of model selection unheated viscosity of lupine ingredients

From the model metrics it is derived that the neural network is the best candidate for quantifying unheated viscosity of lupine ingredients. The behaviour scatterplots shows physically plausible behaviour in terms of trends and negative values for the neural network. There are no significant differences between the test metrics between the base components and the split according to solubility. Therefore, the best model is the neural network with main macro components. As there is quite a large variation in the five times repeated neural networks, the one with the lowest test error is chosen for further analyses.

For the other properties we refer to the supplementary information.
