# Supplementary material for: Quantifying techno-functional properties of ingredients from multiple crops using machine learning
Source: Curr Res Food Sci. 2023 Sep 22;7:100601. doi: 10.1016/j.crfs.2023.100601 (PMC10562757; doi:10.1016/j.crfs.2023.100601)
Supplement: Multimedia component 1 [file mmc1.docx]

## Supplement to manuscript entitled “Quantifying techno-functional properties of ingredients from multiple crops using machine learning”

Anouk Lie-Piang, Jos Hageman, Iris Vreenegoor, Kai van der Kolk, Suzan de Leeuw, Albert van der Padt , Remko Boom

## Emulsions

### Yellow pea

The spline regression, random forest, and neural network can all quantify the emulsion separation of velocity yellow pea ingredients accurately based on the test metrics. The neural network and spline model predict a few negative values for protein in the behaviour. When looking at the repetitions, the random forest performs better based on the train and test metrics than when the protein is split according to processing history. Therefore, a random forest with a split according to solubility was chosen.

Table 1 Model metrics models for quantifying emulsion stability with main macro components as independent variables for yellow pea, lupine ingredients and mixtures of those.

| Model | RMSE Train | R2 Train | MAE Train | RMSE Test | R2 Test | MAE Test |
| --- | --- | --- | --- | --- | --- | --- |
| Linear Model | 20.21 | 0.31 | 14.50 | 12.77 | 0.31 | 9.82 |
| Log Linear Model | 20.56 | 0.42 | 11.48 | 9.70 | 0.42 | 5.45 |
| Poly Model | 17.08 | 0.51 | 12.08 | 10.50 | 0.51 | 7.83 |
| Regularisation Model | 17.21 | 0.50 | 11.77 | 10.05 | 0.51 | 7.29 |
| Spline Model | 6.03 | 0.94 | 4.09 | 5.89 | 0.82 | 4.13 |
| Random Forest | 3.54 | 0.98 | 1.91 | 5.58 | 0.81 | 3.35 |
| Neural network | 11.64 | 0.77 | 6.15 | 8.99 | 0.79 | 5.13 |

Table 2 Model metrics models for quantifying emulsion stability with main macro components as independent variables for yellow pea with a split in protein according to solubility.

| Model | RMSE Train | R2 Train | MAE Train | RMSE Test | R2 Test | MAE Test |
| --- | --- | --- | --- | --- | --- | --- |
| Linear Model | 20.20 | 0.31 | 14.56 | 12.90 | 0.30 | 9.97 |
| Log Linear Model | 20.49 | 0.44 | 11.26 | 9.61 | 0.44 | 5.36 |
| Poly Model | 15.63 | 0.59 | 11.11 | 10.98 | 0.50 | 8.55 |
| Regularisation Model | 16.28 | 0.56 | 10.93 | 9.82 | 0.53 | 7.01 |
| Spline Model | 6.00 | 0.94 | 4.16 | 4.27 | 0.90 | 3.41 |
| Random Forest | 3.03 | 0.99 | 1.60 | 4.30 | 0.88 | 2.71 |
| Neural network | 7.81 | 0.90 | 5.54 | 9.21 | 0.71 | 6.31 |


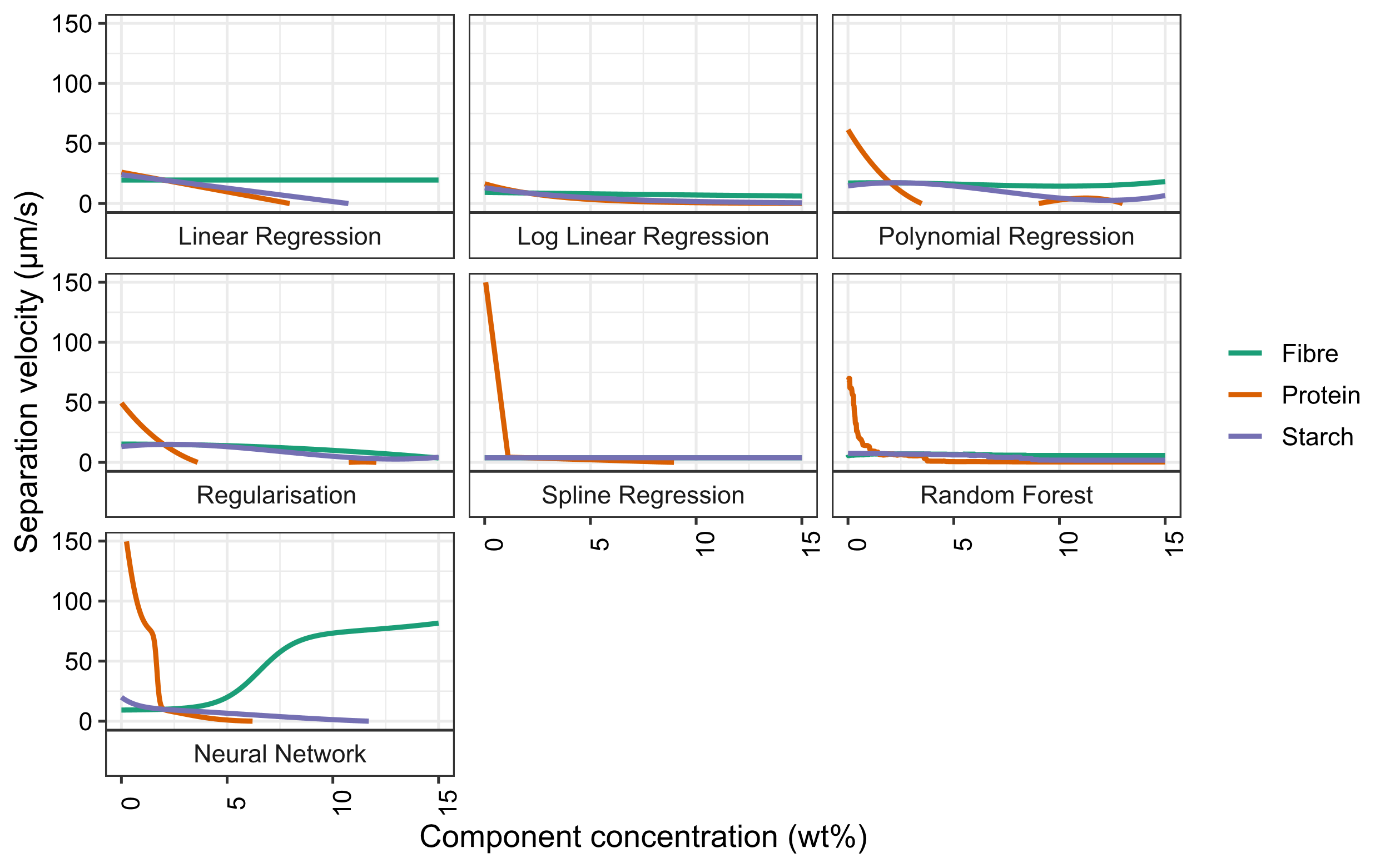


Figure 1 Scatterplot of the behaviour of each component in the evaluated models for quantifying the emulsion stability of yellow pea ingredients with the main macro components as independent variables. The composition of each component increases from 1-15 wt% while the other stay constant at 2%.


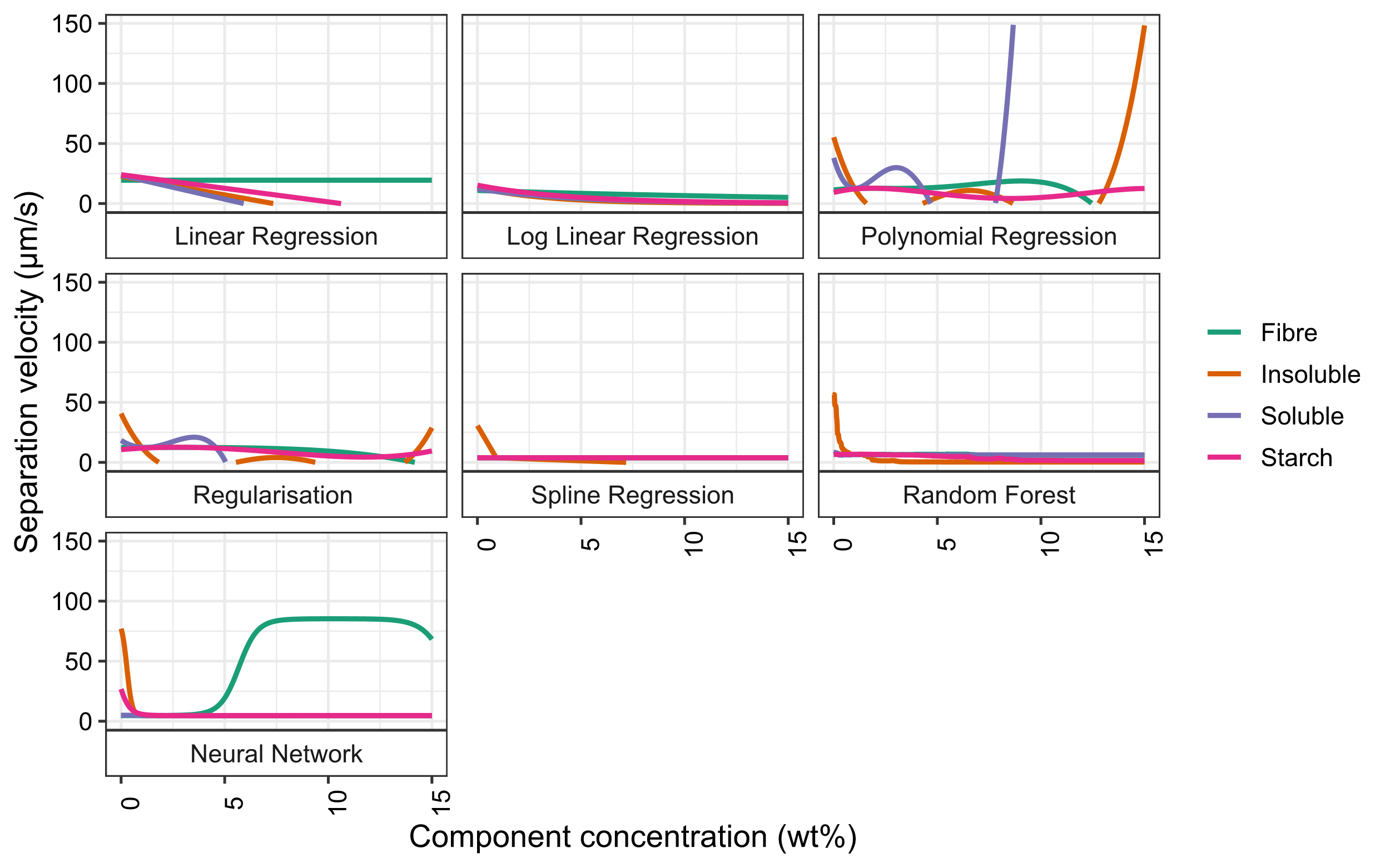


Figure 2 Scatterplot of the behaviour of each component in the evaluated models for quantifying the emulsion stability of yellow pea ingredients with the main macro components with a protein split according to solubility as independent variables. The composition of each component increases from 1-15 wt% while the other stay constant at 2%.


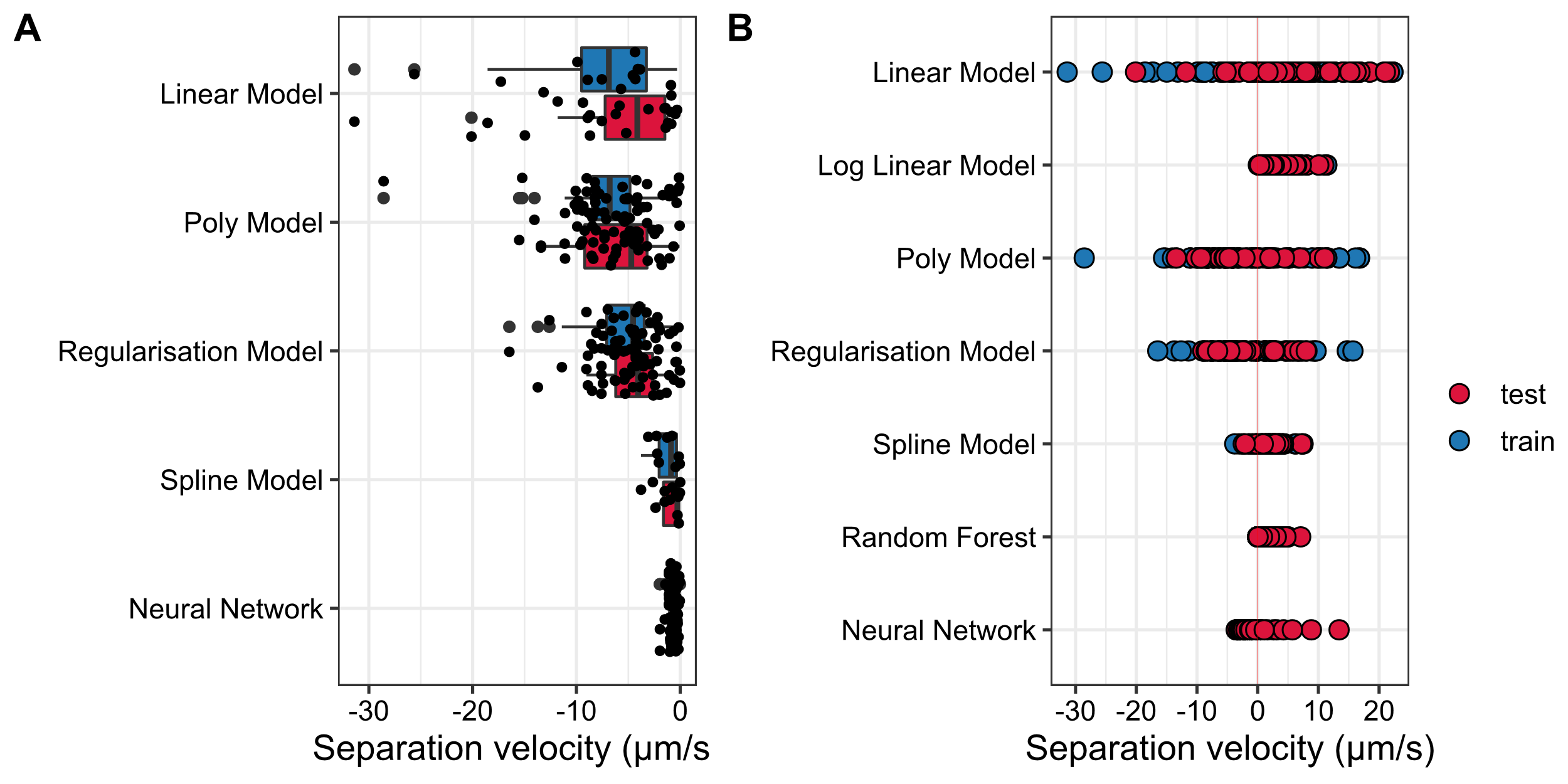


Figure 3 Box- and scatterplot wit negative values (A) and predicted values for the datapoints of with an original value of zero (B) predicted by the evaluated models for quantifying the emulsions stability of yellow pea ingredients with the main macro components as independent variables.


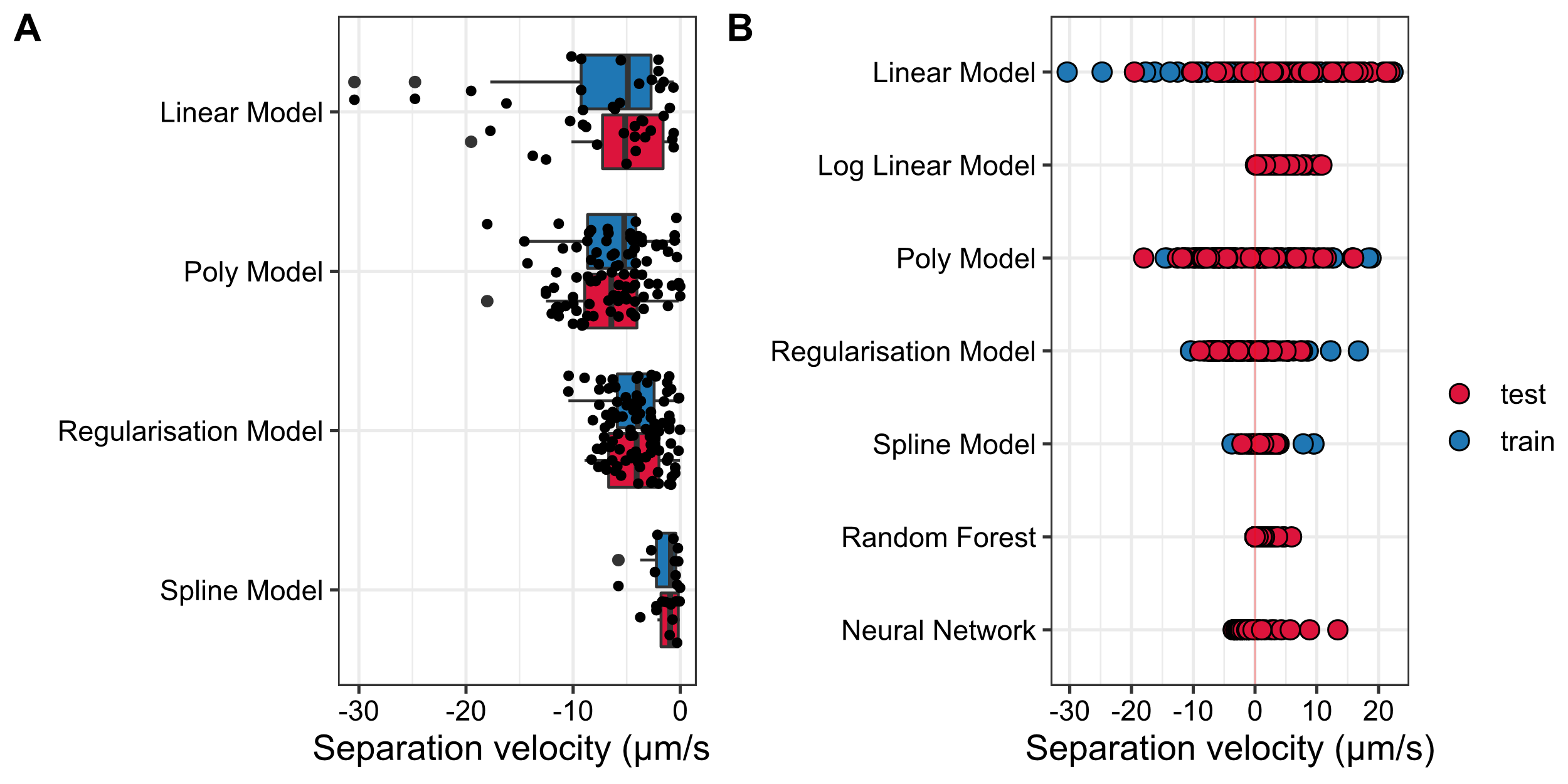


Figure 4 Box- and scatterplot wit negative values (A) and predicted values for the datapoints of with an original value of zero (B) predicted by the evaluated models for quantifying the emulsions stability of yellow pea ingredients with the main macro components with protein split according to solubility as independent variables.


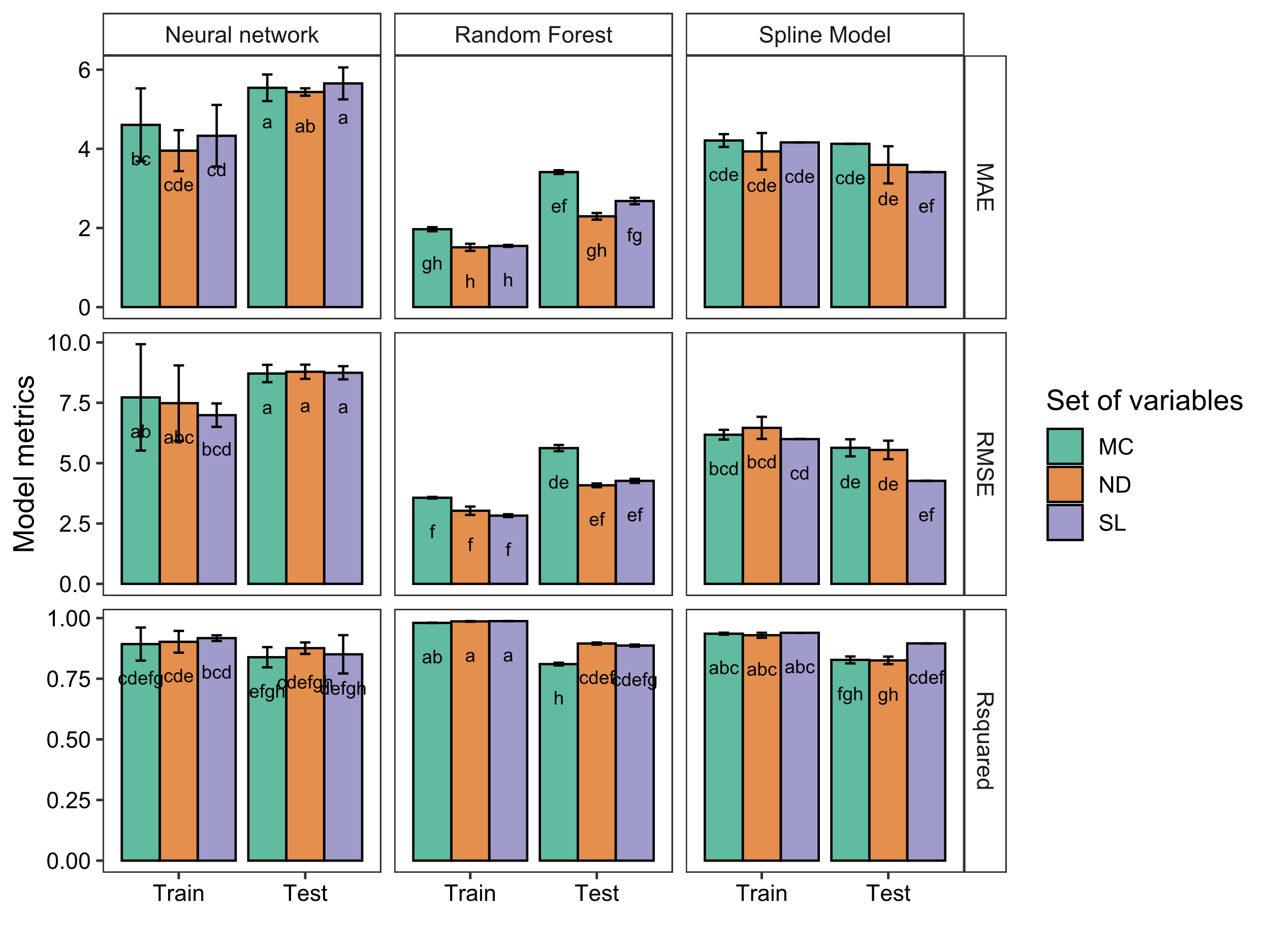


Figure 5 Bar chart containing the model metrics to predict the emulsion stability (mean absolute error (MAE), root mean square error (RMSE), and R^2^) generated five times for the neural network, random forest, and spline regression for yellow pea ingredients with the main macro components (MC) and main macro components with a split according to native (ND) and soluble protein (SL) as independent variables. Letters indicate a significant different (P<0.05).

### Lupine

The neural network can predict the emulsion stability of lupine ingredients with the lowest test metrics and has physically plausible predictions. No significant differences between the sets. Since there is quite a large variation between the repeated neural networks, the one that produces the lowest test errors is used for further analyses.

Table 3 Model metrics models for quantifying emulsion stability with main macro components as independent variables for lupine ingredients.

| Model | RMSE Train | R2 Train | MAE Train | RMSE Test | R2 Test | MAE Test |
| --- | --- | --- | --- | --- | --- | --- |
| Linear Model | 18.48 | 0.43 | 11.39 | 25.93 | 0.33 | 14.59 |
| Log Linear Model | 18.31 | 0.50 | 9.21 | 25.45 | 0.47 | 11.39 |
| Poly Model | 15.40 | 0.61 | 9.93 | 24.63 | 0.41 | 15.15 |
| Regularisation Model | 18.71 | 0.42 | 10.74 | 25.26 | 0.39 | 12.37 |
| Spline Model | 10.48 | 0.82 | 6.91 | 17.02 | 0.75 | 9.95 |
| Random Forest | 9.29 | 0.89 | 5.15 | 20.28 | 0.74 | 9.72 |
| Neural network | 7.66 | 0.90 | 5.21 | 11.72 | 0.87 | 7.25 |


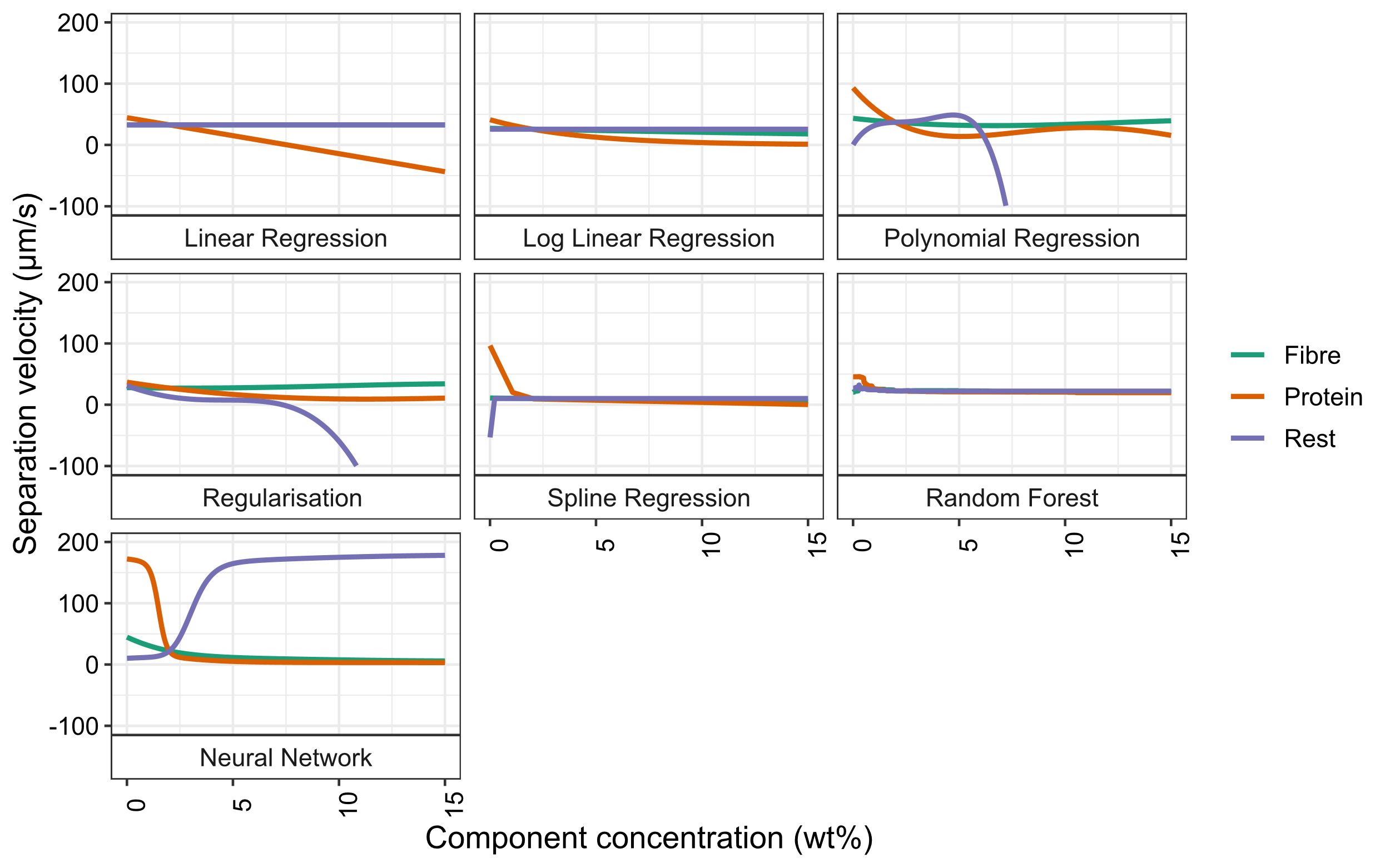


Figure 6 Scatterplot of the behaviour of each component in the evaluated models for quantifying the emulsion stability of lupine ingredients with the main macro components as independent variables. The composition of each component increases from 1-15 wt% while the other stay constant at 2%.


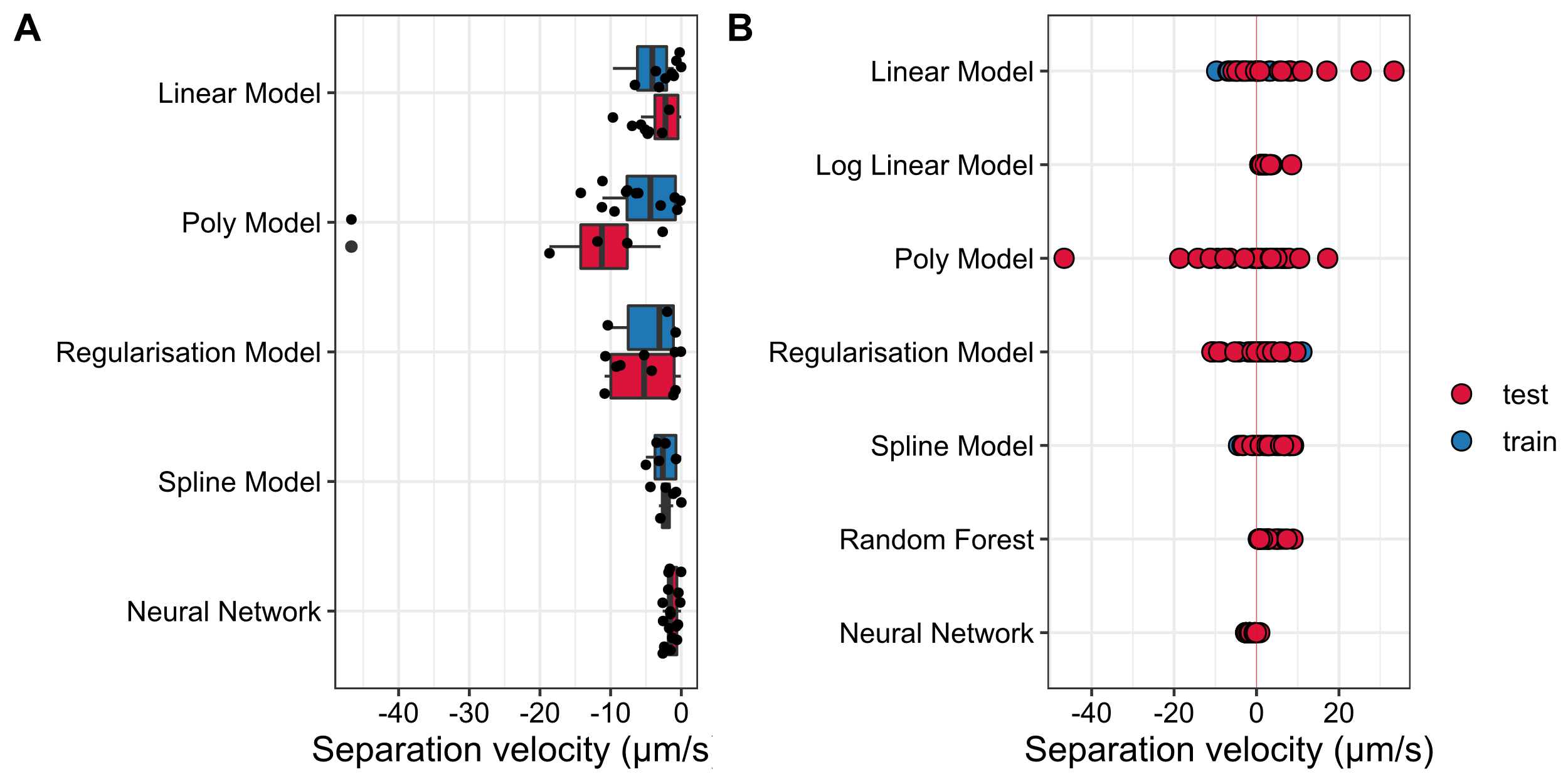


Figure 7 Box- and scatterplot wit negative values predicted by the evaluated models to for quantifying the emulsions stability of lupine ingredients with the main macro components as independent variables. Protein and fibre are split according to crop type.


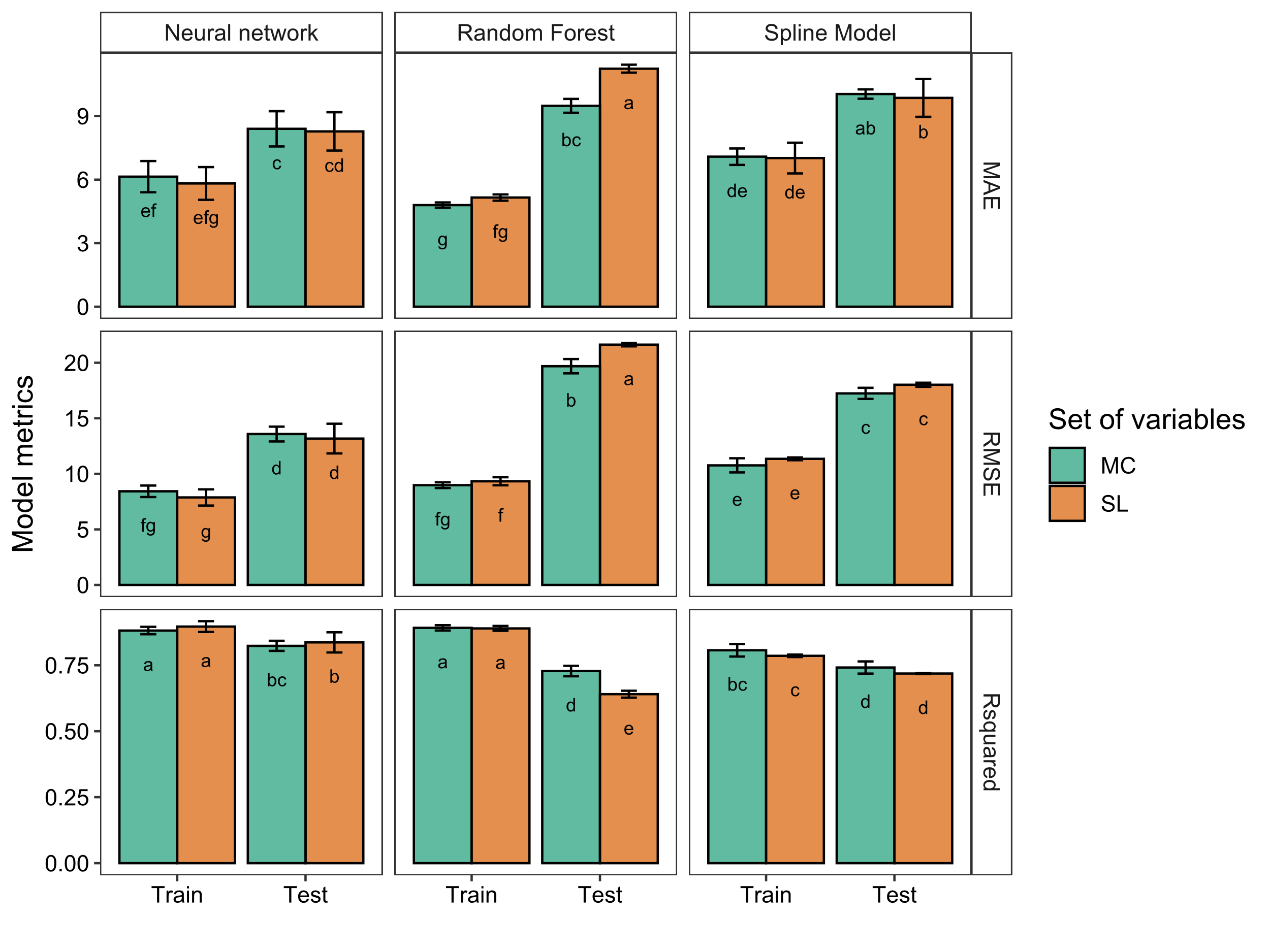


Figure 8 Bar chart containing the model metrics to predict the emulsion stability (mean absolute error (MAE), root mean square error (RMSE), and R^2^) generated five times for the neural network, random forest, and spline regression for lupine ingredients with the main macro components (MC) and main macro components with a split according to soluble protein (SL) as independent variables. Letters indicate a significant different (P<0.05).

### All data combined

To quantify the emulsions stability of yellow pea, a split in protein according to solubility as independent variables was required. Therefore, this split was also evaluated when quantifying the emulsion stability of yellow pea and lupine ingredients and mixtures of those together. However, the model types that can have variation, all do not show significant differences between the sets of variables, therefore the main macro components will be used in this case. The random forest is most feasible with this set of variables since it has acceptable model metrics and predict few negative values. It also shows a physically feasible behaviour.

Table 4 Model metrics models for quantifying emulsion stability with main macro components as independent variables for yellow pea, lupine, and mixtures of those.

| Model | RMSE Train | R2 Train | MAE Train | RMSE Test | R2 Test | MAE Test |
| --- | --- | --- | --- | --- | --- | --- |
| Linear Model | 20.52 | 0.32 | 14.57 | 24.12 | 0.19 | 14.25 |
| Log Linear Model | 20.99 | 0.40 | 12.03 | 24.19 | 0.26 | 10.43 |
| Poly Model | 19.42 | 0.39 | 13.52 | 22.65 | 0.28 | 12.21 |
| Regularisation Model | 19.46 | 0.39 | 13.38 | 22.74 | 0.28 | 11.95 |
| Spline Model | 9.74 | 0.85 | 6.58 | 14.58 | 0.72 | 7.75 |
| Random Forest | 5.03 | 0.96 | 2.69 | 15.79 | 0.72 | 7.15 |
| Neural network | 12.15 | 0.76 | 7.33 | 21.33 | 0.49 | 11.96 |


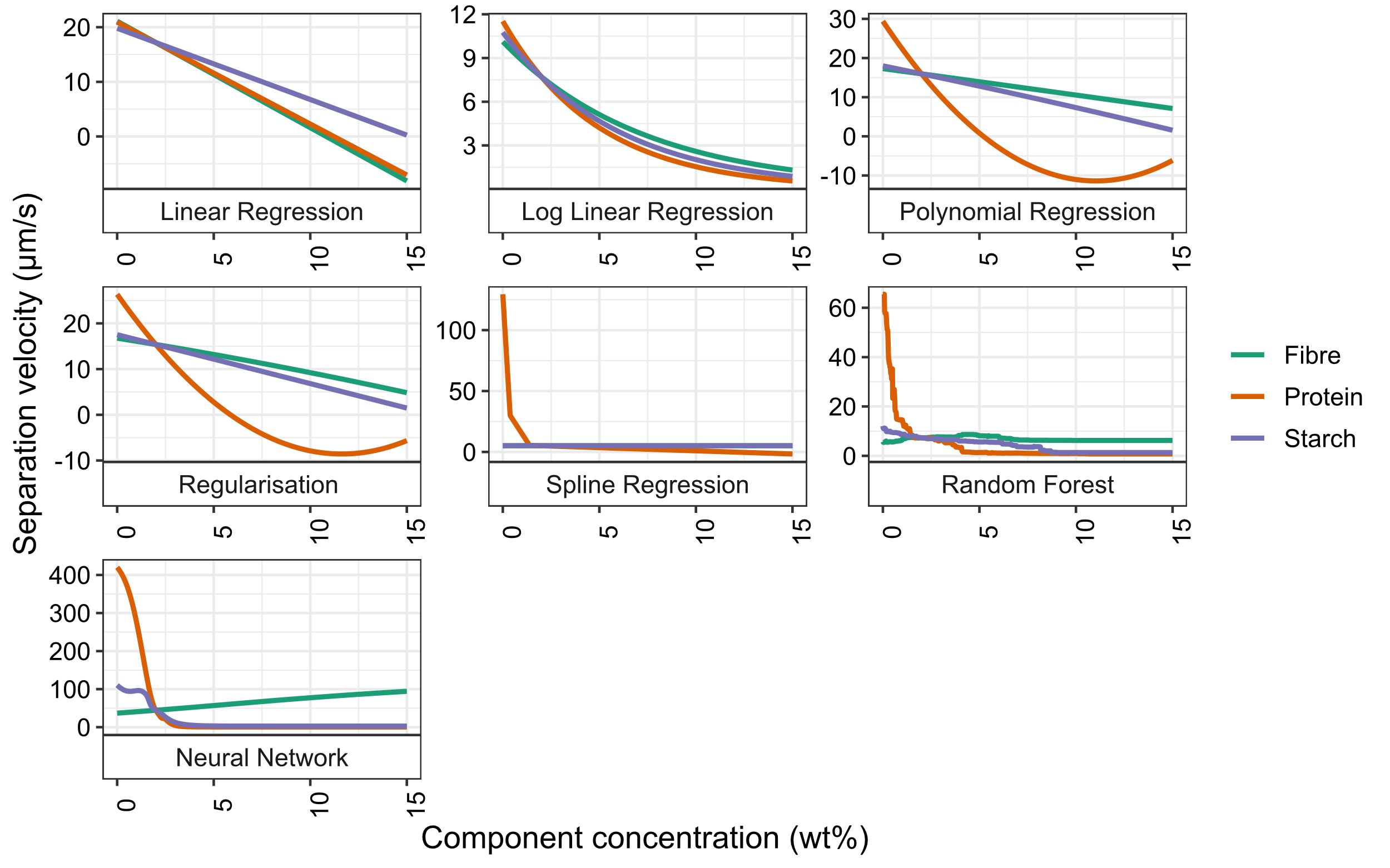


Figure 9 Scatterplot of the behaviour of each component in the evaluated models for quantifying the emulsion stability of yellow pea and lupine ingredients and mixtures of those with the main macro components as independent variables. The composition of each component increases from 1-15 wt% while the other stay constant at 2%.


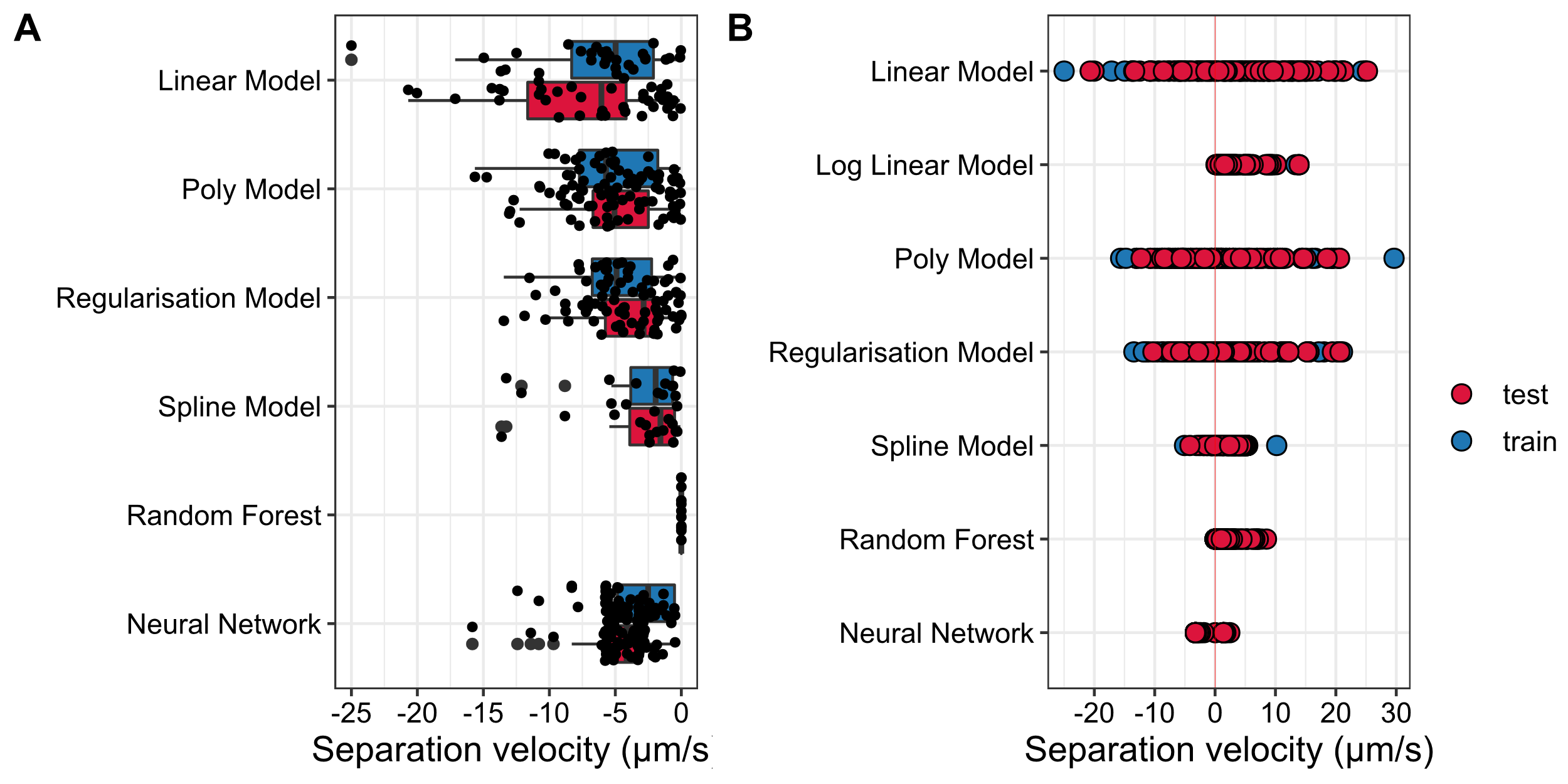


Figure 10 Box- and scatterplot wit negative values predicted by the evaluated models to for quantifying the emulsions stability of yellow pea and lupine ingredients and mixtures of those with the main macro components as independent variables.


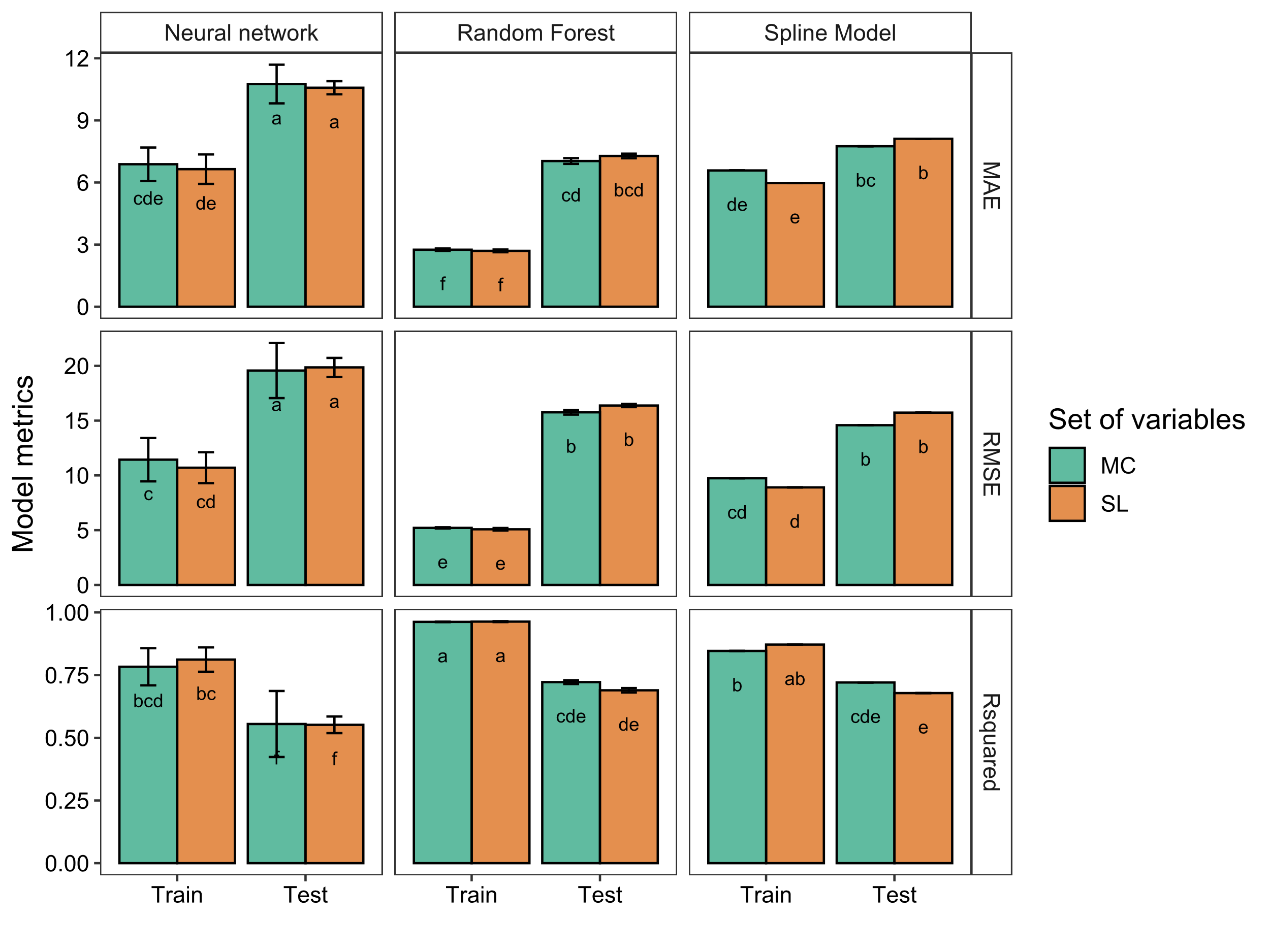


Figure 11 Bar chart containing the model metrics to predict the emulsion stability (mean absolute error (MAE), root mean square error (RMSE), and R^2^) generated five times for the neural network, random forest, and spline regression for yellow pea and lupine ingredients and mixtures of those with the main macro components (MC) and main macro components with a split according to soluble protein (SL) as independent variables. Letters indicate a significant different (P<0.05).

### All data combined with split

The random forest was also fitted with the main macro component but a with the protein and fibre split according to crop.

Table 5 Model metrics models for quantifying foaming capacity with main macro components as independent variables for ingredients of yellow pea, lupine and mixtures of those.

| Model | RMSE Train | R2 Train | MAE Train | RMSE Test | R2 Test | MAE Test |
| --- | --- | --- | --- | --- | --- | --- |
| Linear Model | 20.41 | 0.33 | 14.49 | 24.28 | 0.18 | 14.13 |
| Log Linear Model | 20.62 | 0.43 | 11.58 | 24.03 | 0.27 | 10.18 |
| Poly Model | 16.91 | 0.54 | 11.51 | 20.54 | 0.41 | 11.22 |
| Regularisation Model | 18.97 | 0.42 | 12.76 | 22.19 | 0.31 | 11.64 |
| Spline Model | 10.32 | 0.83 | 6.40 | 15.16 | 0.72 | 8.32 |
| Random Forest | 4.53 | 0.97 | 2.49 | 17.38 | 0.62 | 7.72 |
| Neural network | 9.87 | 0.84 | 5.77 | 13.78 | 0.81 | 8.78 |


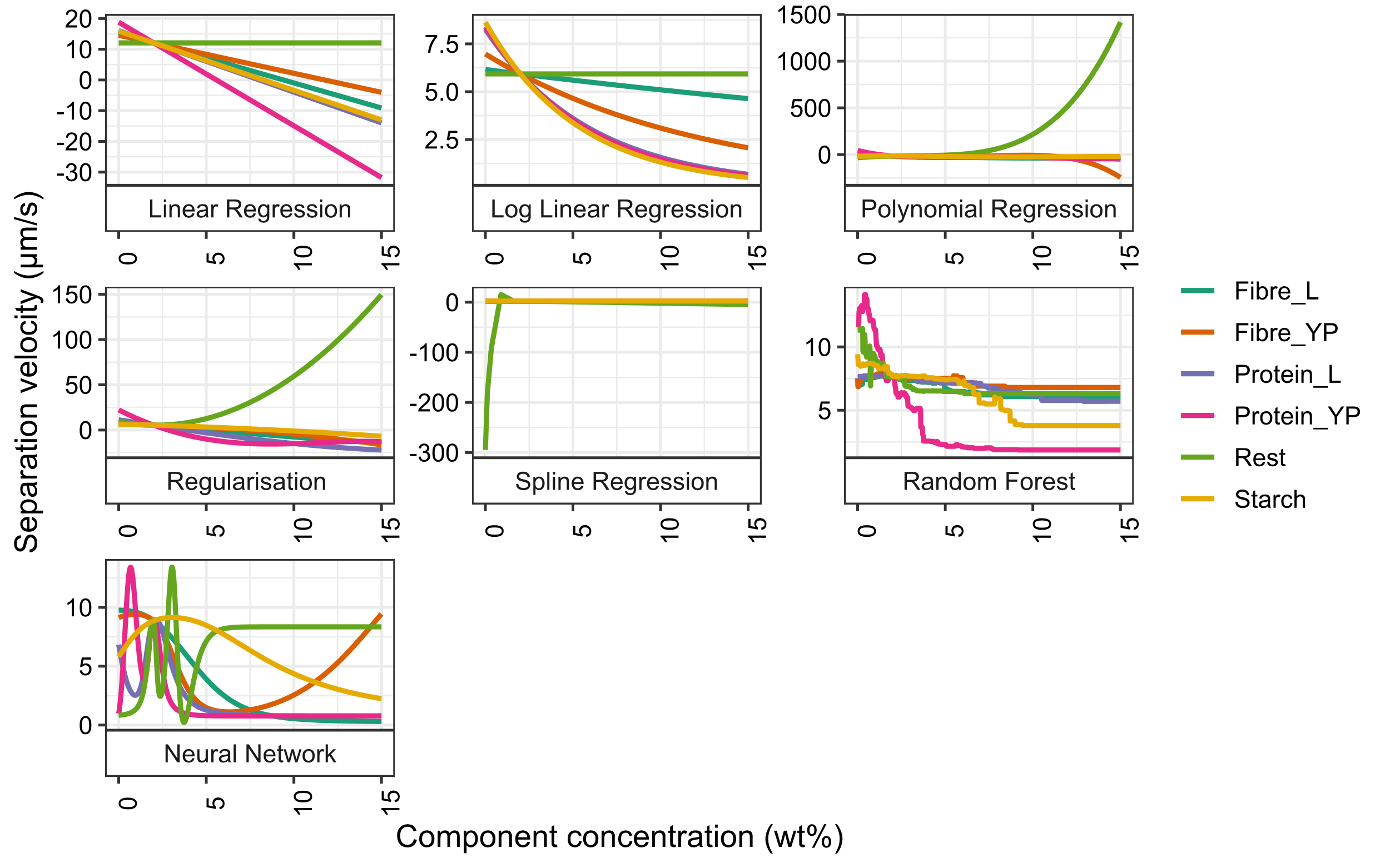


Figure 12 Scatterplot of the behaviour of each component in the evaluated models for quantifying the emulsion stability of yellow pea and lupine ingredients and mixtures of those with the main macro components as independent variables. Protein and fibre are split according to crop. The composition of each component increases from 1-15 wt% while the other stay constant at 2%.


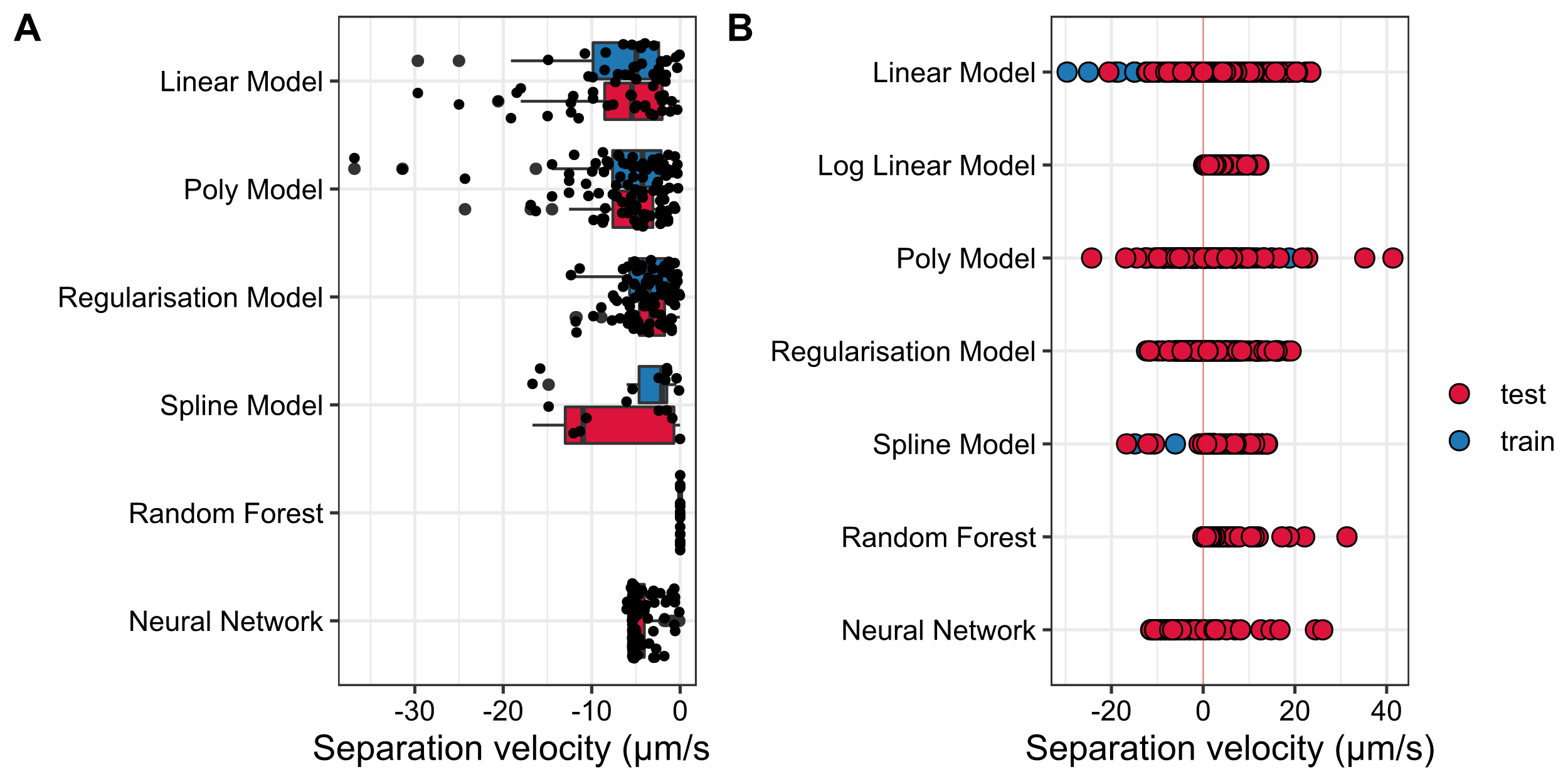


Figure 13 Box- and scatterplot wit negative values predicted by the evaluated models for quantifying the emulsions stability of yellow pea and lupine ingredients and mixtures of those with the main macro components as independent variables. Protein and fibre are split according to crop type.

## Foams

Table 6 Confusion matrices of liquid and semi solid classification used for the foams.

| Reference 🡺  Prediction 🡻 | Liquid | Semi solid | Liquid | Semi solid | Liquid | Semi solid |
| --- | --- | --- | --- | --- | --- | --- |
| **Train** | **Yellow Pea** |  | **Lupine** |  | **All** |  |
| Liquid | 177 | 7 | 52 | 1 | 251 | 8 |
| Semi solid | 0 | 23 | 0 | 9 | 5 | 35 |
| *Accuracy* | *0.97* |  | *0.98* |  | *0.96* |  |
| *Kappa* | *0.85* |  | *0.94* |  | *0.82* |  |
| **Test** | **Yellow Pea** |  | **Lupine** |  | **All** |  |
| Liquid | 52 | 4 | 28 | 2 | 96 | 3 |
| Semi solid | 0 | 4 | 0 | 7 | 4 | 21 |
| *Accuracy* | *0.93* |  | *0.95* |  | *0.94* |  |
| *Kappa* | *0.63* |  | *0.84* |  | *0.82* |  |
| Threshold  (mPa.s) | < 226 |  | < 412 |  | < 158 |  |

### Yellow pea

Model metrics indicate that the random forest is the most suitable model for predicting foaming capacity of yellow pea. The spline model also has acceptable metrics, but the trend of fibre goes negative. There is no significant difference when splitting the independent variables according to nativity or solubility with the random forest. The zero values are relatively close to zero, however, this could be improved. The model behaviour is acceptable; however, the irregularities are not ideal for the purpose of this study. Yet, a better model is not available.

Table 7 Model metrics models for quantifying foaming capacity with main macro components as independent variables for yellow pea ingredients.

| Model | RMSE Train | R2 Train | MAE Train | RMSE Test | R2 Test | MAE Test |
| --- | --- | --- | --- | --- | --- | --- |
| Linear Model | 43.82 | 0.48 | 30.12 | 39.11 | 0.63 | 26.39 |
| Log Linear Model | 53.39 | 0.37 | 36.91 | 70.37 | 0.41 | 42.17 |
| Poly Model | 41.04 | 0.55 | 29.38 | 39.96 | 0.62 | 28.98 |
| Regularisation Model | 41.12 | 0.55 | 29.17 | 39.41 | 0.63 | 27.70 |
| Spline Model | 35.07 | 0.67 | 26.12 | 36.93 | 0.67 | 26.52 |
| Random Forest | 15.96 | 0.94 | 11.70 | 38.29 | 0.65 | 26.84 |
| Neural network | 36.66 | 0.64 | 25.84 | 40.46 | 0.62 | 29.40 |


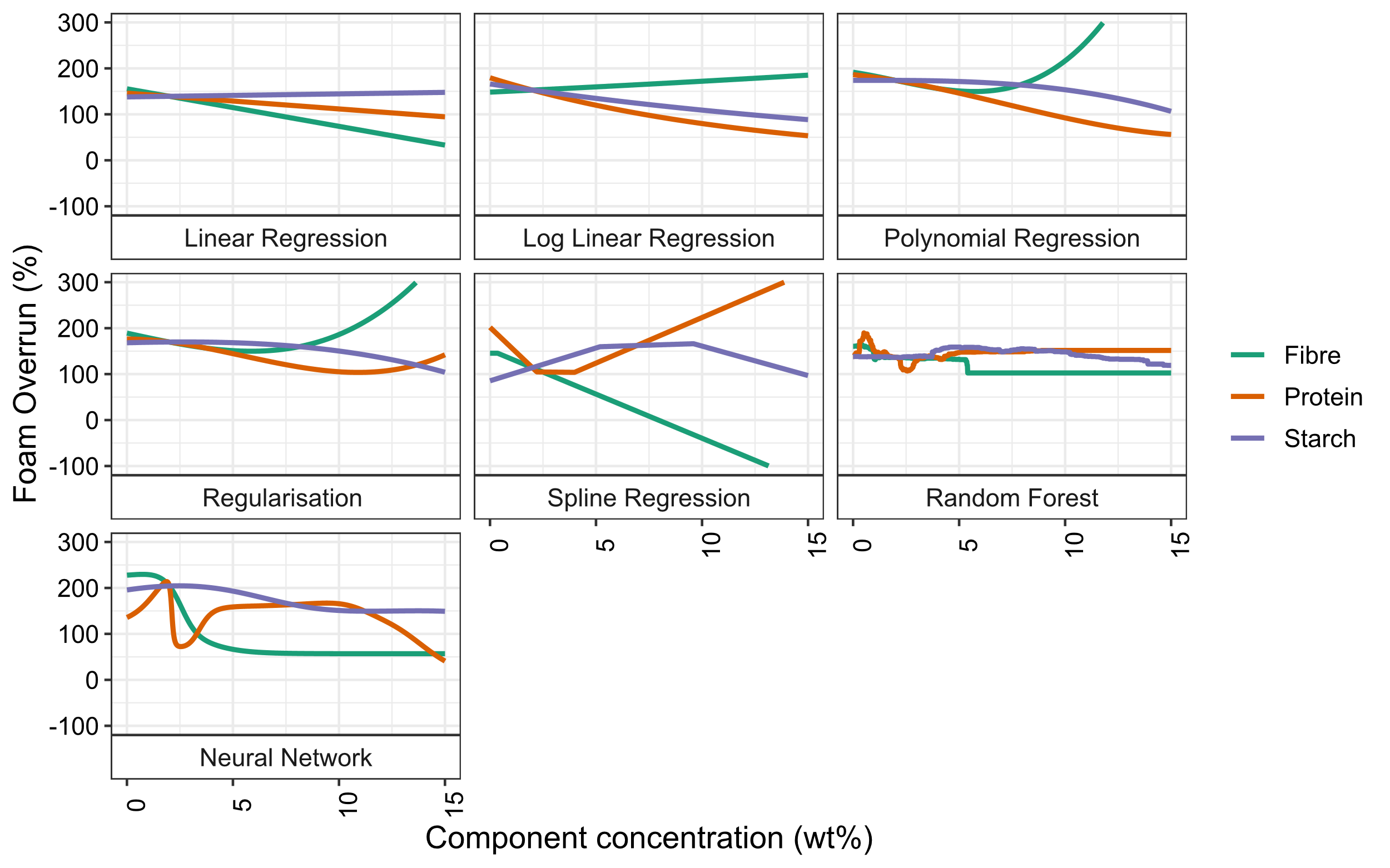


Figure 14 Scatterplot of the behaviour of each component in the evaluated models for quantifying the foaming capacity of yellow pea ingredients with the main macro components as independent variables. The composition of each component increases from 1-15 wt% while the other stay constant at 2%.


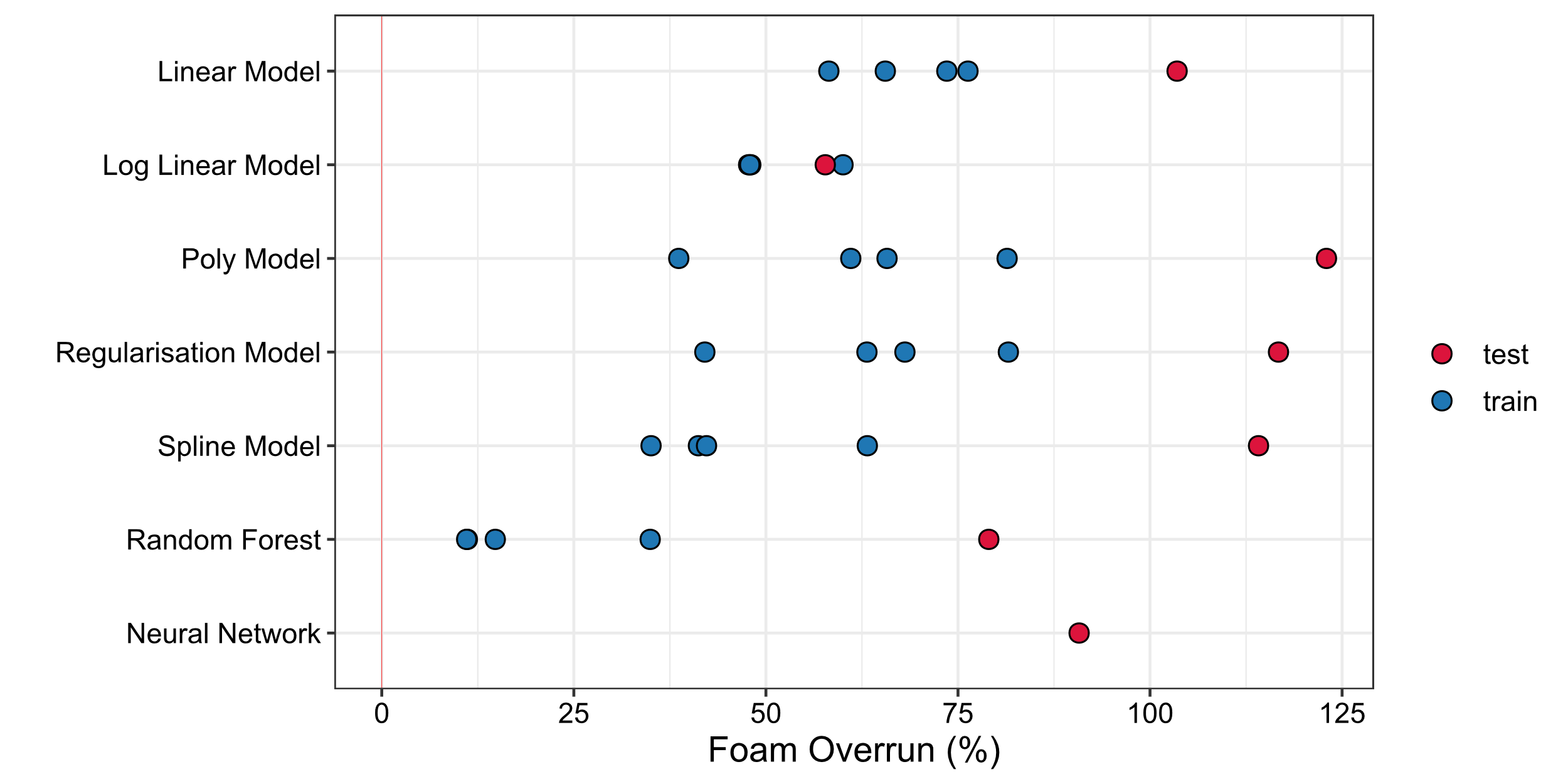


Figure 15 Scatterplot of predicted values for the datapoints of with an original value of zero, predicted by the evaluated models to for quantifying the foaming capacity of yellow pea ingredients with the main macro components as independent variables.


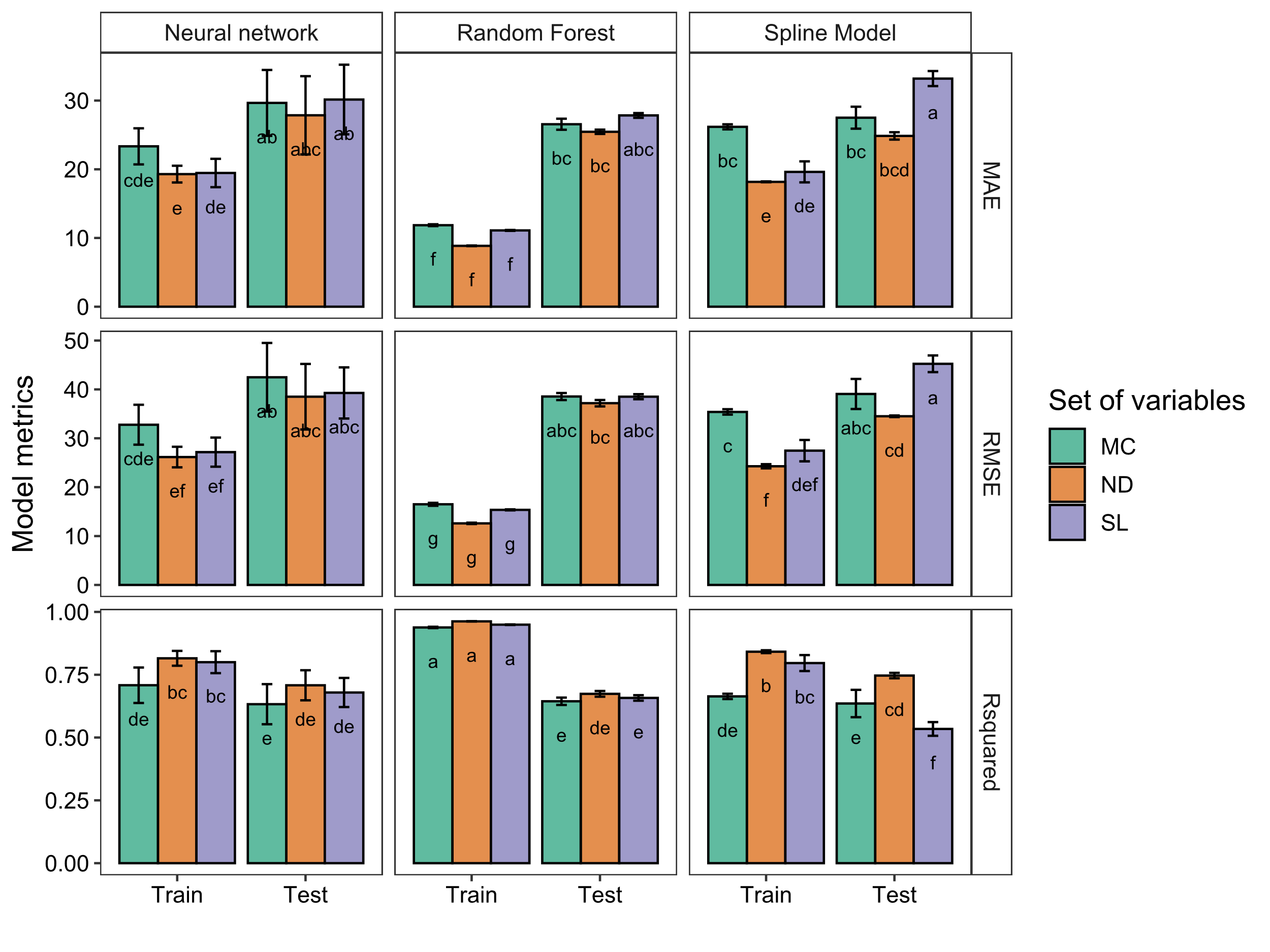


Figure 16 Bar chart containing the model metrics to predict the foaming capacity (mean absolute error (MAE), root mean square error (RMSE), and R^2^) generated five times for the neural network, random forest, and spline regression for yellow pea ingredients with the main macro components (MC) and main macro components with a split according to native (ND) and soluble protein (SL) as independent variables. Letters indicate a significant different (P<0.05).

### Lupine

Neural network and random forest are both potential models to quantify the foaming capacity of lupine ingredients based on all model metrics, behaviour, and negative values. Also, the predictions seem physically feasible since they are not negative for these model types. The prediction of the behaviour of each components shows that the foaming capacity predicted by the neural network remains at one percentage of overrun, also this model suffers from more overfitting (train error << test error) compared to the random forest. No significant differences were found between the sets of variables. Therefore, the random forest is selected with the main macro components as predictive variables.

Table 8 Model metrics models for quantifying foaming capacity with main macro components as independent variables for lupine ingredients.

| Model | RMSE Train | R2 Train | MAE Train | RMSE Test | R2 Test | MAE Test |
| --- | --- | --- | --- | --- | --- | --- |
| Linear Model | 63.01 | 0.28 | 48.20 | 75.09 | 0.27 | 66.87 |
| Log Linear Model | 65.06 | 0.27 | 42.43 | 69.51 | 0.27 | 52.45 |
| Poly Model | 41.41 | 0.69 | 33.07 | 67.98 | 0.47 | 57.16 |
| Regularisation Model | 58.57 | 0.41 | 43.51 | 68.26 | 0.29 | 59.85 |
| Spline Model | 28.80 | 0.85 | 23.12 | 54.40 | 0.63 | 45.31 |
| Random Forest | 27.48 | 0.90 | 17.95 | 71.83 | 0.18 | 56.77 |
| Neural network | 9.98 | 0.98 | 7.35 | 71.81 | 0.53 | 52.71 |


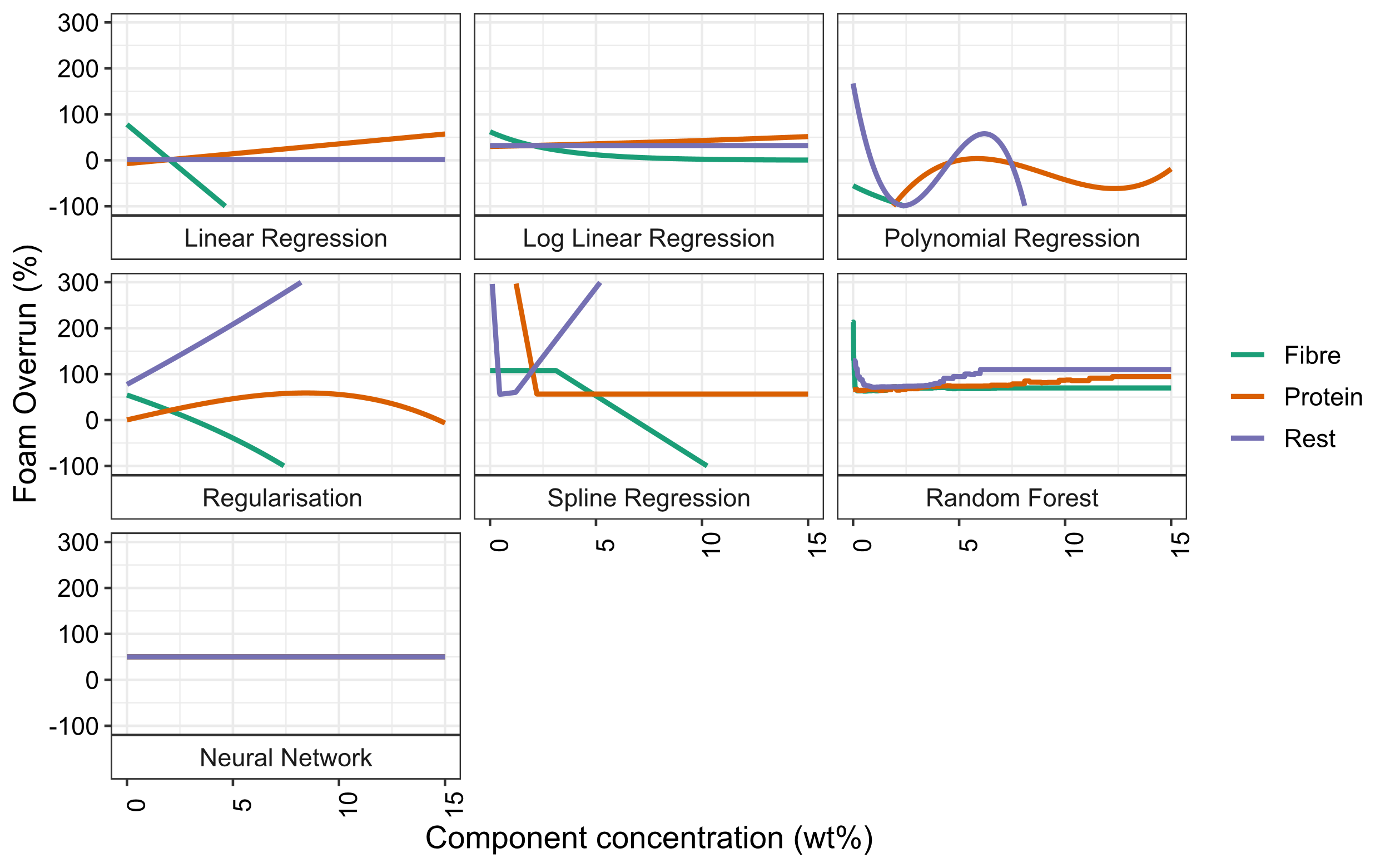


Figure 17 Scatterplot of the behaviour of each component in the evaluated models for quantifying the foam capacity of lupine ingredients with the main macro components as independent variables. The composition of each component increases from 1-15 wt% while the other stay constant at 2%.


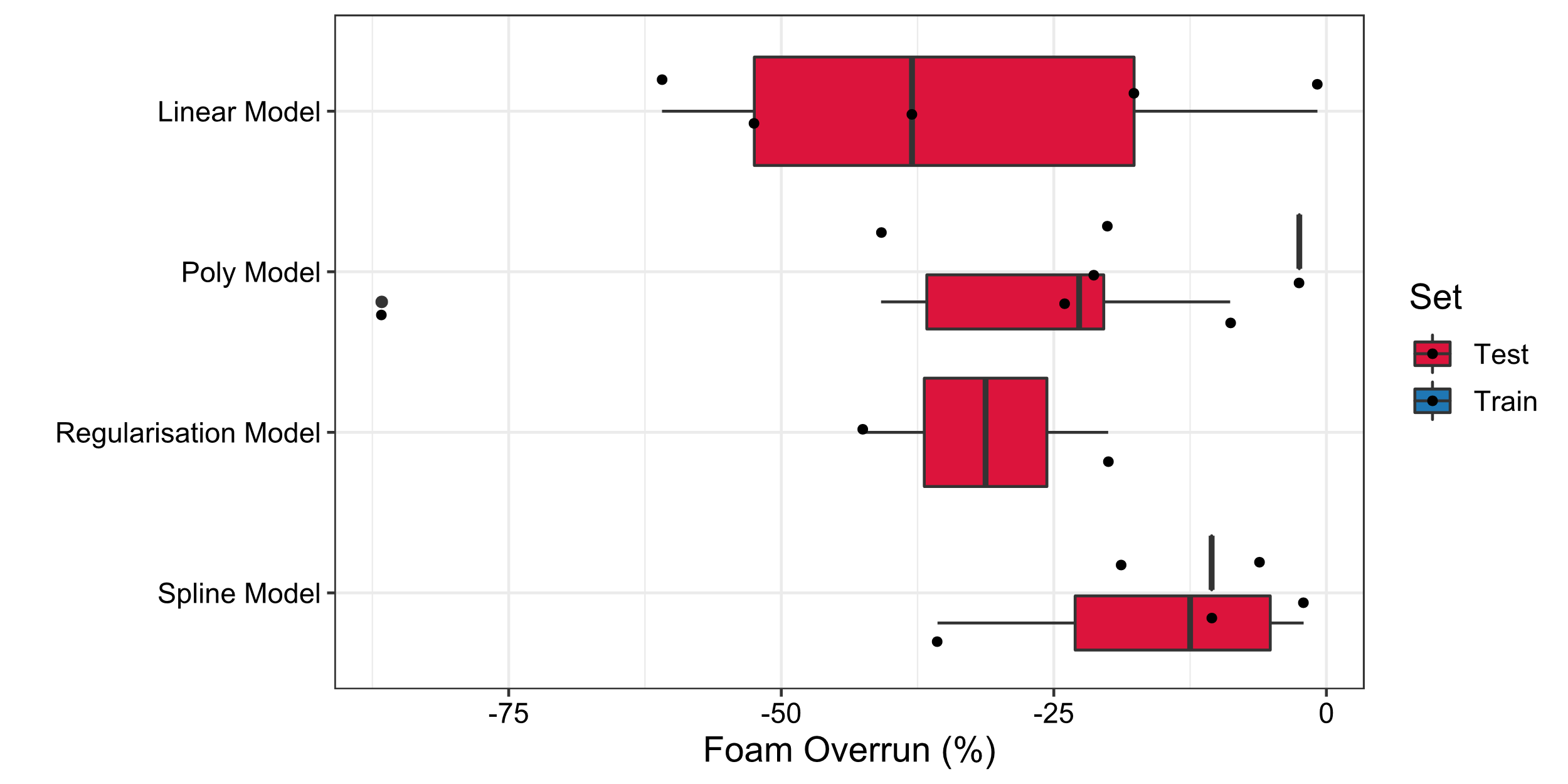


Figure 18 Boxplot of negative values predicted by the evaluated models to for quantifying the foaming capacity of lupine ingredients with the main macro components as independent variables.


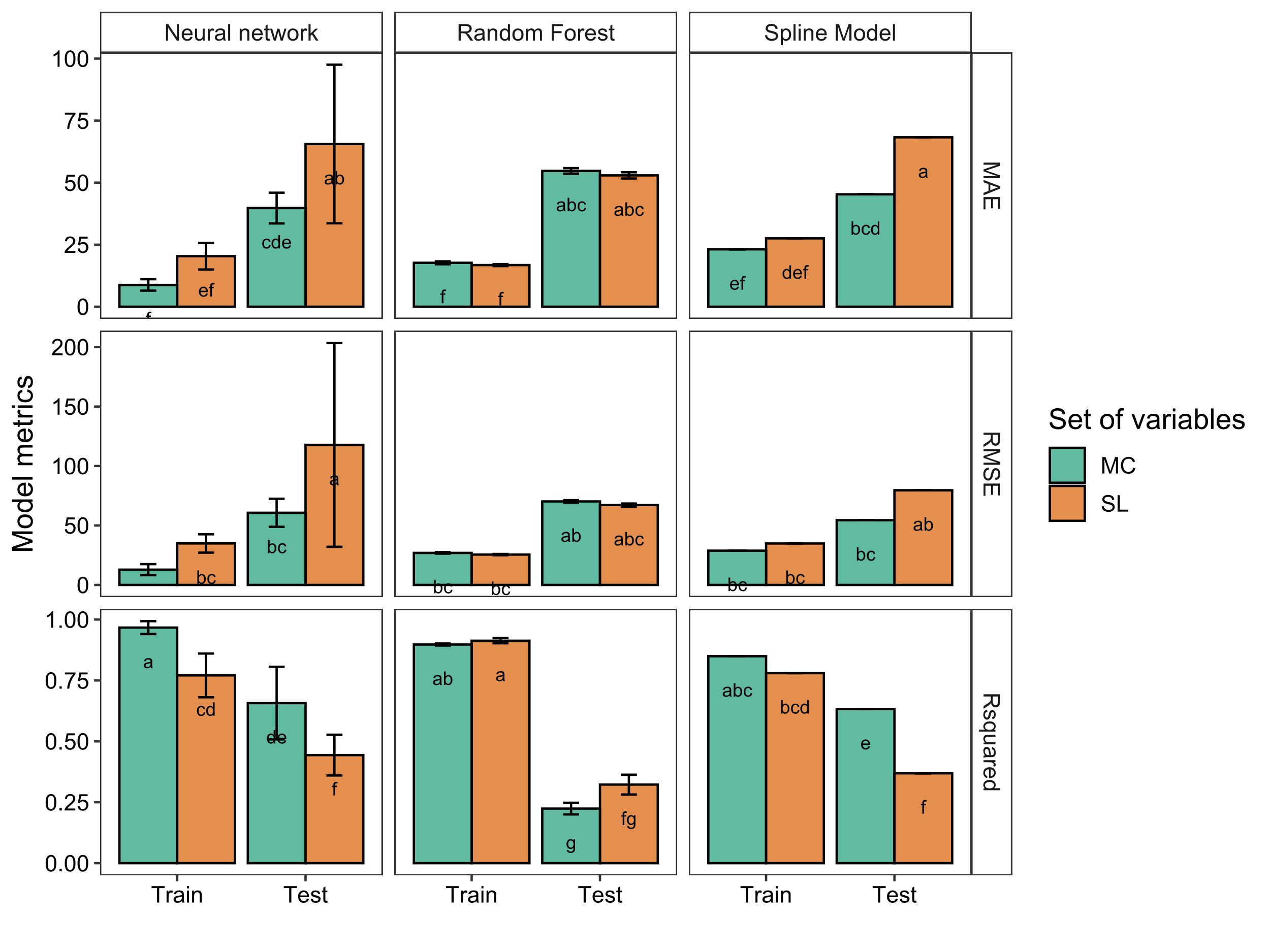


Figure 19 Bar chart containing the model metrics to predict the foaming capacity (mean absolute error (MAE), root mean square error (RMSE), and R^2^) generated five times for the neural network, random forest, and spline regression for lupine ingredients with the main macro components (MC) and main macro components with a split according to soluble protein (SL) as independent variables. Letters indicate a significant different (P<0.05).

### All data combined

The random forest can quantify the foaming capacity of all data combined (yellow pea and lupine ingredients and combinations of those) with the most favourable model metrics and behaviour. Notably, the random forest does show some artefacts as it is a discontinues trend, however, by a lack of better models the random forest is still considered.

Table 9 Model metrics models for quantifying foaming capacity with main macro components as independent variables for yellow pea, lupine and mixtures of those.

| Model | RMSE Train | R2 Train | MAE Train | RMSE Test | R2 Test | MAE Test |
| --- | --- | --- | --- | --- | --- | --- |
| Linear Model | 57.45 | 0.29 | 41.06 | 65.01 | 0.17 | 46.83 |
| Log Linear Model | 63.30 | 0.23 | 43.13 | 78.90 | 0.10 | 52.02 |
| Poly Model | 51.33 | 0.43 | 36.84 | 60.13 | 0.30 | 46.11 |
| Regularisation Model | 51.57 | 0.43 | 36.96 | 59.38 | 0.31 | 45.88 |
| Spline Model | 37.75 | 0.69 | 28.10 | 48.03 | 0.53 | 35.34 |
| Random Forest | 19.84 | 0.94 | 14.03 | 50.30 | 0.49 | 37.32 |
| Neural network | 49.22 | 0.48 | 35.12 | 64.18 | 0.25 | 48.00 |


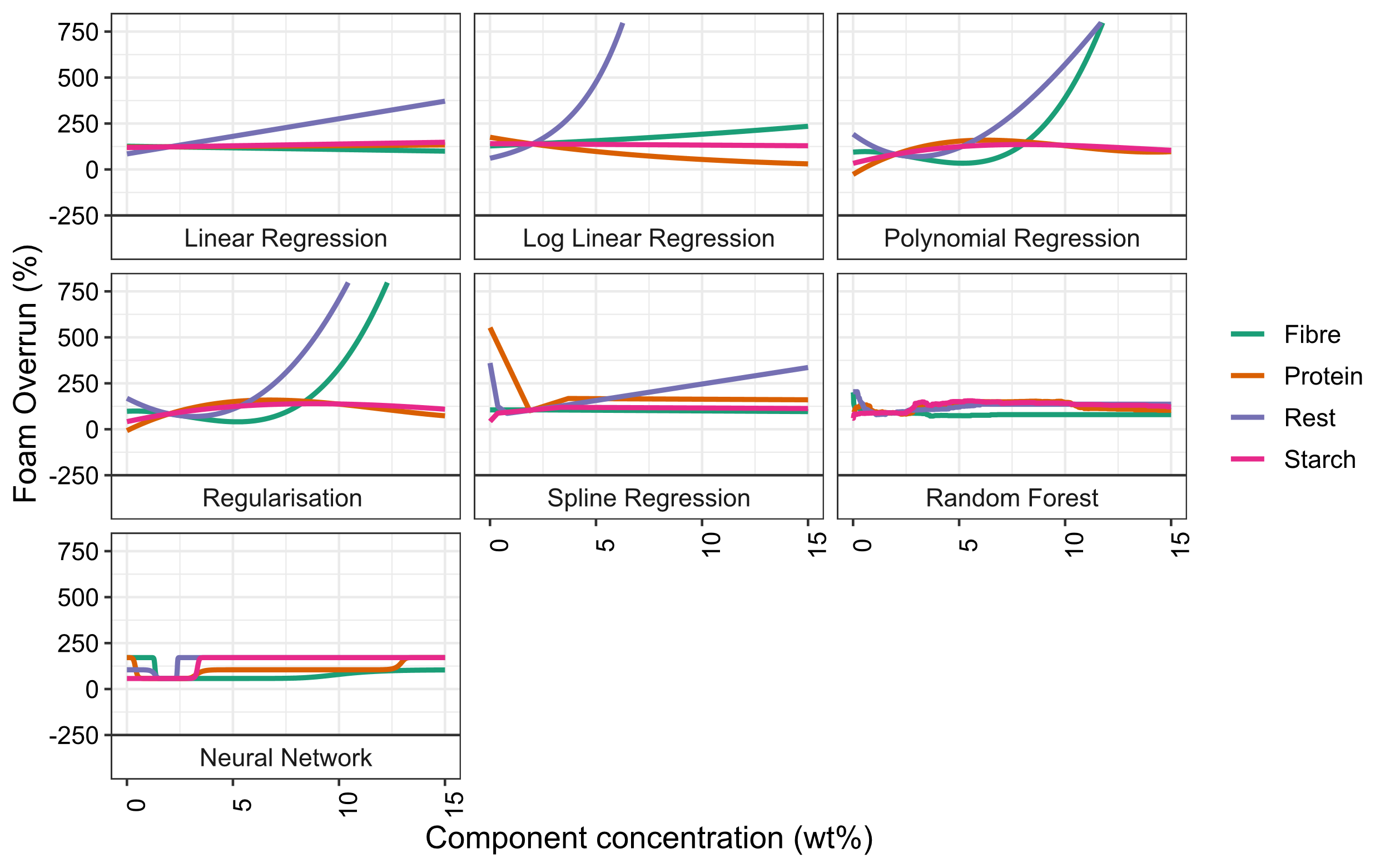


Figure 20 Scatterplot of the behaviour of each component in the evaluated models for quantifying the foaming capacity of yellow pea and lupine ingredients and mixtures of those with the main macro components as independent variables. The composition of each component increases from 1-15 wt% while the other stay constant at 2%.


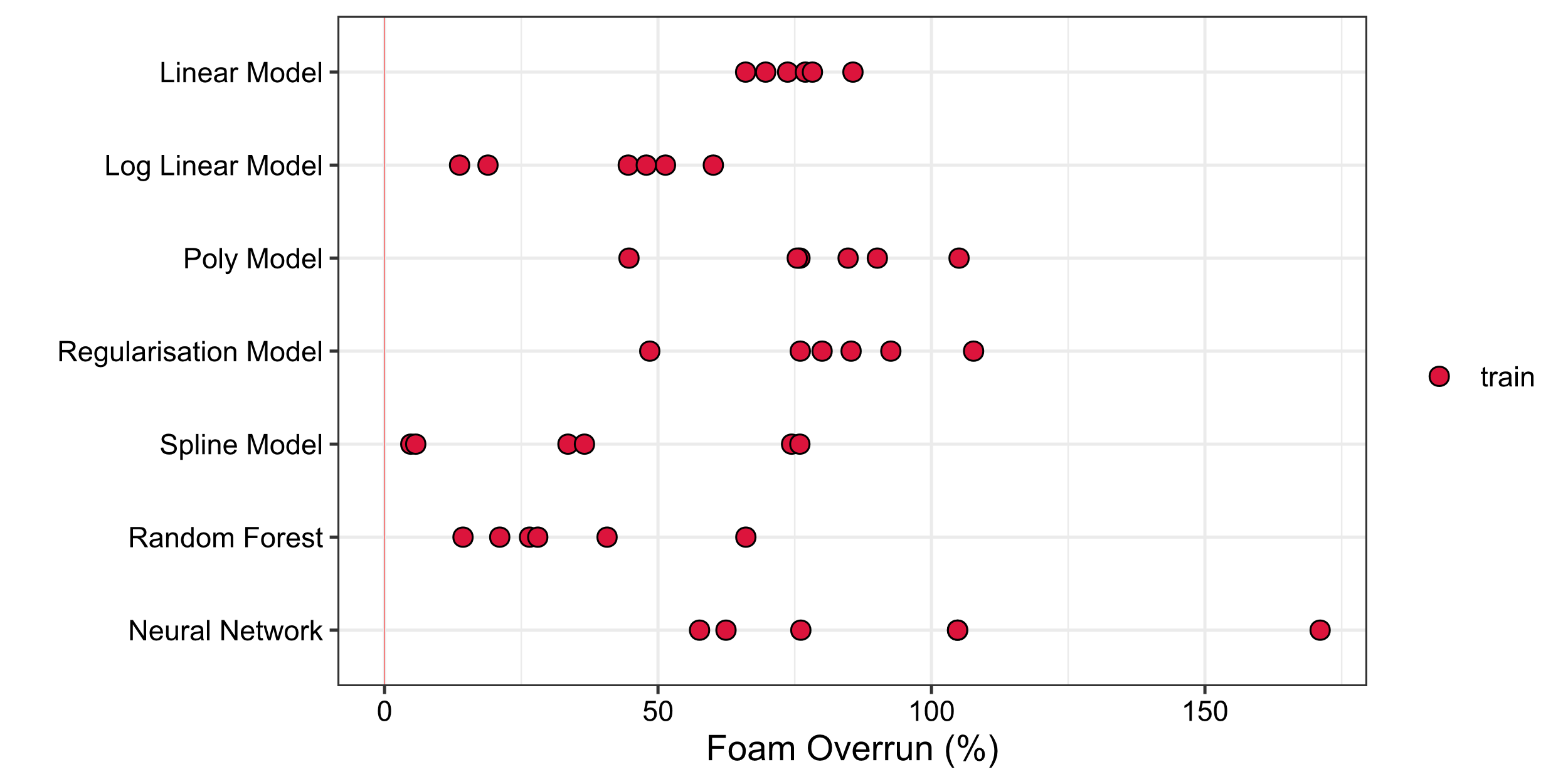


Figure 21 Scatterplot with predicted values for the datapoints of with an original value of zero, as predicted by the evaluated models to for quantifying the foaming capacity of yellow pea and lupine ingredients and mixtures of those with the main macro components as independent variables.

### All data combined with a split

Table 10 Model metrics models for quantifying foaming capacity with main macro components as independent variables for yellow pea, lupine ingredients and mixtures of those with a split in protein and fibre according to crop.

| Model | RMSE Train | R2 Train | MAE Train | RMSE Test | R2 Test | MAE Test |
| --- | --- | --- | --- | --- | --- | --- |
| Linear Model | 58.41 | 0.25 | 41.58 | 66.04 | 0.13 | 50.38 |
| Log Linear Model | 64.42 | 0.18 | 42.16 | 69.09 | 0.17 | 48.03 |
| Poly Model | 51.55 | 0.42 | 37.06 | 56.97 | 0.36 | 41.52 |
| Regularisation Model | 51.82 | 0.41 | 37.12 | 57.42 | 0.35 | 42.39 |
| Spline Model | 38.83 | 0.67 | 28.08 | 59.82 | 0.33 | 43.07 |
| Random Forest | 20.16 | 0.94 | 14.02 | 47.66 | 0.55 | 35.20 |
| Neural network | 38.11 | 0.68 | 28.70 | 81.60 | 0.34 | 57.49 |


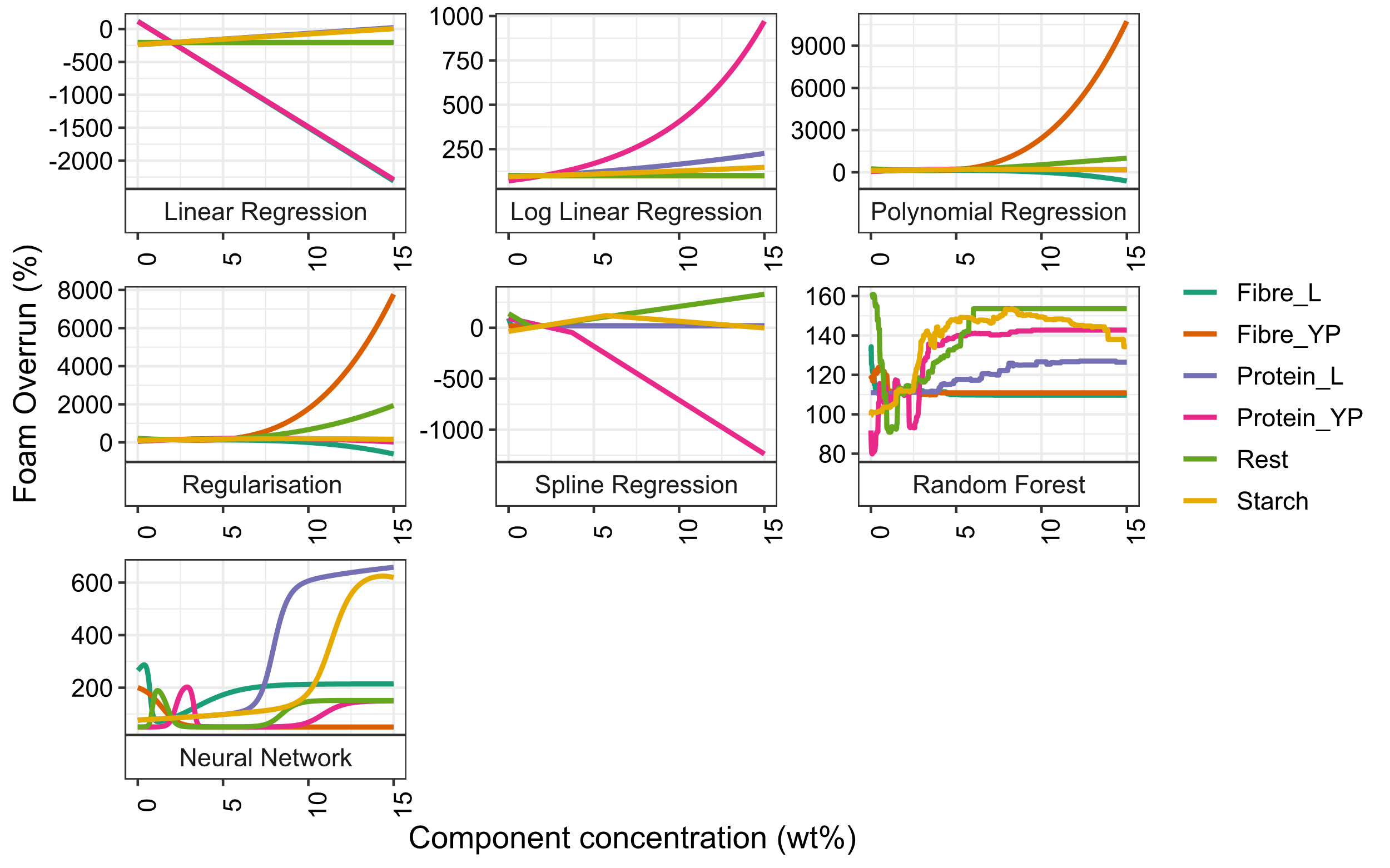


Figure 22 Scatterplot of the behaviour of each component in the evaluated models for quantifying the foaming capacity of yellow pea and lupine ingredients and mixtures of those with the main macro components as independent variables. Protein and fibre are split according to crop. The composition of each component increases from 1-15 wt% while the other stay constant at 2%.


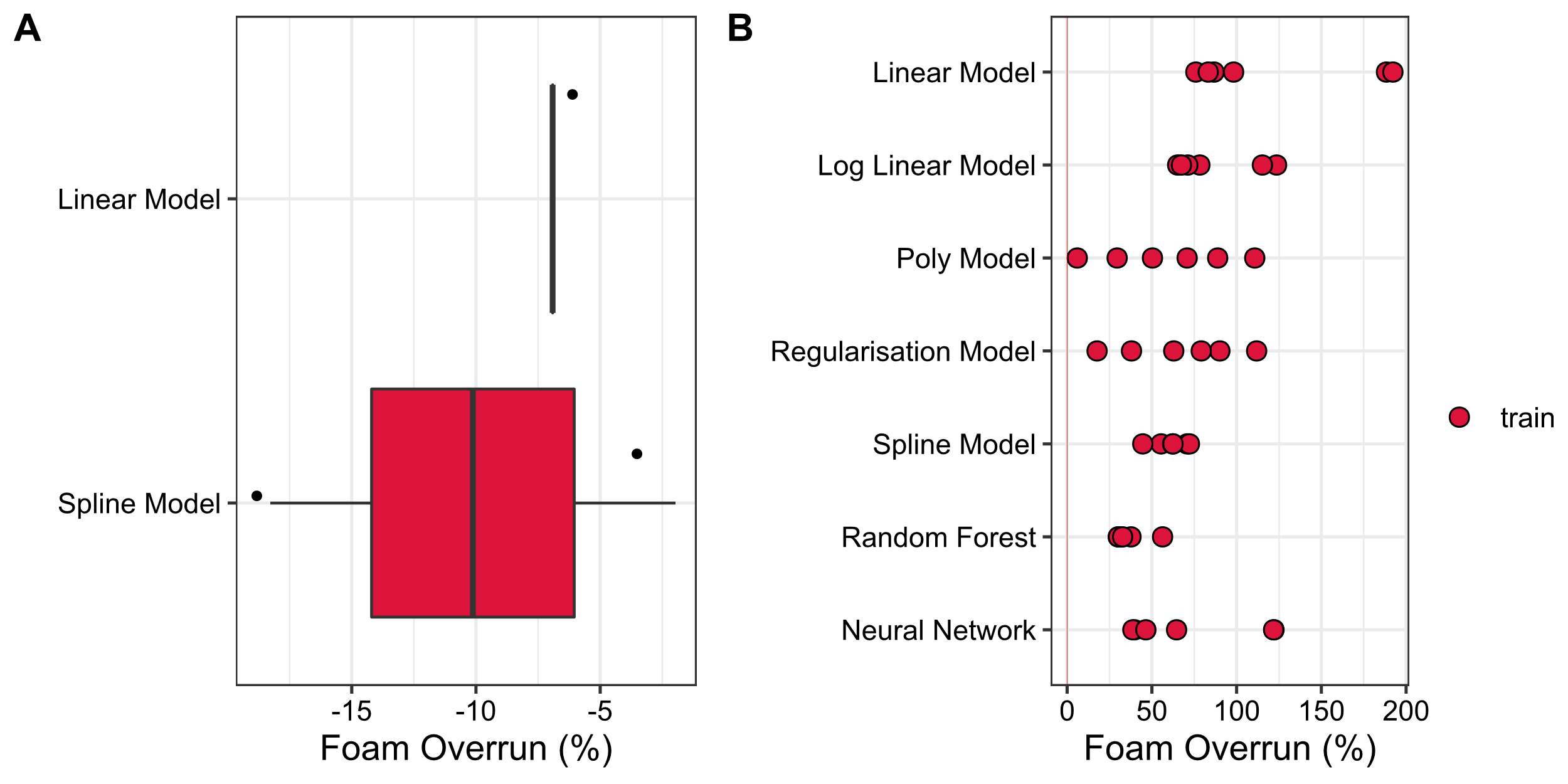


Figure 23 Box- and scatterplot wit negative values (A) and predicted values for the datapoints of with an original value of zero (B) predicted by the evaluated models to for quantifying the foaming capacity of yellow pea and lupine ingredients and mixtures of those with the main macro components as independent variables. Protein and fibre are split according to crop type.

## Gelation

### Yellow pea

The best model to quantify the gel stiffness of yellow pea ingredients is a neural network since it has the best test errors with the most physically plausible behaviour. Just the test MAE and RMSE are slightly lower with a split according to processing history, however, the other are not significant different. Therefore, the variables with the main macro components are considered. The metrics from the repeated neural network show some variation, therefore the model with the lowest errors is considered in further analyses.

Table 11 Model metrics models for quantifying gel stiffness with main macro components as independent variables for yellow pea ingredients.

| Model | RMSE Train | R2 Train | MAE Train | RMSE Test | R2 Test | MAE Test |
| --- | --- | --- | --- | --- | --- | --- |
| Linear Model | 49.51 | 0.93 | 38.95 | 71.30 | 0.83 | 48.46 |
| Log Linear Model | 216.89 | 0.77 | 82.55 | 169.81 | 0.73 | 86.16 |
| Poly Model | 48.30 | 0.93 | 36.94 | 47.43 | 0.92 | 35.14 |
| Regularisation Model | 49.48 | 0.93 | 37.47 | 49.22 | 0.91 | 34.89 |
| Spline Model | 29.97 | 0.97 | 20.93 | 74.87 | 0.82 | 46.52 |
| Random Forest | 25.09 | 0.98 | 16.65 | 85.63 | 0.74 | 55.46 |
| Neural network | 31.82 | 0.97 | 22.57 | 64.60 | 0.88 | 42.05 |


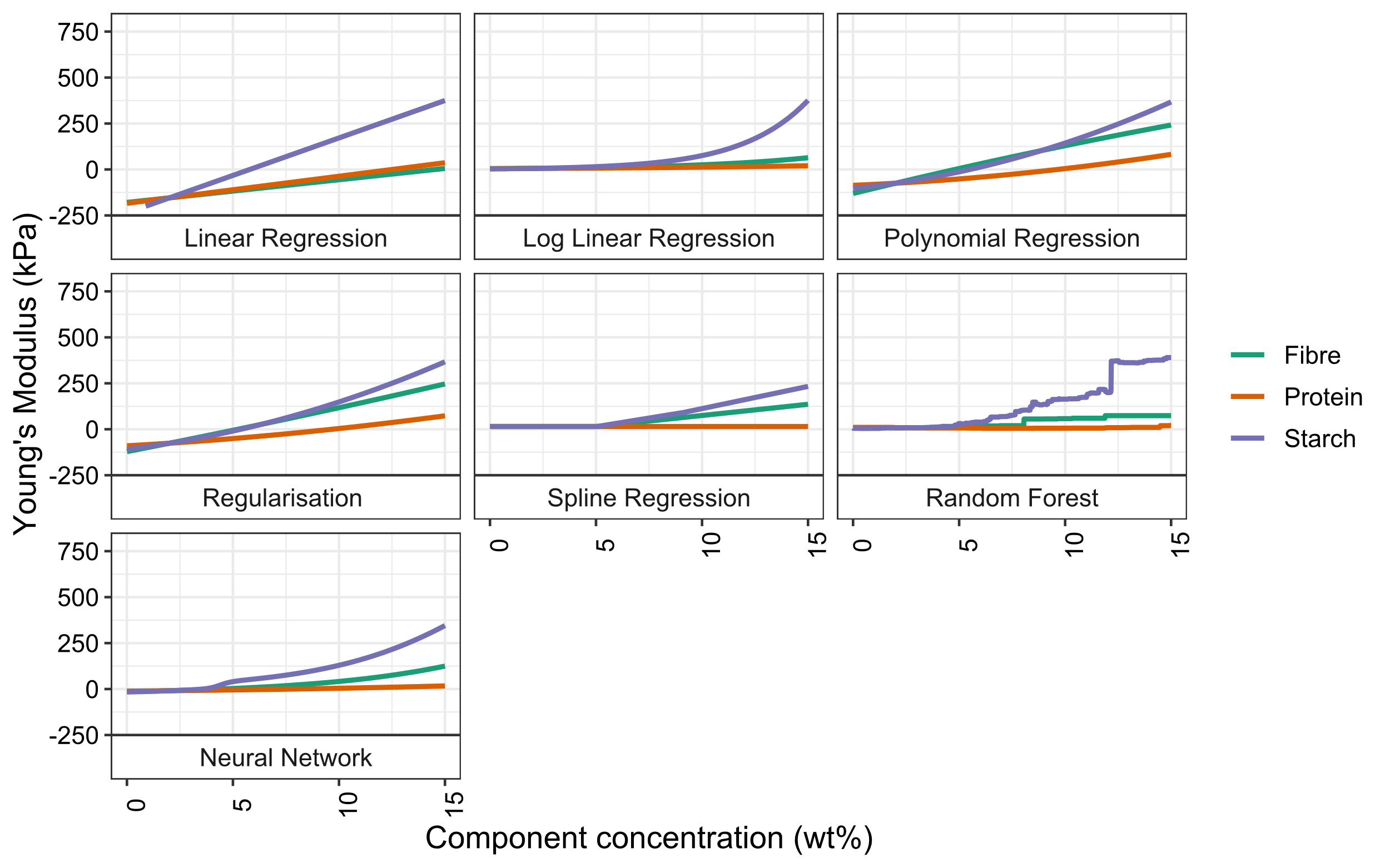


Figure 24 Scatterplot of the behaviour of each component in the evaluated models for quantifying the gel stiffness of yellow pea ingredients with the main macro components as independent variables. The composition of each component increases from 1-15 wt% while the other stay constant at 2%.


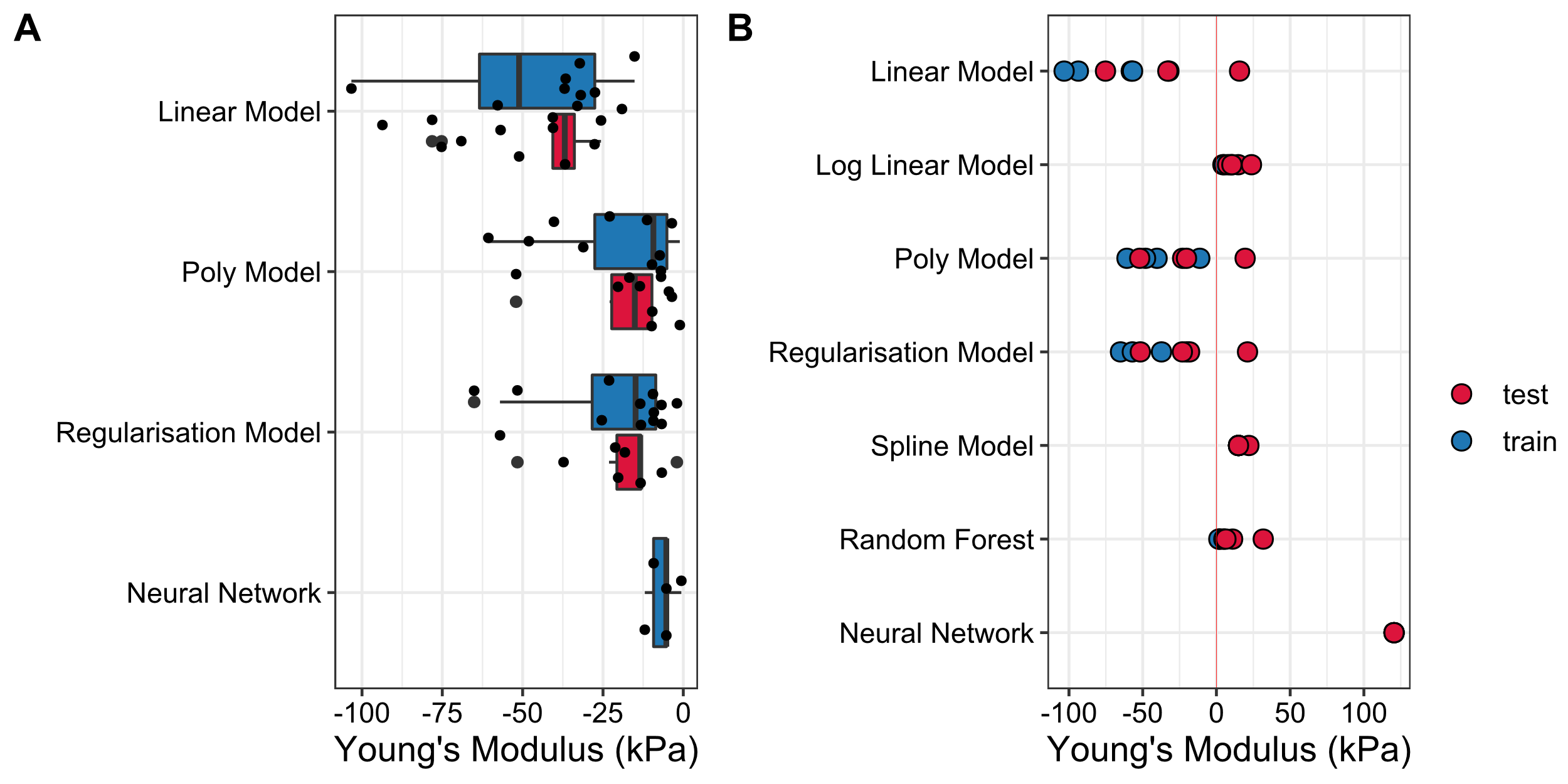


Figure 25 Scatter- and boxplot of negative values (A) and predicted values for the datapoints of with an original value of zero (B) predicted by the evaluated models to for quantifying the gel stiffness of yellow pea with the main macro components as independent variables.


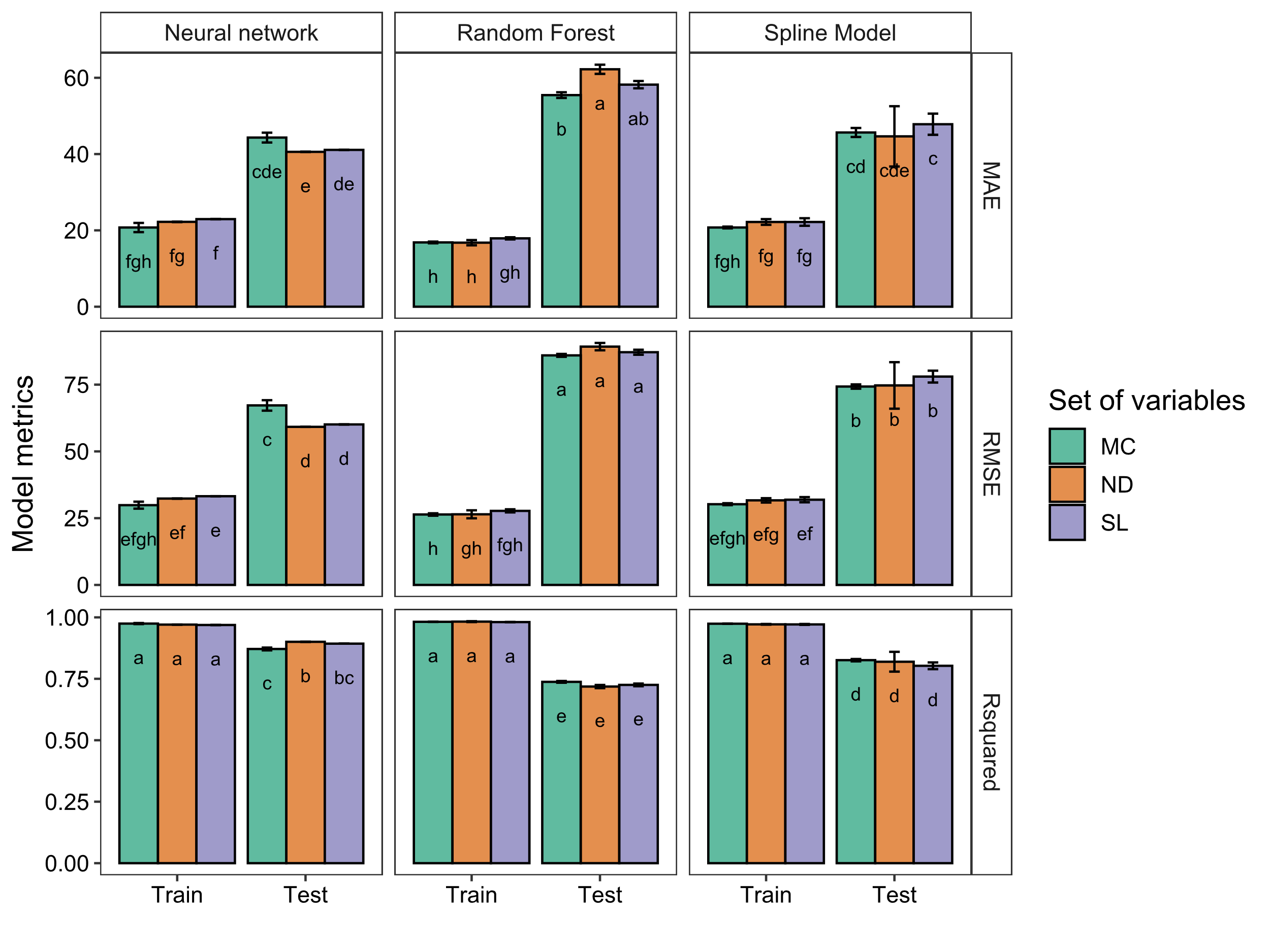


Figure 26 Bar chart containing the model metrics to predict the gel stiffness (mean absolute error (MAE), root mean square error (RMSE), and R^2^) generated five times for the neural network, random forest, and spline regression for yellow pea ingredients with the main macro components (MC) and main macro components with a split according to native (ND) and soluble protein (SL) as independent variables. Letters indicate a significant difference (P<0.05).

### Lupine

Based on model metrics the best model to quantify the gel stiffness of lupine ingredients is a neural network. Regularised polynomial linear regression also has promising metrics but predicts more negative values in trend behaviour and negative value plot. No significant differences between sets of variables and therefore the main macro components without split are used. The metrics from the repeated neural network show some variation, therefore the model with the lowest errors is considered in further analyses.

Table 12 Model metrics models for quantifying gel stiffness with main macro components as independent variables for lupine ingredients.

| Model | RMSE Train | R2 Train | MAE Train | RMSE Test | R2 Test | MAE Test |
| --- | --- | --- | --- | --- | --- | --- |
| Linear Model | 9.24 | 0.97 | 7.22 | 14.85 | 0.91 | 10.94 |
| Log Linear Model | 28.32 | 0.93 | 11.20 | 24.46 | 0.73 | 11.38 |
| Poly Model | 18.26 | 0.89 | 13.88 | 18.80 | 0.84 | 14.96 |
| Regularisation Model | 20.11 | 0.87 | 13.72 | 16.27 | 0.89 | 12.88 |
| Spline Model | 9.64 | 0.97 | 6.42 | 19.00 | 0.90 | 11.42 |
| Random Forest | 20.91 | 0.88 | 9.91 | 22.58 | 0.77 | 12.28 |
| Neural network | 5.54 | 0.99 | 3.27 | 15.14 | 0.92 | 7.52 |


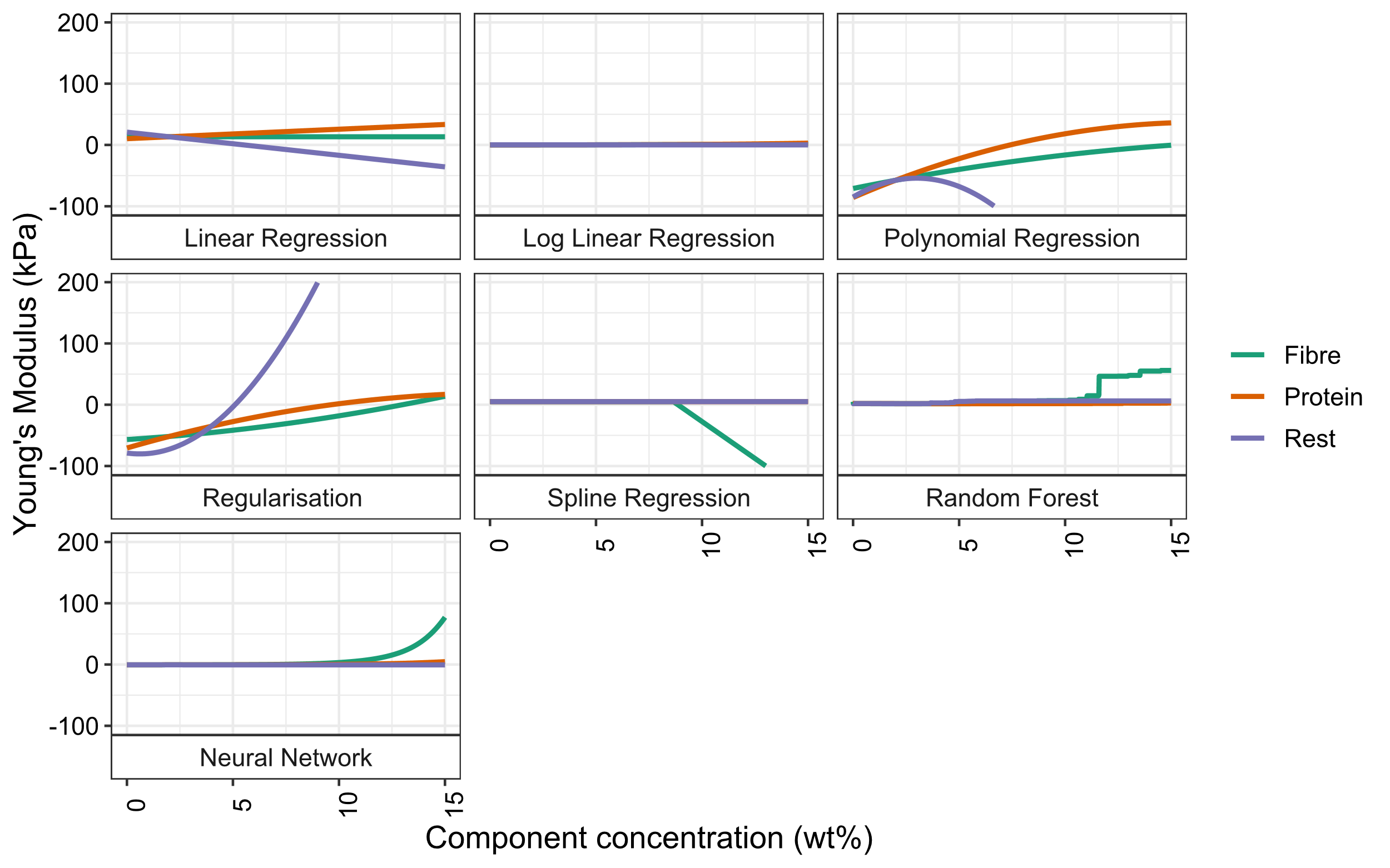


Figure 27 Scatterplot of the behaviour of each component in the evaluated models for quantifying the gel stiffness of lupine ingredients with the main macro components as independent variables. The composition of each component increases from 1-15 wt% while the other stay constant at 2%.


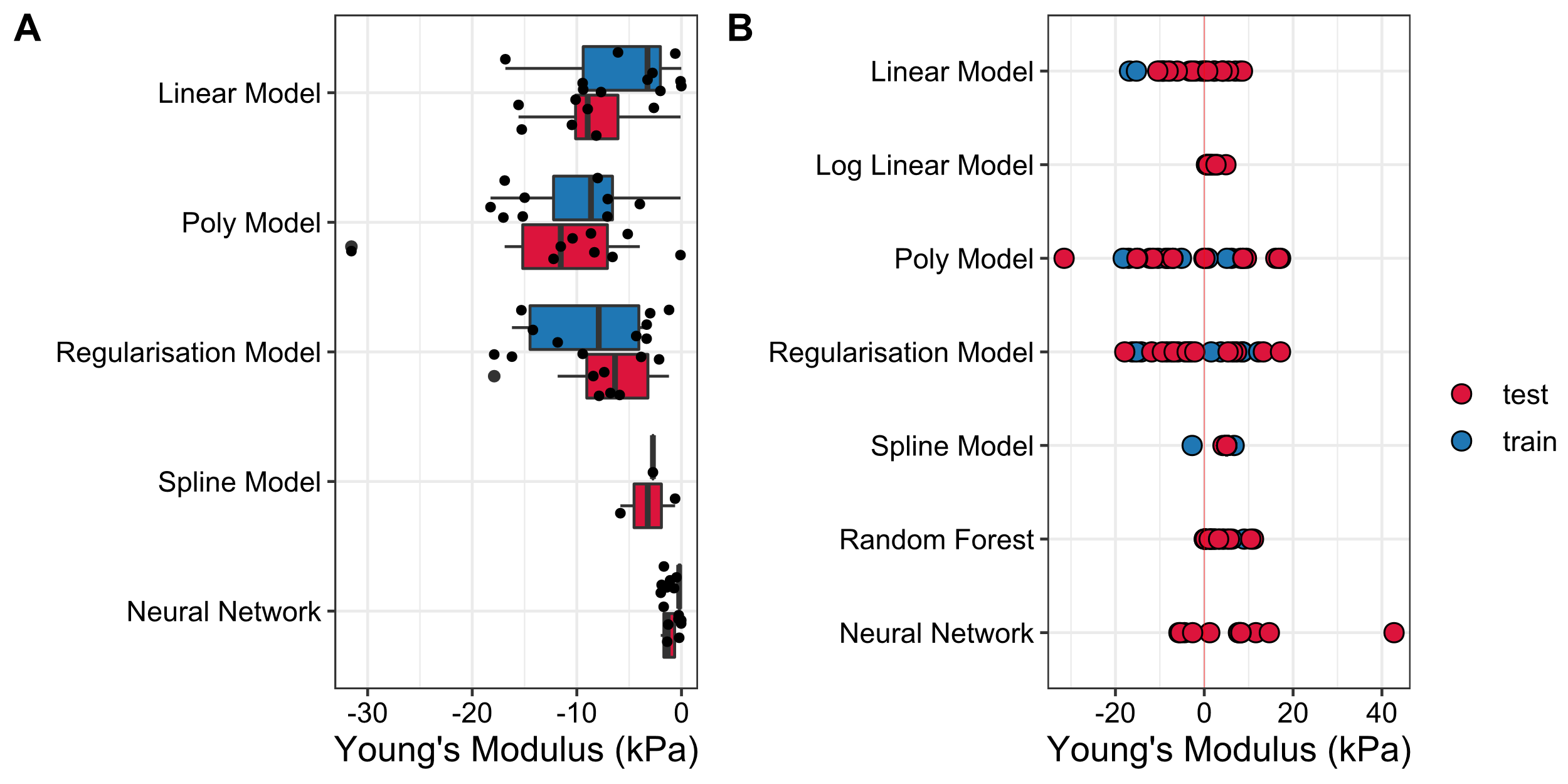


Figure 28 Scatter and boxplot of negative values (A) and predicted values for the datapoints of with an original value of zero (B) predicted by the evaluated models to for quantifying the gel stiffness of lupine ingredients with the main macro components as independent variables.


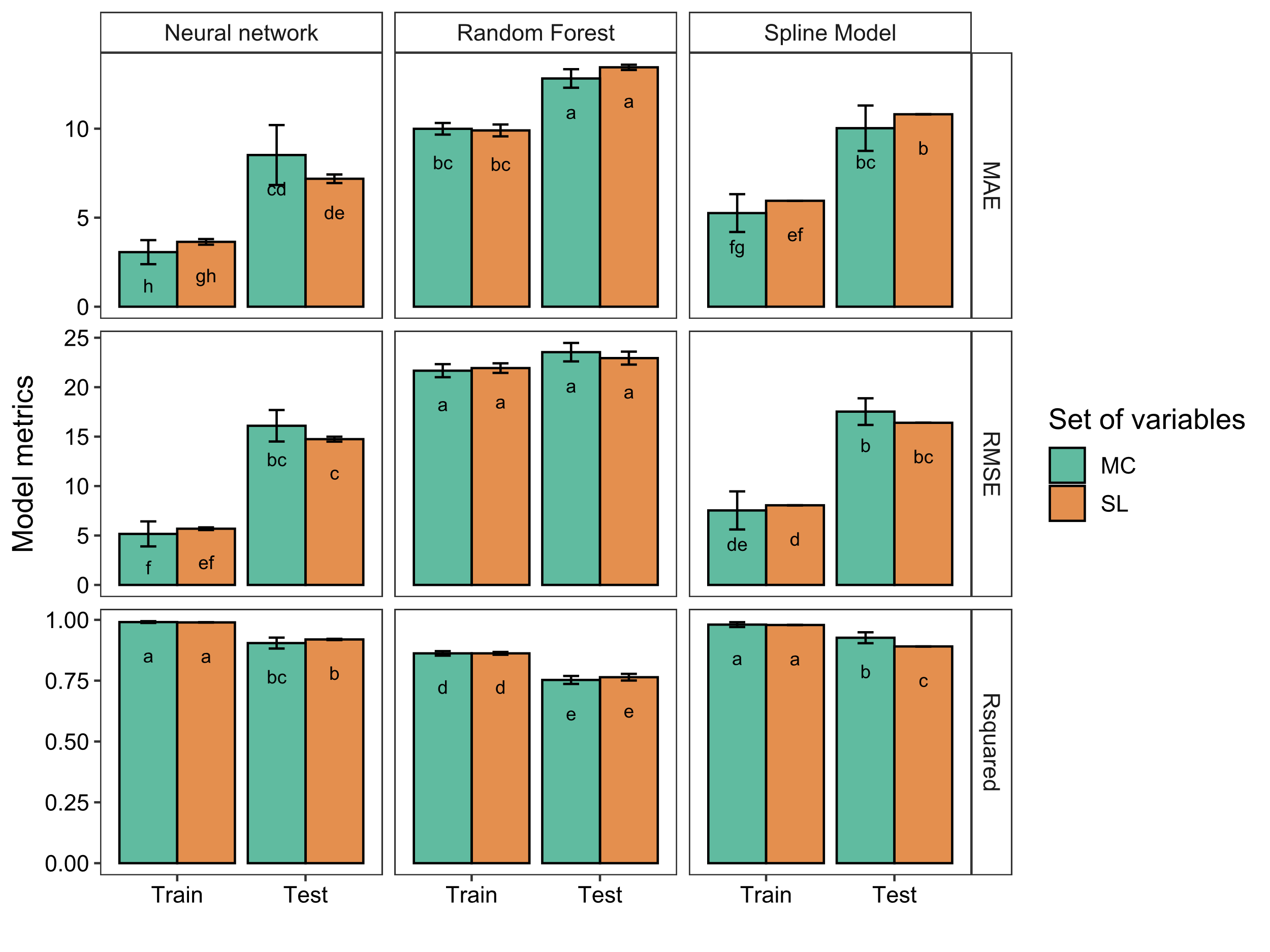


Figure 29 Bar chart containing the model metrics to predict the gel stiffness (mean absolute error (MAE), root mean square error (RMSE), and R^2^) generated five times for the neural network, random forest, and spline regression for yellow pea ingredients with the main macro components (MC) and main macro components with a split according to soluble protein (SL) as independent variables. Letters indicate a significant different (P<0.05).

### All data combined

The spline regression, polynomial model, and regularisation polynomial linear regression, and neural network are of interest. The latter produces the least number of negative sample and has feasible behaviour and is therefore considered the best. Since no effect of splitting protein on the model metrics for the individual models to predict the gel stiffness, these are also not considered here.

Table 13 Model metrics models for quantifying gel stiffness with main macro components as independent variables for yellow pea, lupine and mixtures of those.

| Model | RMSE Train | R2 Train | MAE Train | RMSE Test | R2 Test | MAE Test |
| --- | --- | --- | --- | --- | --- | --- |
| Linear Model | 54.73 | 0.90 | 44.64 | 66.24 | 0.83 | 47.15 |
| Log Linear Model | 475.31 | 0.61 | 132.01 | 288.54 | 0.65 | 108.94 |
| Poly Model | 52.44 | 0.91 | 40.39 | 51.66 | 0.89 | 38.59 |
| Regularisation Model | 53.12 | 0.91 | 39.34 | 53.75 | 0.88 | 39.68 |
| Spline Model | 30.10 | 0.97 | 21.01 | 58.03 | 0.87 | 33.59 |
| Random Forest | 24.34 | 0.98 | 14.45 | 74.55 | 0.78 | 44.52 |
| Neural network | 36.88 | 0.96 | 26.70 | 54.87 | 0.89 | 33.63 |


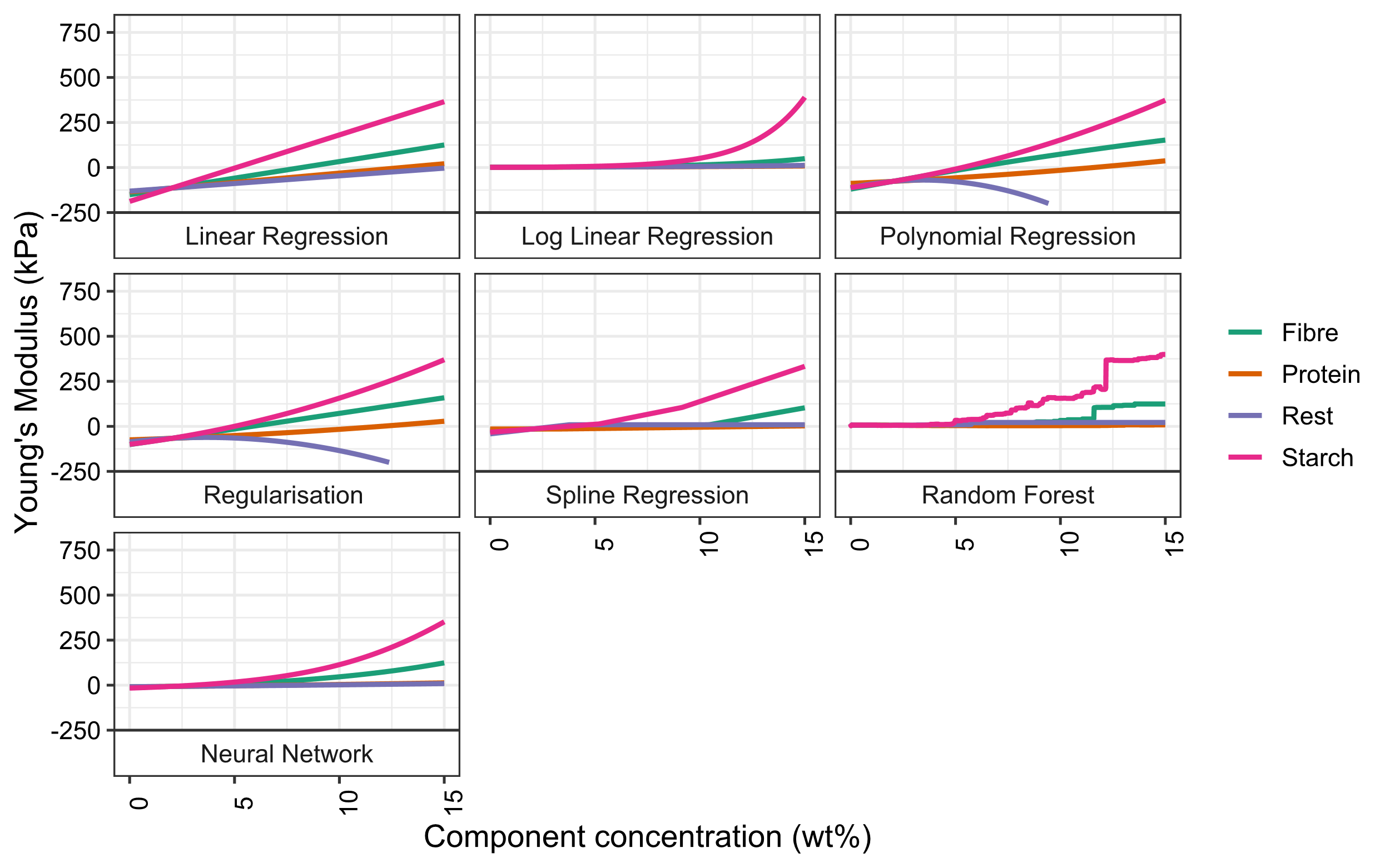


Figure 30 Scatterplot of the behaviour of each component in the evaluated models for quantifying the gel stiffness of yellow pea and lupine ingredients and mixtures of those with the main macro components as independent variables. The composition of each component increases from 1-15 wt% while the other stay constant at 2%.


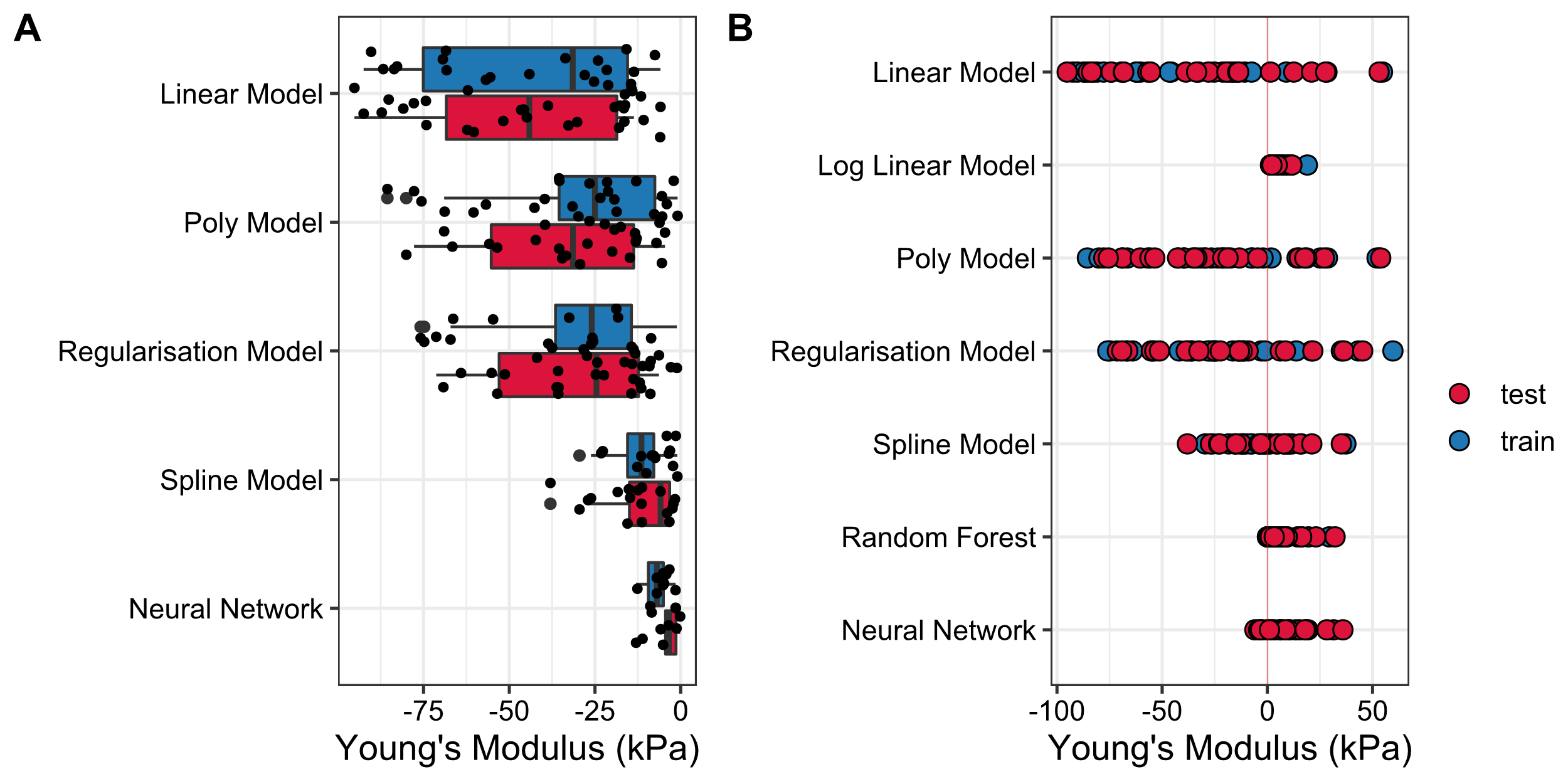


Figure 31 Scatter and boxplot of negative values (A) and predicted values for the datapoints of with an original value of zero (B) predicted by the evaluated models to for quantifying the gel stiffness of yellow pea and lupine ingredients and mixtures of those with the main macro components as independent variables.

## Heated viscosity

### Yellow pea

Based on the model metrics the spline regression is picked as the most suitable model. It also shows the most physically feasible behaviour out of all models. There are no significant differences between the sets and the main macro components without a split also overall give the lowest errors out of all sets of variables. The impact of fibre is very low but when it gets negative it is also outside of the measuring range.

Table 14 Model metrics models for quantifying heated viscosity with main macro components as independent variables for yellow pea ingredients.

| Model | RMSE Train | R2 Train | MAE Train | RMSE Test | R2 Test | MAE Test |
| --- | --- | --- | --- | --- | --- | --- |
| Linear Model | 1217.57 | 0.84 | 906.52 | 1371.44 | 0.72 | 1200.10 |
| Log Linear Model | 1847.20 | 0.81 | 806.04 | 1503.29 | 0.95 | 627.29 |
| Poly Model | 764.60 | 0.94 | 472.39 | 696.80 | 0.91 | 482.79 |
| Regularisation Model | 772.01 | 0.94 | 480.65 | 646.87 | 0.91 | 448.98 |
| Spline Model | 420.60 | 0.98 | 234.30 | 597.53 | 0.95 | 457.04 |
| Random Forest | 501.39 | 0.98 | 242.15 | 887.85 | 0.94 | 682.24 |
| Neural network | 411.68 | 0.98 | 215.28 | 730.16 | 0.99 | 440.95 |


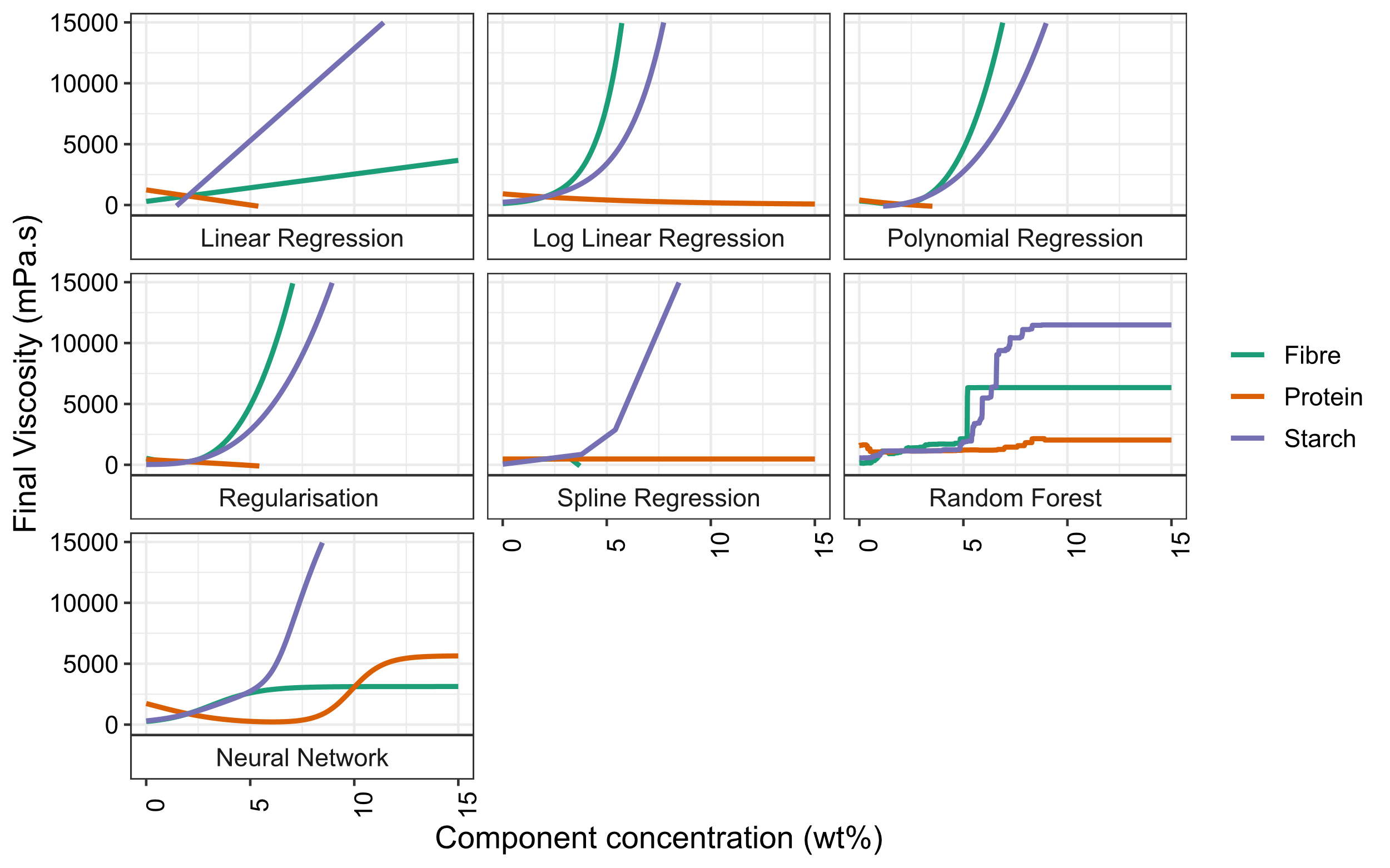


Figure 32 Scatterplot of the behaviour of each component in the evaluated models for quantifying the heated viscosity of yellow pea ingredients with the main macro components as independent variables. The composition of each component increases from 1-15 wt% while the other stay constant at 2%.


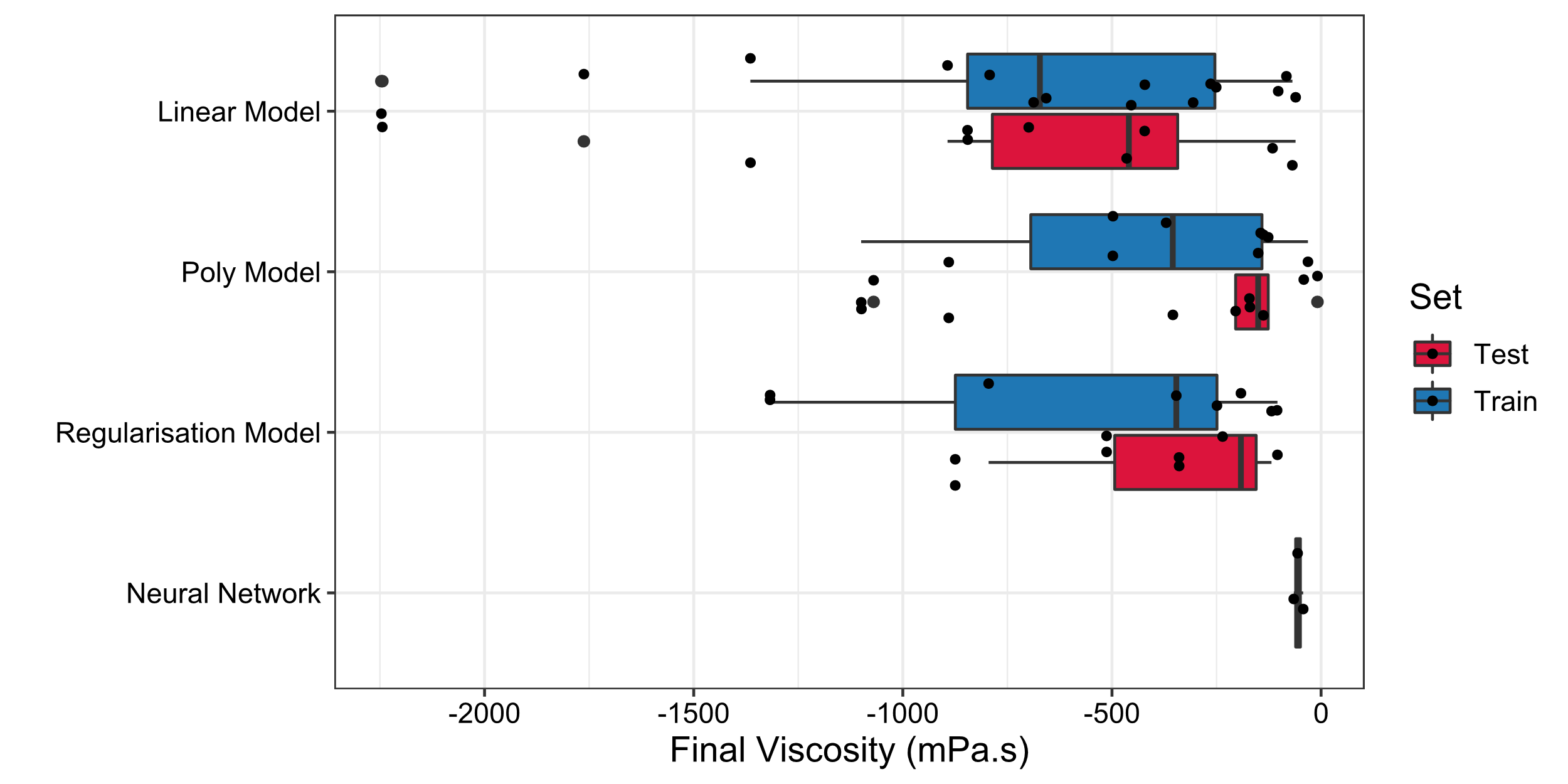


Figure 33 Boxplot of negative values predicted by the evaluated models to for quantifying the heated viscosity of yellow pea ingredients with the main macro components as independent variables.


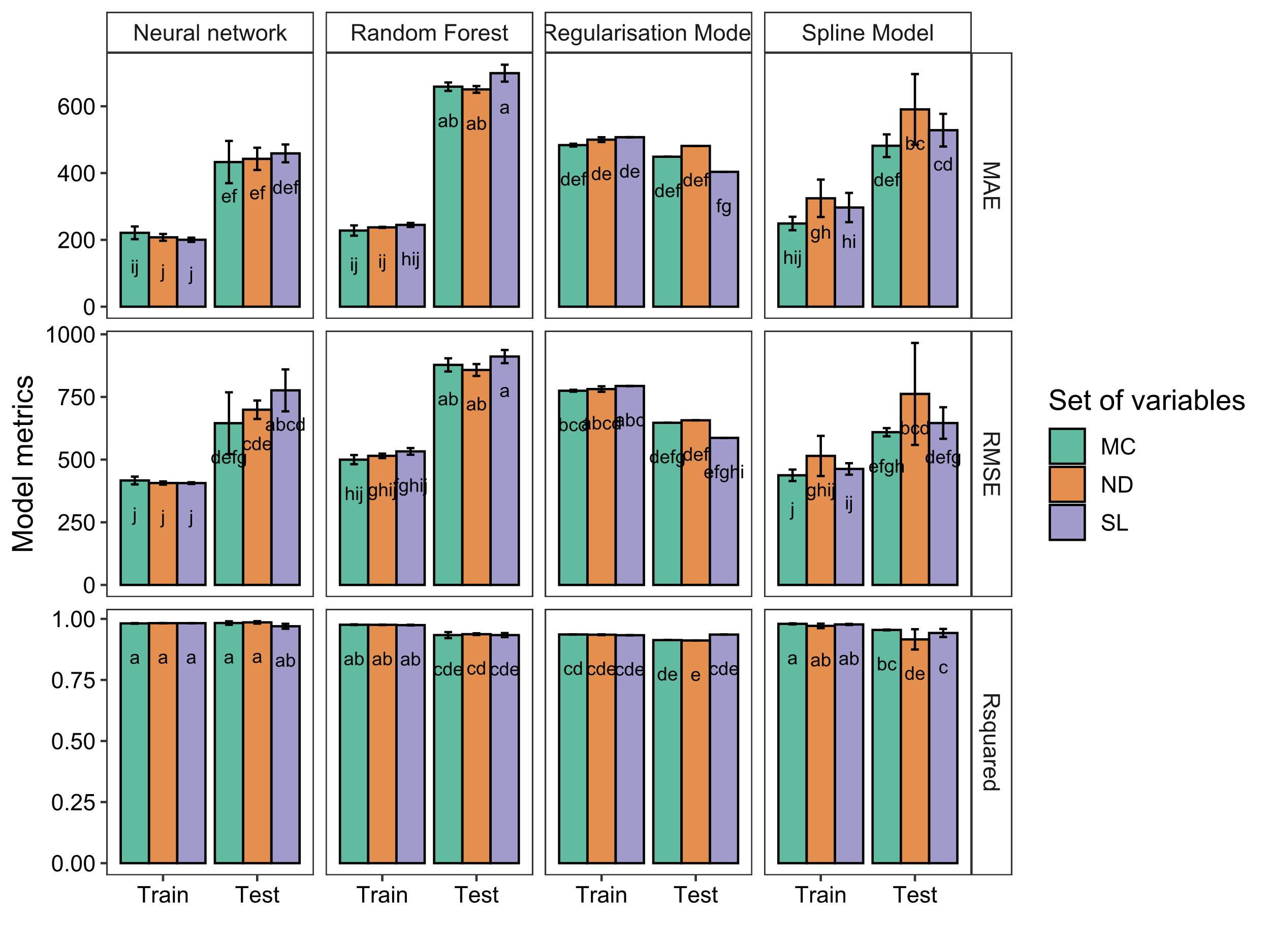


Figure 34 Bar chart containing the model metrics to predict the heated viscosity (mean absolute error (MAE), root mean square error (RMSE), and R^2^) generated five times for the neural network, random forest, and spline regression for yellow pea ingredients with the main macro components (MC) and main macro components with a split according to native proteins (ND) and soluble protein (SL) as independent variables. Letters indicate a significant different (P<0.05).

### Lupine

Based on the model metrics the neural network is clearly the best. Few negative values are predicted, and the behaviour trend is physically plausible. Lupine with a protein split according to solubility seems to result in substantially better test model metrics than with just the main macro components Therefore, best model is neural network with MC with a split according to solubility. Due to quite a big variation in the metrics of the obtained neural networks, the one with the lowest test error is used for further analyses.

Table 15 Model metrics models for quantifying heated viscosity with main macro components as independent variables for lupine ingredients.

| Model | RMSE Train | R2 Train | MAE Train | RMSE Test | R2 Test | MAE Test |
| --- | --- | --- | --- | --- | --- | --- |
| Linear Model | 596.39 | 0.88 | 415.37 | 1341.17 | 0.62 | 794.60 |
| Log Linear Model | 1007.92 | 0.79 | 347.61 | 1501.25 | 0.52 | 623.24 |
| Poly Model | 749.39 | 0.82 | 485.72 | 1111.65 | 0.74 | 781.64 |
| Regularisation Model | 750.82 | 0.82 | 476.17 | 1094.14 | 0.75 | 754.02 |
| Spline Model | 393.45 | 0.95 | 253.30 | 1302.99 | 0.63 | 615.21 |
| Random Forest | 416.30 | 0.96 | 238.97 | 1207.15 | 0.73 | 526.23 |
| Neural network | 420.01 | 0.94 | 274.12 | 996.98 | 0.79 | 550.75 |

Table 16 Model metrics models for quantifying heated viscosity with main macro components with a split according to protein solubility as independent variables for lupine ingredients.

| Model | RMSE Train | R2 Train | MAE Train | RMSE Test | R2 Test | MAE Test |
| --- | --- | --- | --- | --- | --- | --- |
| Linear Model | 531.07 | 0.91 | 386.83 | 1055.70 | 0.84 | 570.49 |
| Log Linear Model | 551.35 | 0.92 | 253.19 | 1052.99 | 0.71 | 485.94 |
| Poly Model | 530.06 | 0.91 | 364.20 | 1072.58 | 0.71 | 846.40 |
| Regularisation Model | 834.49 | 0.78 | 521.64 | 1062.17 | 0.82 | 625.22 |
| Spline Model | 428.25 | 0.94 | 282.76 | 1224.75 | 0.74 | 548.10 |
| Random Forest | 458.21 | 0.95 | 253.45 | 1133.20 | 0.78 | 509.93 |
| Neural network | 353.93 | 0.96 | 224.50 | 547.07 | 0.93 | 280.73 |


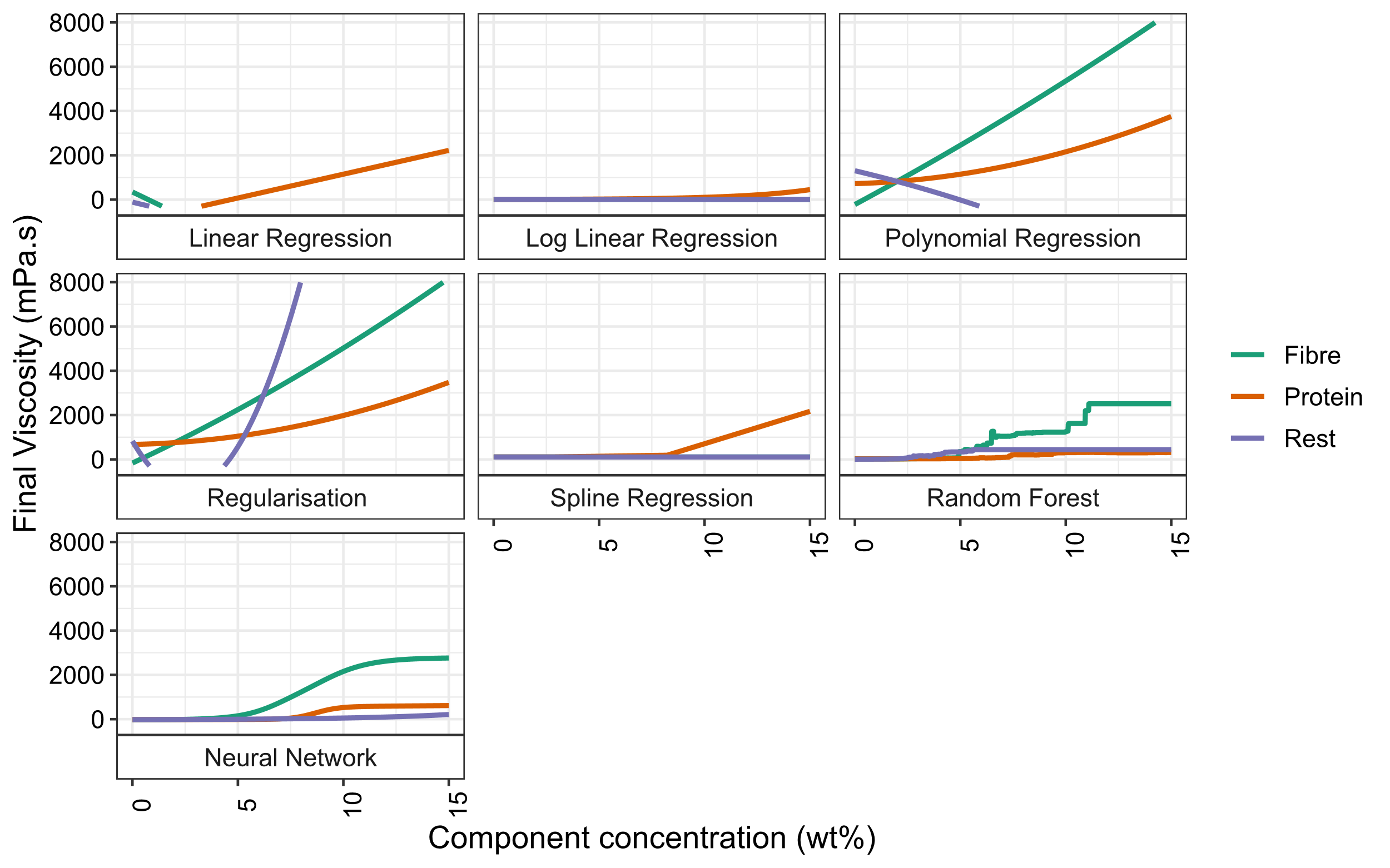


Figure 35 Scatterplot of the behaviour of each component in the evaluated models for quantifying the heated viscosity of lupine ingredients with the main macro components as independent variables. The composition of each component increases from 1-15 wt% while the other stay constant at 2%.


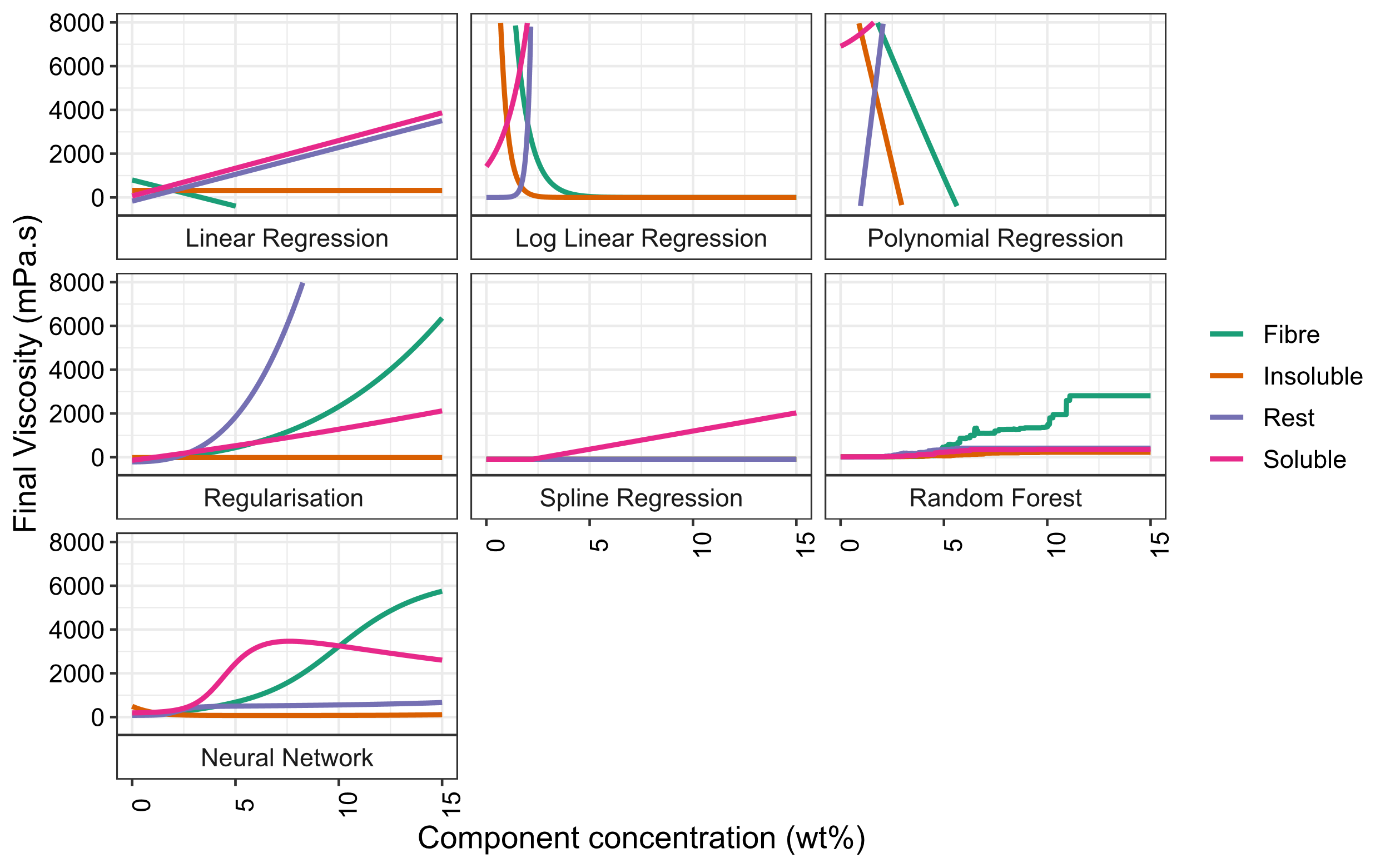


Figure 36 Scatterplot of the behaviour of each component in the evaluated models for quantifying the heated viscosity of lupine ingredients with the main macro components with a split according to protein solubility as independent variables. The composition of each component increases from 1-15 wt% while the other stay constant at 2%.


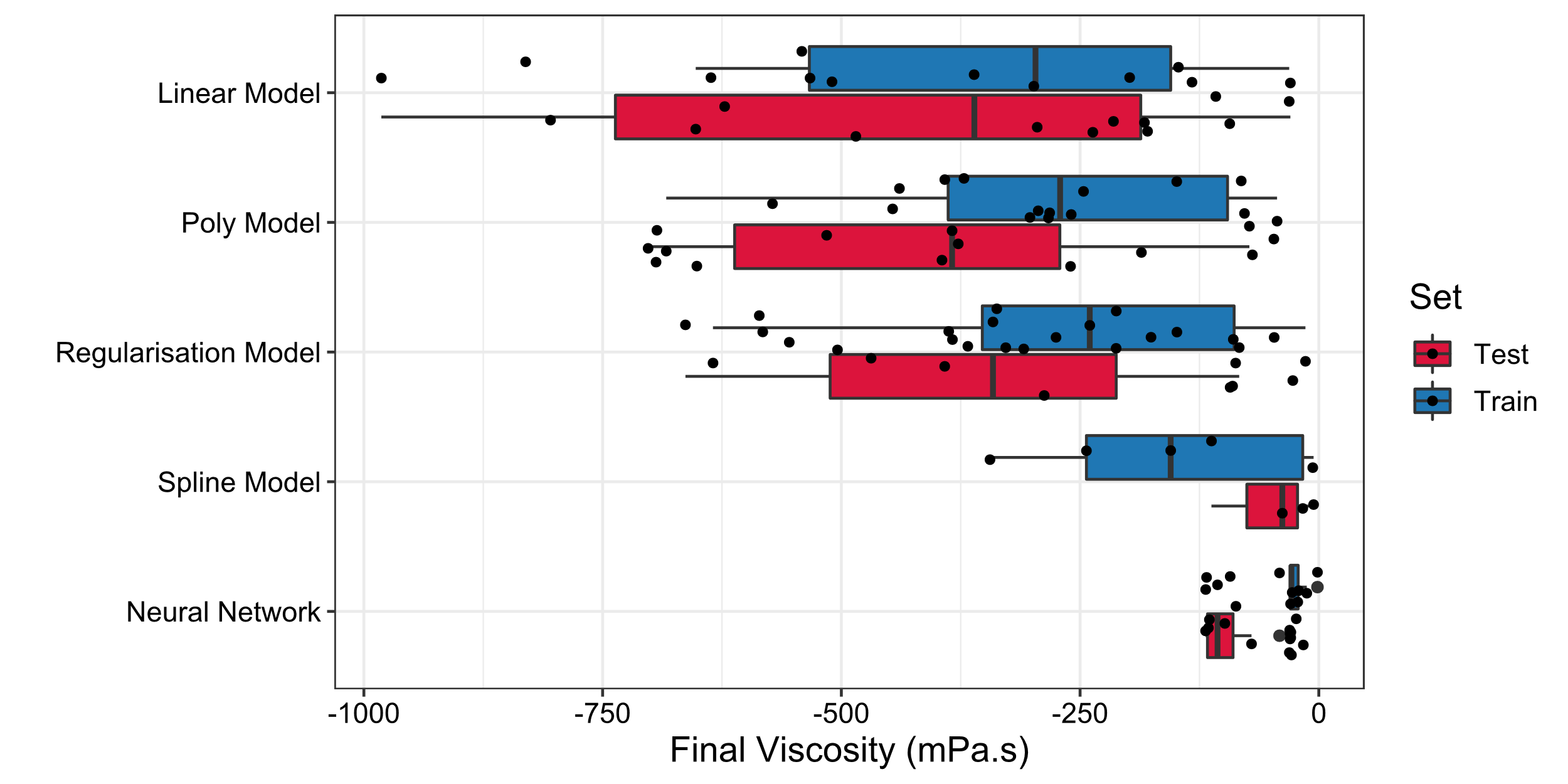


Figure 37 Boxplot of negative values predicted by the evaluated model for quantifying the heated viscosity of lupine ingredients with the main macro components as independent variables.


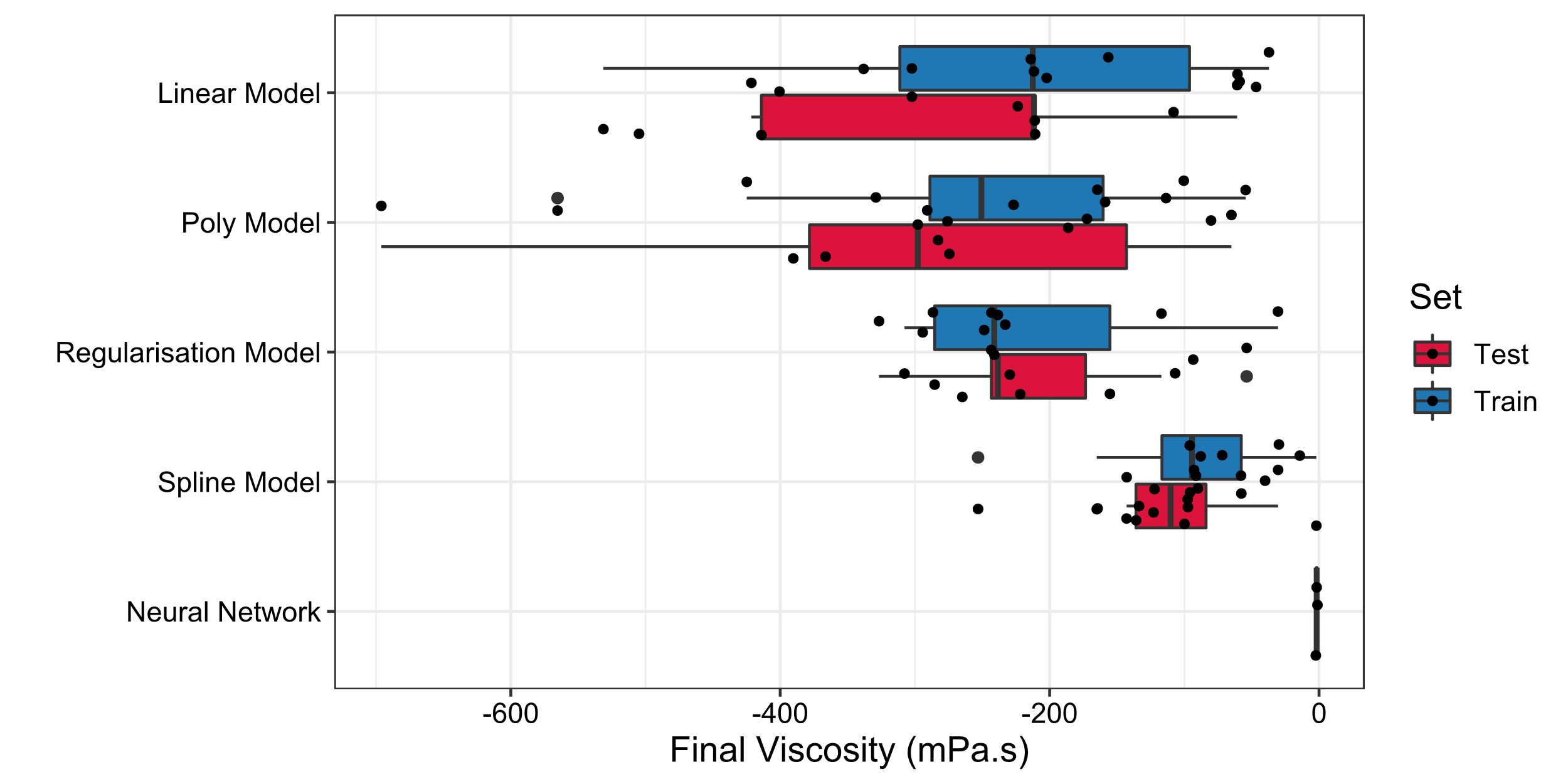


Figure 38 Boxplot of negative values predicted by the evaluated models for quantifying the heated viscosity of lupine ingredients with the main macro components with a split according to protein solubility as independent variables.


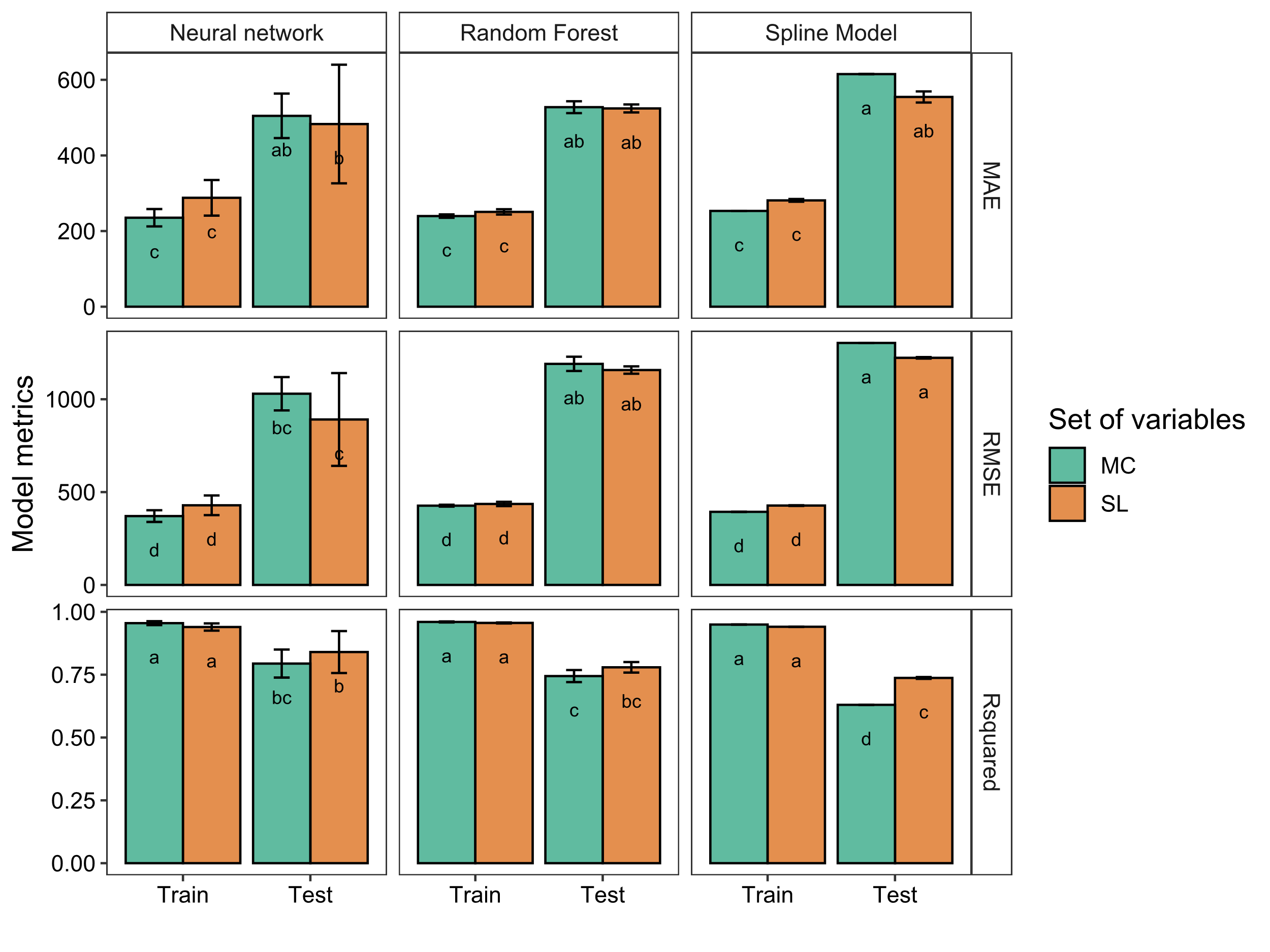


Figure 39 Bar chart containing the model metrics to predict the heated viscosity (mean absolute error (MAE), root mean square error (RMSE), and R^2^) generated five times for the neural network, random forest, and spline regression for lupine ingredients with the main macro components (MC) and main macro components with a split according to soluble protein (SL) as independent variables. Letters indicate a significant different (P<0.05).

### All data combined

As for lupine the heated viscosity was better predicted with a split in protein according to solubility, this split is also evaluated for the model based on all data. However, no significant difference in model metrics was found, therefore the main macro components will be used as independent variables. In terms of model metrics for the main macro components as independent variables, the spline regression, polynomial regression, and regularised polynomial linear regression are of interest. The latter two produce more negative values. Therefore, the spline regression will be considered the best.

*Table 17 Model metrics models for quantifying heated viscosity with main macro components as independent variables for yellow pea, lupine and mixtures of those.*

| Model | RMSE Train | R2 Train | MAE Train | RMSE Test | R2 Test | MAE Test |
| --- | --- | --- | --- | --- | --- | --- |
| Linear Model | 1708.28 | 0.74 | 1209.57 | 5364.40 | 0.84 | 2158.29 |
| Log Linear Model | 3233.94 | 0.79 | 1245.82 | 3322147.11 | 0.55 | 482785.18 |
| Poly Model | 1799.56 | 0.71 | 1093.31 | 1637.99 | 0.96 | 1183.49 |
| Regularisation Model | 1808.16 | 0.71 | 1086.40 | 1630.75 | 0.96 | 1110.23 |
| Spline Model | 822.83 | 0.94 | 554.89 | 1960.03 | 0.96 | 1076.79 |
| Random Forest | 735.93 | 0.96 | 316.00 | 5905.49 | 0.71 | 1886.25 |
| Neural network | 831.84 | 0.94 | 544.63 | 4076.88 | 0.76 | 2524.28 |


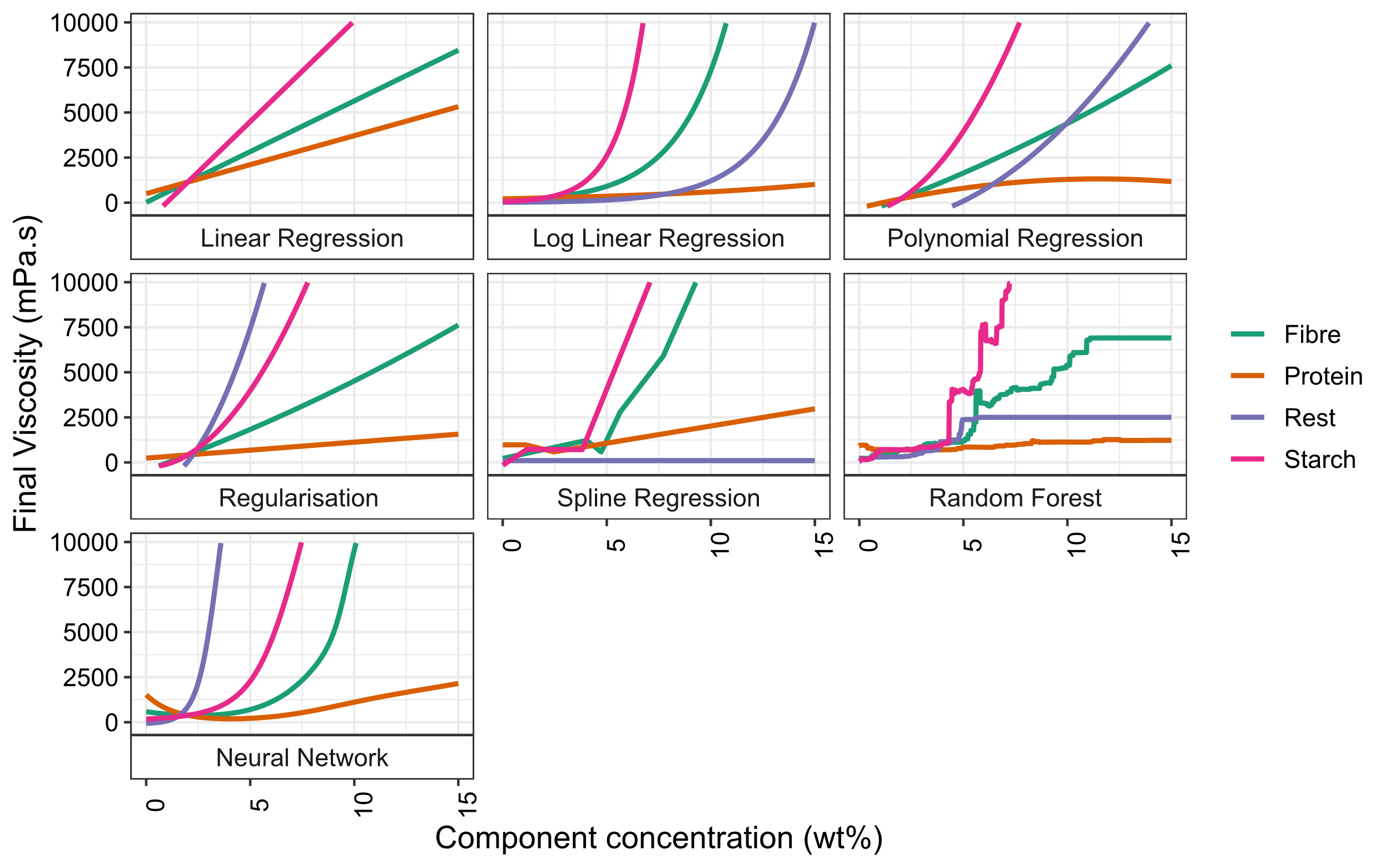


Figure 40 Scatterplot of the behaviour of each component in the evaluated models for quantifying the heated viscosity of yellow pea and lupine ingredients and mixtures of those with the main macro components as independent variables. The composition of each component increases from 1-15 wt% while the other stay constant at 2%.


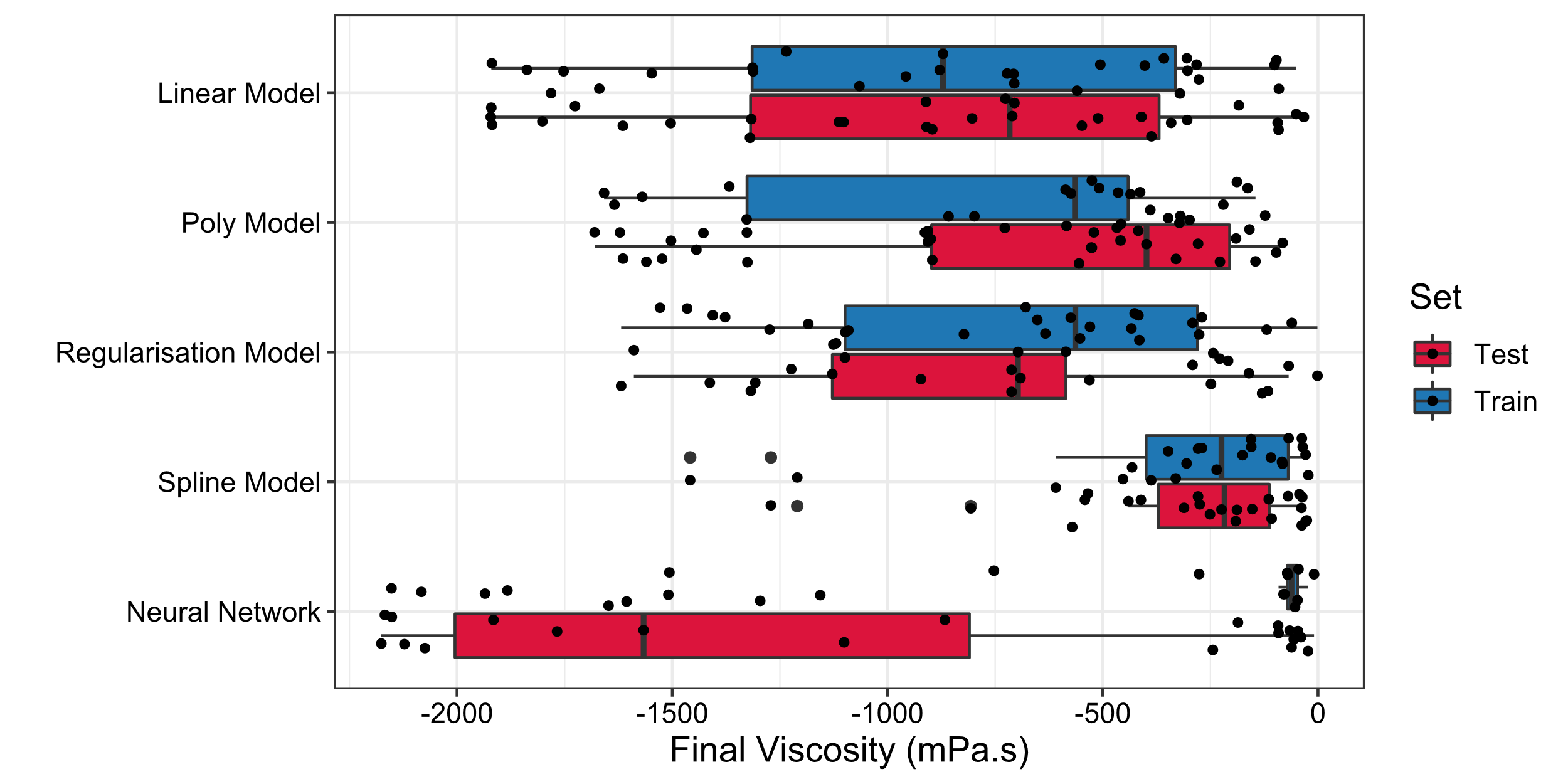


Figure 41 Boxplot of negative values predicted by the evaluated models to for quantifying the heated viscosity of yellow pea and lupine ingredients and mixtures of those with the main macro components as independent variables.


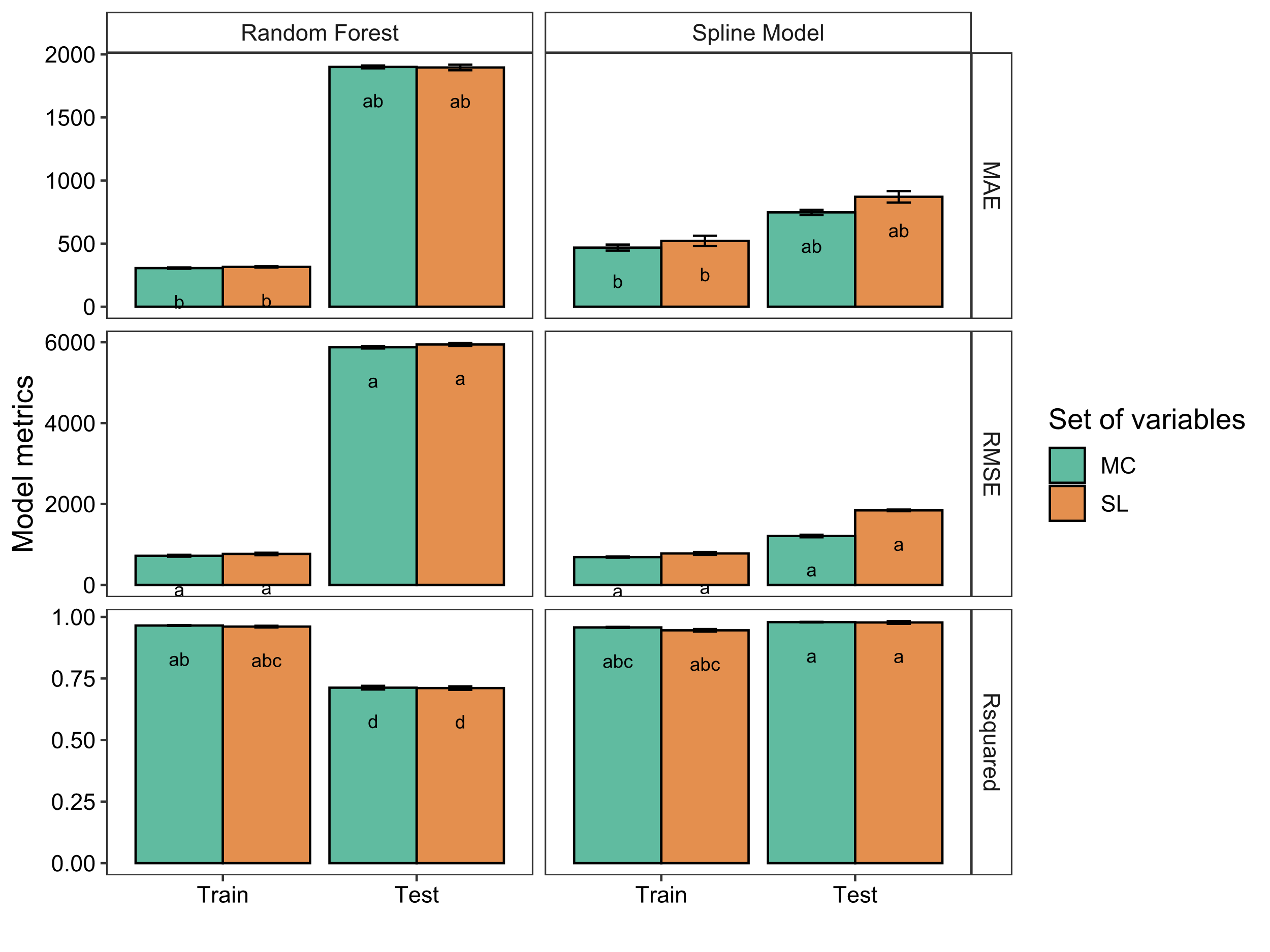


Figure 42 Bar chart containing the model metrics to predict the heated viscosity (mean absolute error (MAE), root mean square error (RMSE), and R^2^) generated five times for the random forest, and spline regression for yellow pea and lupine ingredients and mixtures of those with the main macro components (MC) and main macro components with a split according to soluble protein (SL) as independent variables. Letters indicate a significant different (P<0.05). The neural network was left out for clarity since it has relatively big variation.

## Unheated viscosity

### Yellow pea

In terms of model metrics, the spline regression, random forest, polynomial, and regularized polynomial regression are of interest. The polynomial, spline regression show physically unplausible behaviour and are therefore not considered. The behaviour of the regularized polynomial linear regression is more feasible than that of the random forest and produces fewer negative values than the spline model. Although with split in protein according to nativity or solubility the metrics are slightly better in a regularized polynomial linear regression, the behaviour scatterplots show trend that goes negative values and therefore also more negative values are observed in the boxplot. Therefore, the regularisation with the main macro components is considered most suitable for this dataset.

Table 18 Model metrics models for quantifying unheated viscosity with main macro components as independent variables for yellow pea ingredients.

| Model | RMSE Train | R2 Train | MAE Train | RMSE Test | R2 Test | MAE Test |
| --- | --- | --- | --- | --- | --- | --- |
| Linear Model | 63.50 | 0.54 | 33.42 | 91.44 | 0.91 | 56.81 |
| Log Linear Model | 58.46 | 0.85 | 16.47 | 112.67 | 1.00 | 51.86 |
| Poly Model | 38.38 | 0.83 | 24.38 | 45.01 | 0.94 | 31.55 |
| Regularisation Model | 52.47 | 0.71 | 21.95 | 69.86 | 0.99 | 36.05 |
| Spline Model | 54.43 | 0.66 | 23.42 | 37.94 | 1.00 | 24.38 |
| Random Forest | 37.68 | 0.92 | 12.19 | 77.37 | 1.00 | 40.60 |
| Neural network | 6.98 | 0.99 | 4.24 | 224.68 | 1.00 | 109.01 |


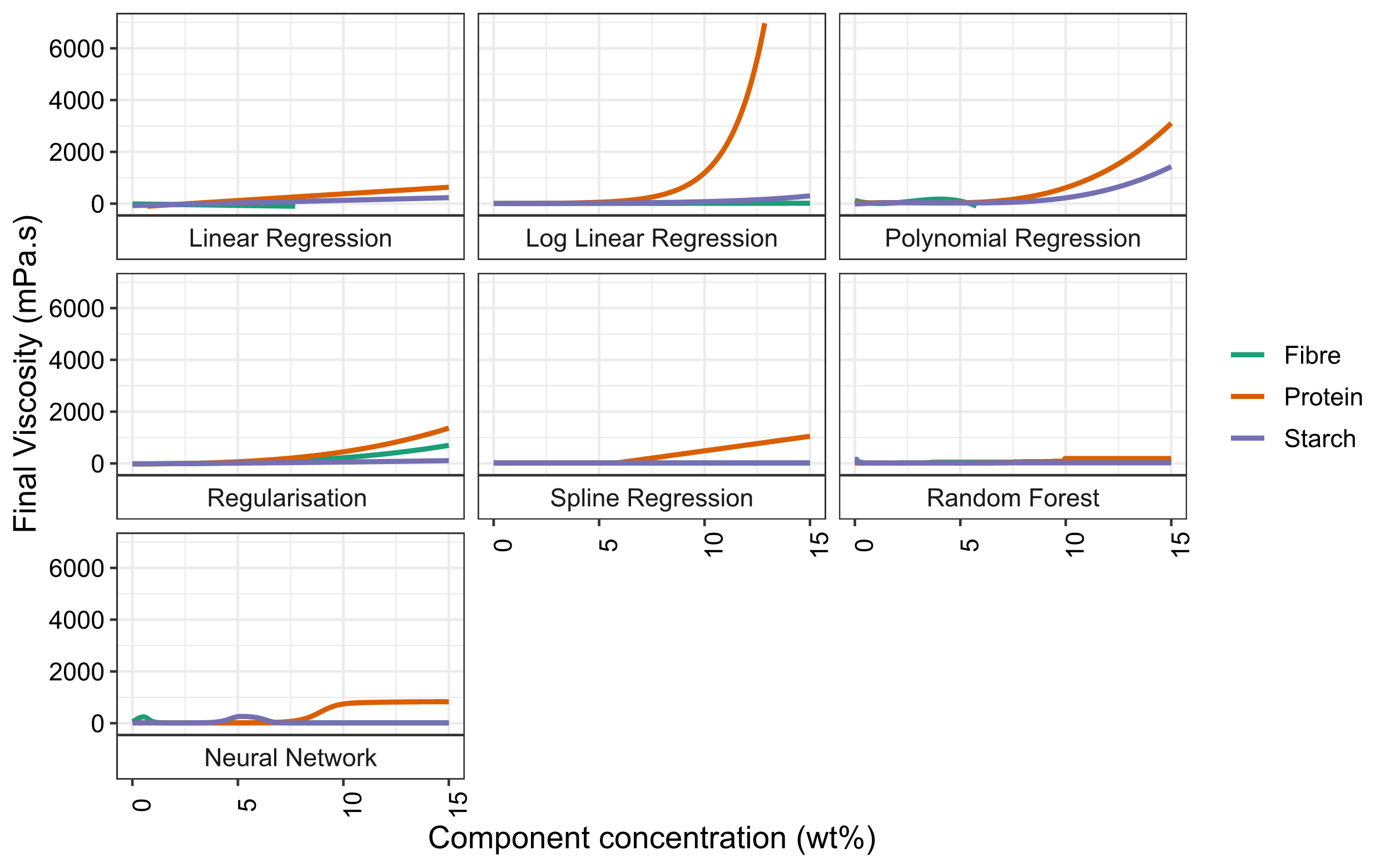


Figure 43 Scatterplot of the behaviour of each component in the evaluated models for quantifying the unheated viscosity of yellow pea ingredients with the main macro components as independent variables. The composition of each component increases from 1-15 wt% while the other stay constant at 2%.


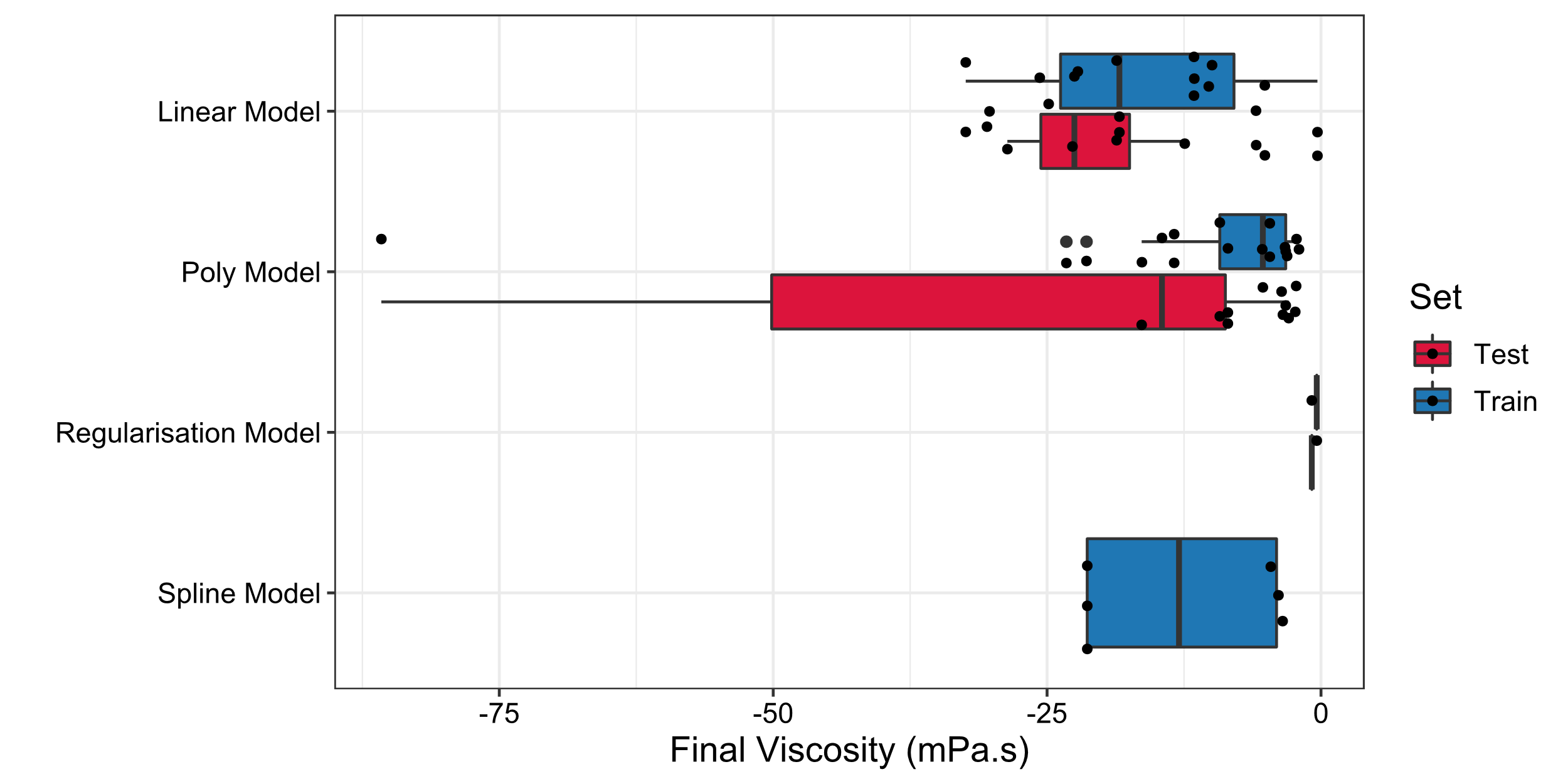


Figure 44 Boxplot of negative values predicted by the evaluated models to for quantifying the unheated viscosity of yellow pea ingredients with the main macro components as independent variables.


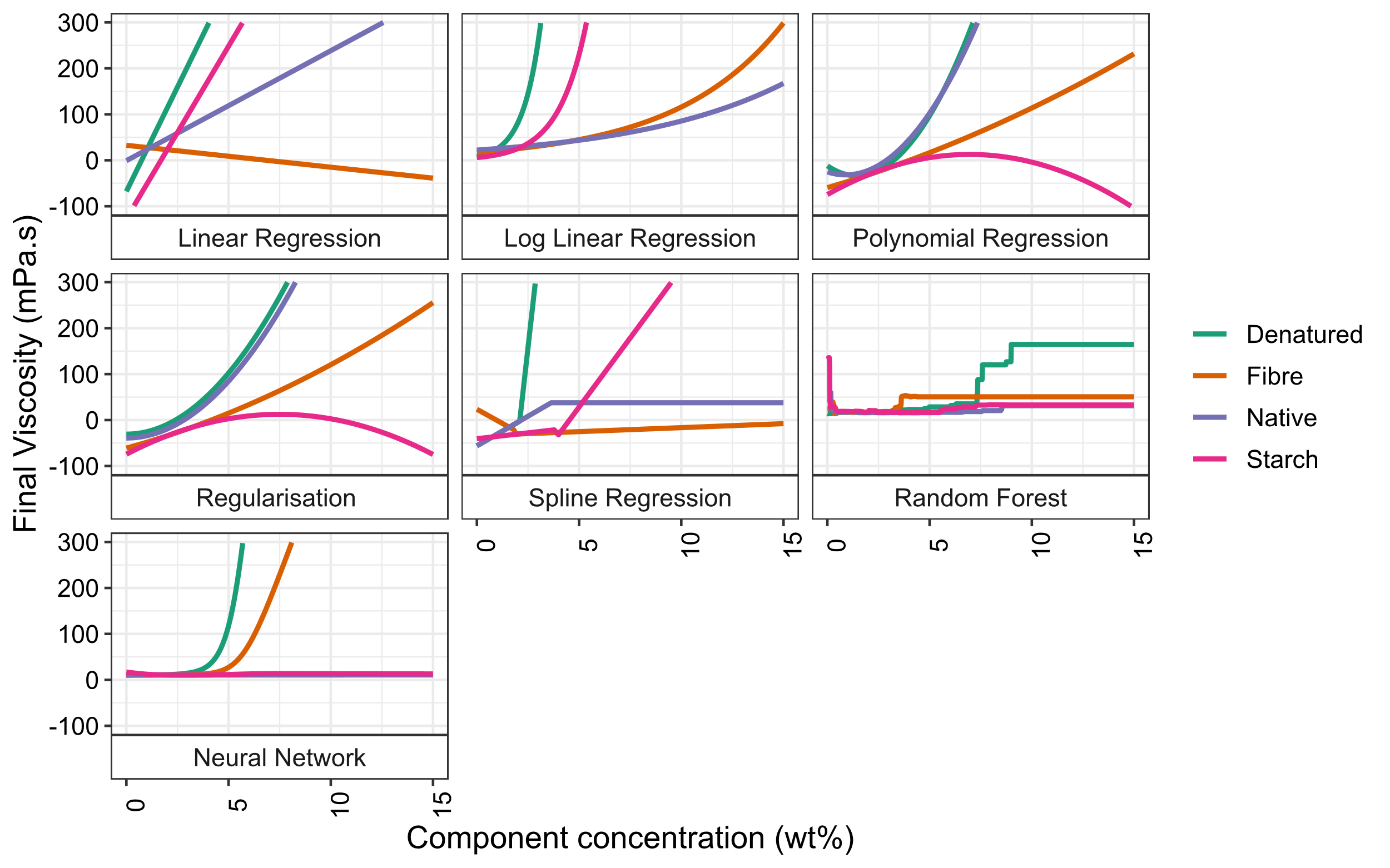


Figure 45 Scatterplot of the behaviour of each component in the evaluated models for quantifying the unheated viscosity of yellow pea ingredients with the main macro components as independent variables with a split according to nativity. The composition of each component increases from 1-15 wt% while the other stay constant at 2%.


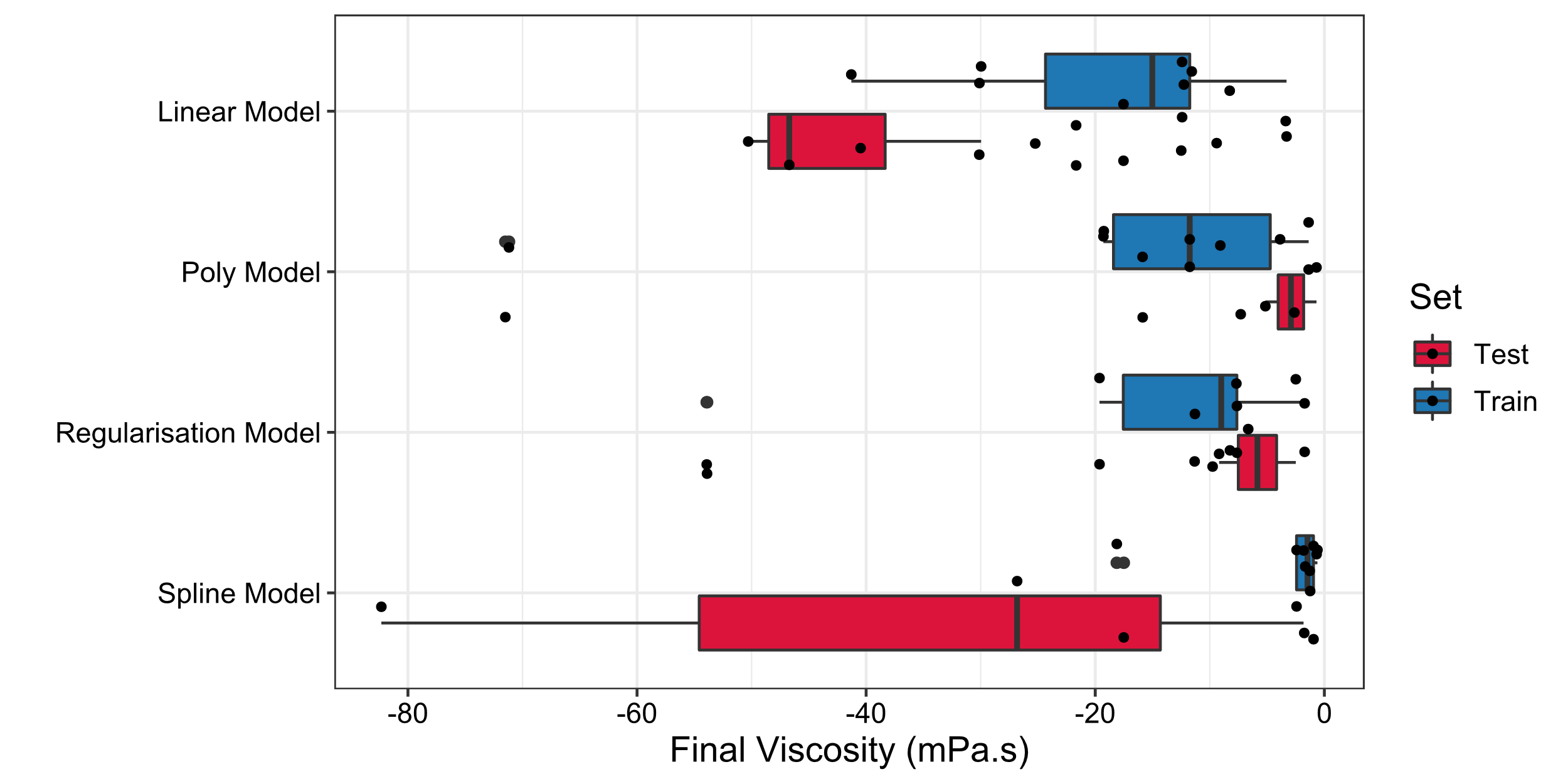


Figure 46 Boxplot of negative values predicted by the evaluated models to for quantifying the unheated viscosity of yellow pea ingredients with the main macro components as independent variables with a split according to nativity.


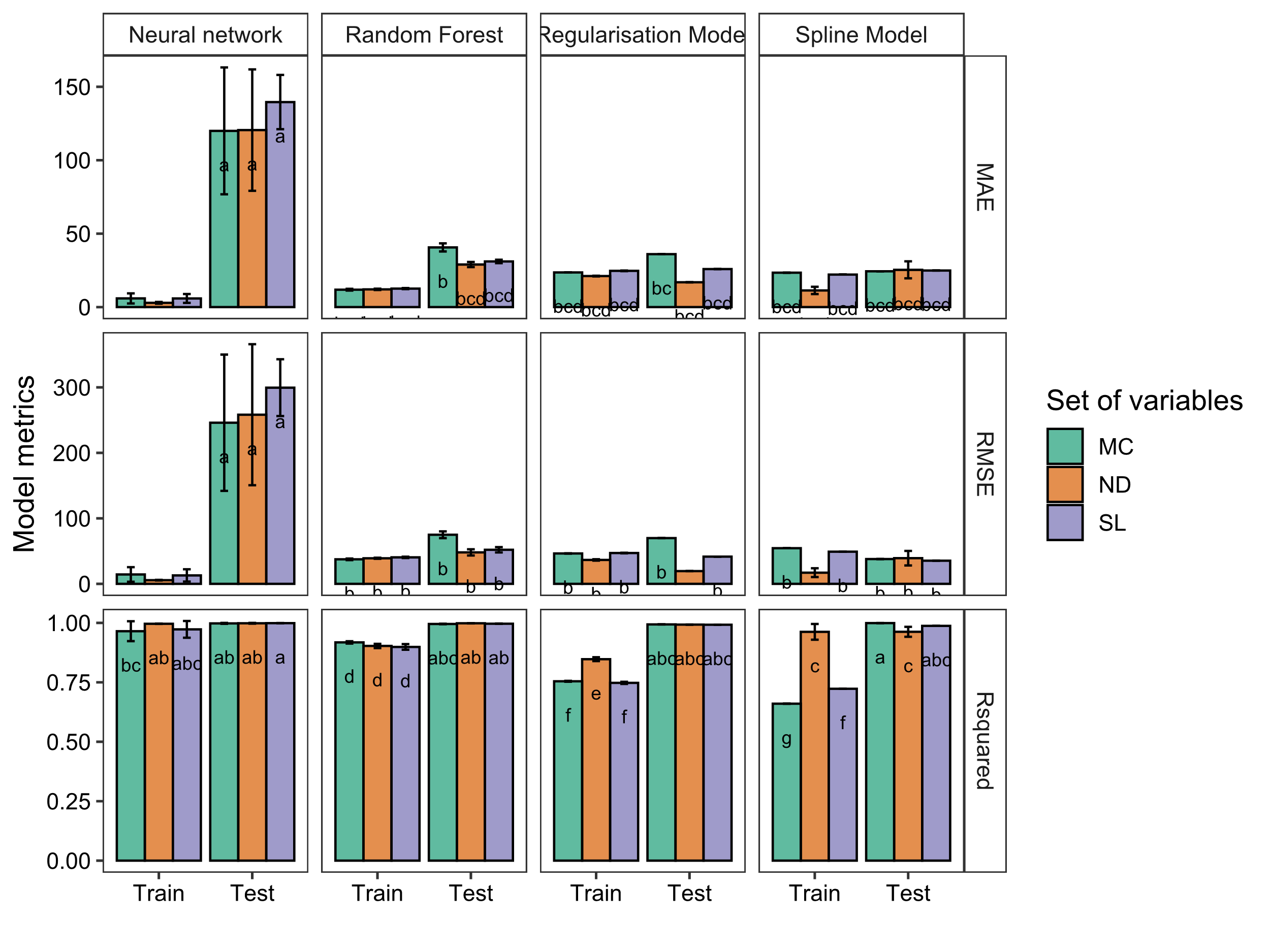


Figure 47 Bar chart containing the model metrics to predict the unheated viscosity (mean absolute error (MAE), root mean square error (RMSE), and R^2^) generated five times for the neural network, random forest, and spline regression for yellow pea ingredients with the main macro components (MC) and main macro components with a split according to native proteins (ND) and soluble protein (SL) as independent variables. Letters indicate a significant different (P<0.05).

### Lupine

From the model metrics it is derived that the neural network is the best candidate for quantifying unheated viscosity of lupine ingredients. The behaviour scatterplots shows physically plausible behaviour in terms of trends and negative values for the neural network. There are no significant differences between the test metrics between the base components and the split according to solubility. Therefore, the best model is the neural network with main macro components. As there is quite a large variation in the five times repeated neural networks, the one with the lowest test error is chosen for further analyses.

Table 19 Model metrics models for quantifying unheated viscosity with main macro components as independent variables using lupine ingredients.

| Model | RMSE Train | R2 Train | MAE Train | RMSE Test | R2 Test | MAE Test |
| --- | --- | --- | --- | --- | --- | --- |
| Linear Model | 494.73 | 0.80 | 297.56 | 482.37 | 0.59 | 333.00 |
| Log Linear Model | 721.84 | 0.59 | 179.01 | 295.52 | 0.65 | 94.52 |
| Poly Model | 212.58 | 0.96 | 158.54 | 349.74 | 0.78 | 215.63 |
| Regularisation Model | 445.65 | 0.84 | 214.43 | 467.02 | 0.64 | 278.70 |
| Spline Model | 220.66 | 0.96 | 124.12 | 352.40 | 0.68 | 201.38 |
| Random Forest | 308.43 | 0.95 | 92.37 | 344.78 | 0.80 | 130.84 |
| Neural network | 143.59 | 0.98 | 77.99 | 248.52 | 0.92 | 115.63 |


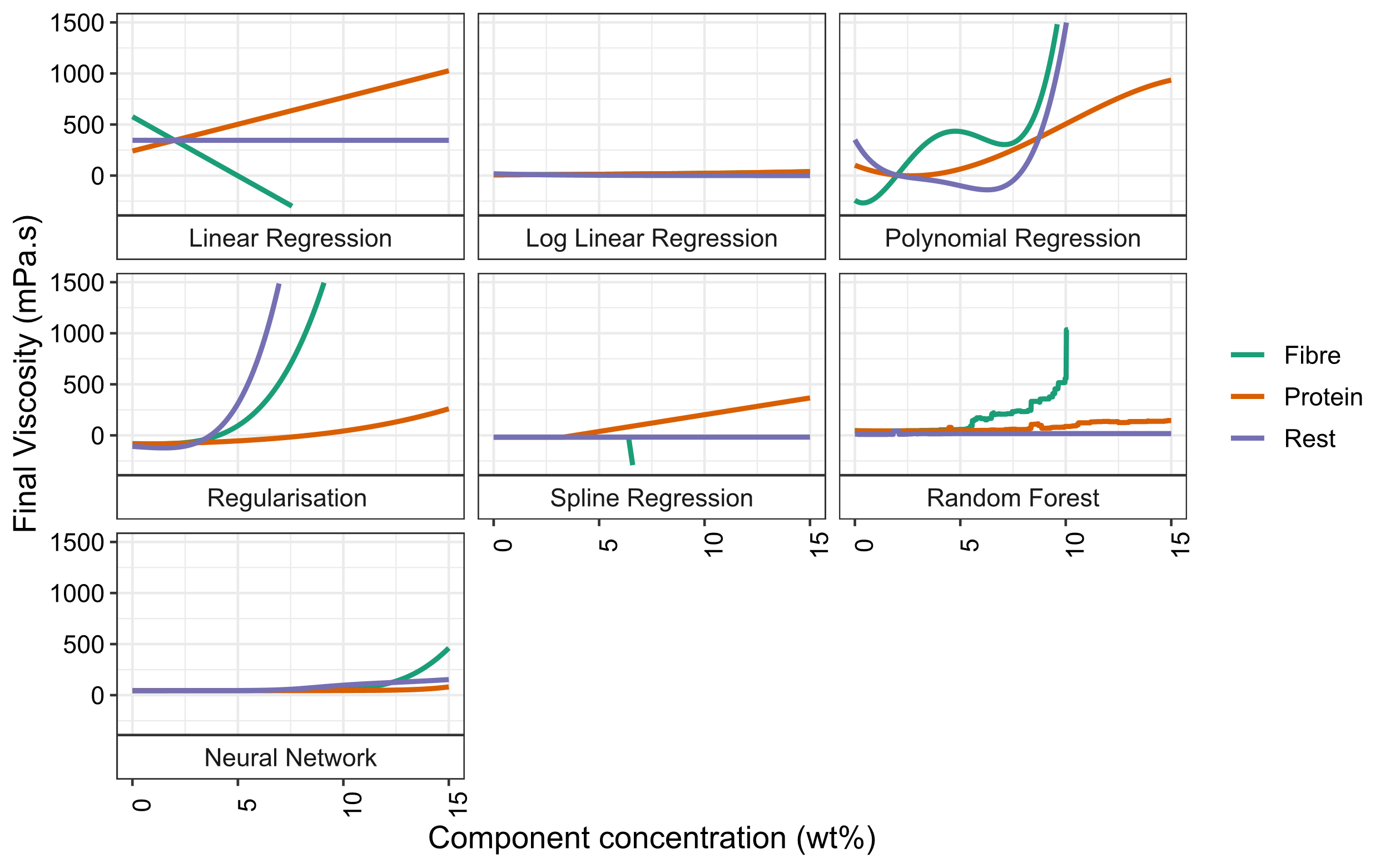


Figure 48 Scatterplot of the behaviour of each component in the evaluated models for quantifying the unheated viscosity of lupine ingredients with the main macro components as independent variables. The composition of each component increases from 1-15 wt% while the other stay constant at 2%.


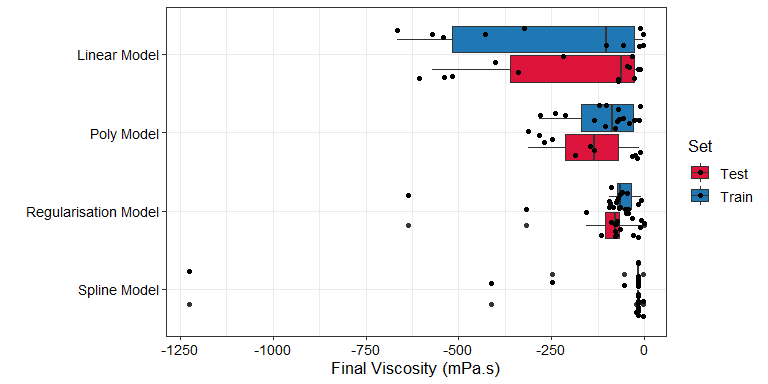


Figure 49 Boxplot of negative values predicted by the evaluated models to for quantifying the unheated viscosity of lupine ingredients with the main macro components as independent variables.


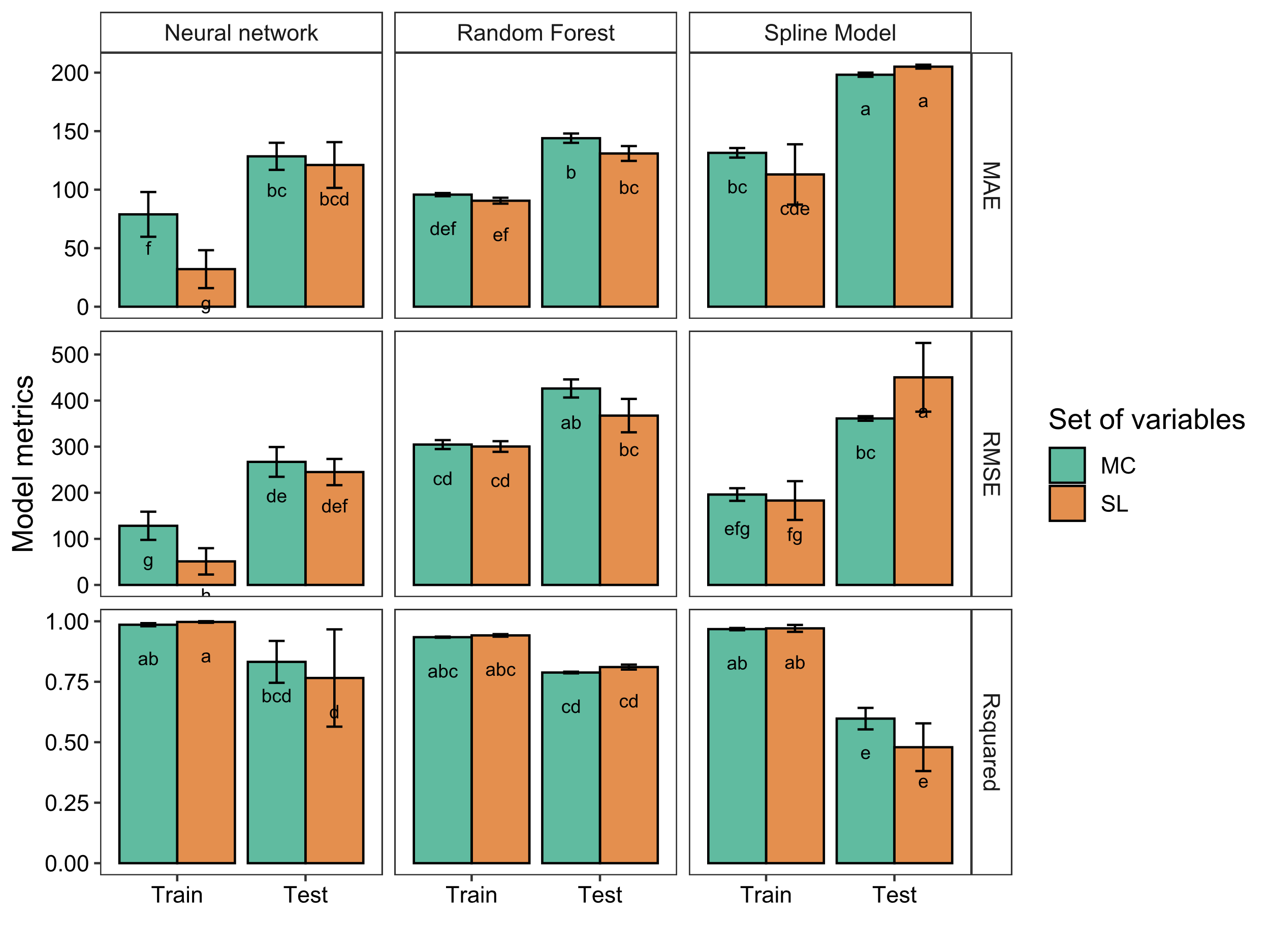


Figure 50 Bar chart containing the model metrics to predict the unheated viscosity (mean absolute error (MAE), root mean square error (RMSE), and R^2^) generated five times for the neural network, random forest, and spline regression for lupine ingredients with the main macro components (MC) and main macro components with a split according to soluble protein (SL) as independent variables. Letters indicate a significant different (P<0.05).

### All data combined

The data from the models created with and without a split in protein and fibre according to origin is presented here. The neural network has the lowest test errors together with the log linear model for the model without the split. Nevertheless, the log linear is not feasible because it increases too fast which results into unrealistic values. Therefore, the neural model is selected. As there was quite a large variation in the five times repeated neural networks (data not shown), the one with the lowest test error is chosen for further analyses.

Table 20 Model metrics models for quantifying unheated viscosity with main macro components as independent variables for data of ingredients from yellow pea, lupine, and mixtures of those.

| Model | RMSE Train | R2 Train | MAE Train | RMSE Test | R2 Test | MAE Test |
| --- | --- | --- | --- | --- | --- | --- |
| Linear Model | 508.64 | 0.39 | 252.74 | 408.32 | 0.31 | 277.27 |
| Log Linear Model | 549.24 | 0.54 | 105.57 | 205.11 | 0.62 | 77.30 |
| Poly Model | 368.98 | 0.68 | 199.95 | 400.28 | 0.45 | 232.96 |
| Regularisation Model | 377.01 | 0.67 | 186.88 | 382.76 | 0.40 | 213.40 |
| Spline Model | 300.68 | 0.79 | 110.22 | 408.85 | 0.53 | 179.17 |
| Random Forest | 192.81 | 0.94 | 54.54 | 221.69 | 0.68 | 107.08 |
| Neural network | 120.32 | 0.97 | 48.49 | 142.02 | 0.83 | 100.58 |


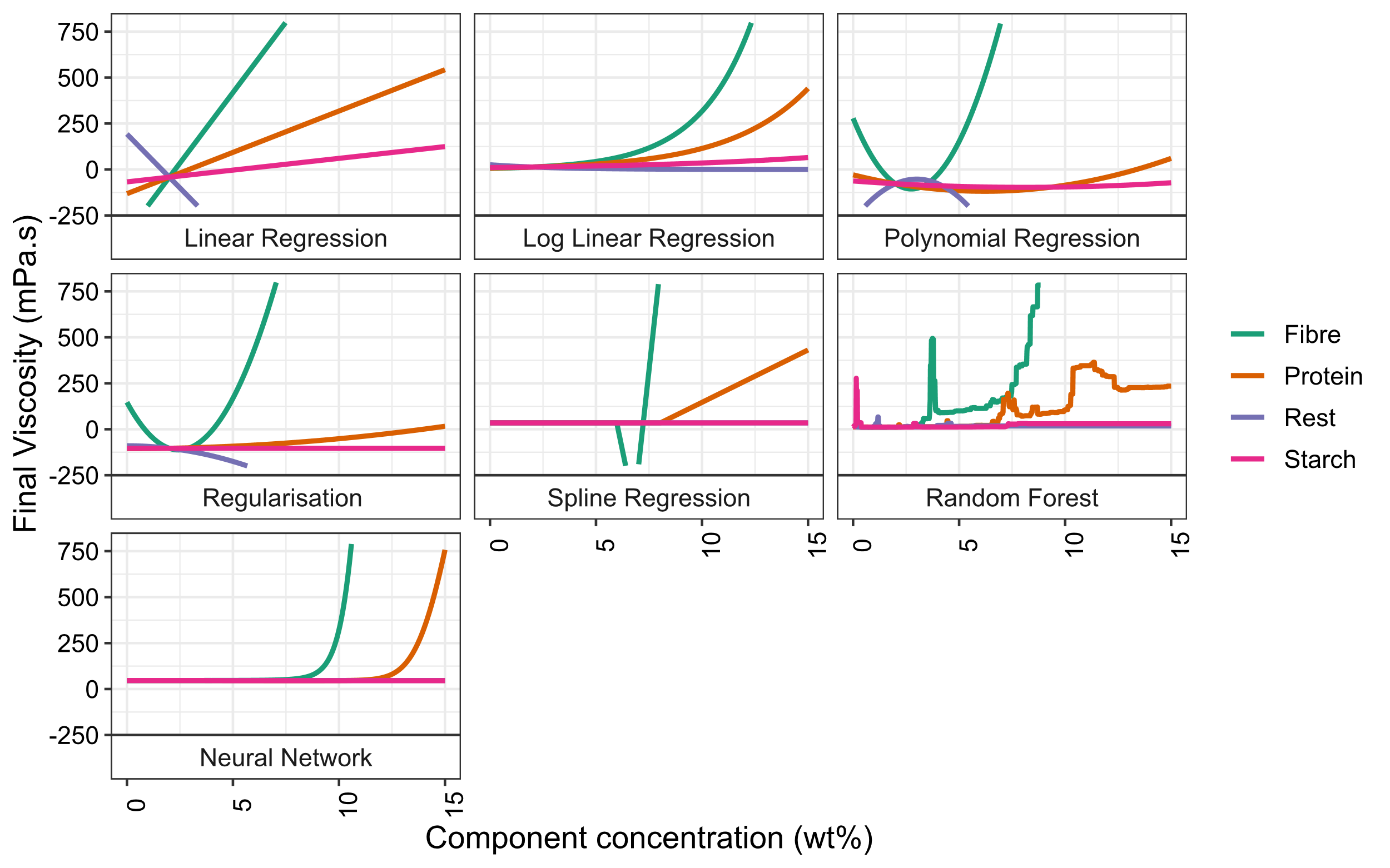


Figure 51 Scatterplot of the behaviour of each component in the evaluated models for quantifying the unheated viscosity of both yellow pea, lupine, and mixtures of those with the main macro components as independent variables. The composition of each component increases from 1-15 wt% while the other stay constant at 2%.


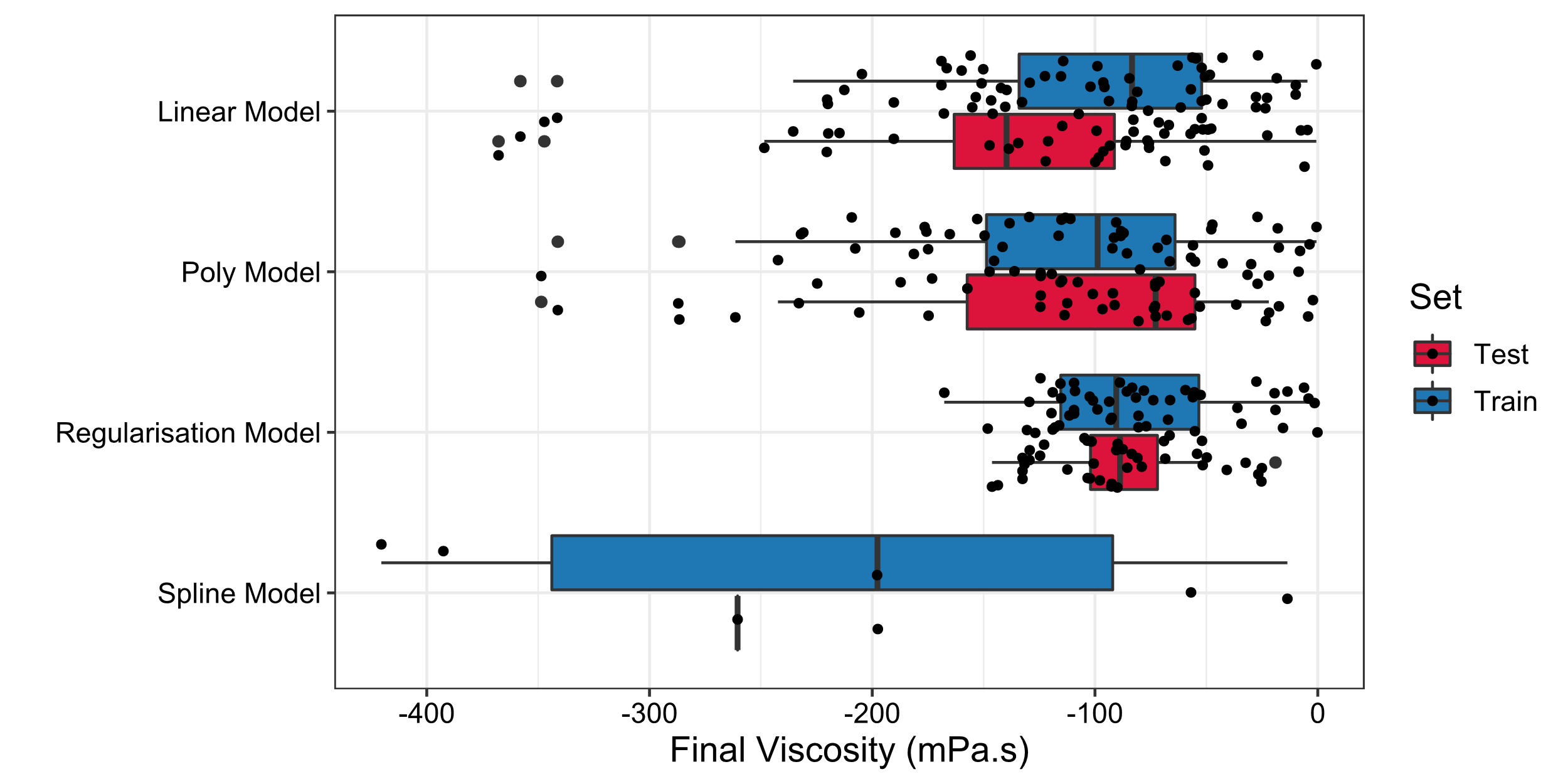


Figure 52 Boxplot of negative values predicted by the evaluated models to for quantifying the unheated viscosity both yellow pea, lupine, and mixtures of those with the main macro components as independent variables.

### All data combined with a split

The model picked with the dataset without a split is picked here as well.

Table 21 Model metrics models for quantifying unheated viscosity with main macro components as independent variables for data of ingredients from yellow pea, lupine, and mixtures of those, combined with a split according to origin (yellow pea of lupine).

| Model | RMSE Train | R2 Train | MAE Train | RMSE Test | R2 Test | MAE Test |
| --- | --- | --- | --- | --- | --- | --- |
| Linear Model | 498.37 | 0.42 | 244.14 | 440.83 | 0.27 | 332.44 |
| Log Linear Model | 466.35 | 0.65 | 88.26 | 174.93 | 0.70 | 75.41 |
| Poly Model | 349.03 | 0.71 | 168.12 | 534.22 | 0.40 | 305.64 |
| Regularisation Model | 361.74 | 0.69 | 153.70 | 426.39 | 0.43 | 220.47 |
| Spline Model | 336.38 | 0.73 | 106.84 | 467.49 | 0.44 | 192.64 |
| Random Forest | 165.12 | 0.95 | 50.07 | 416.52 | 0.63 | 138.08 |
| Neural network | 102.93 | 0.98 | 32.29 | 203.66 | 0.79 | 110.86 |


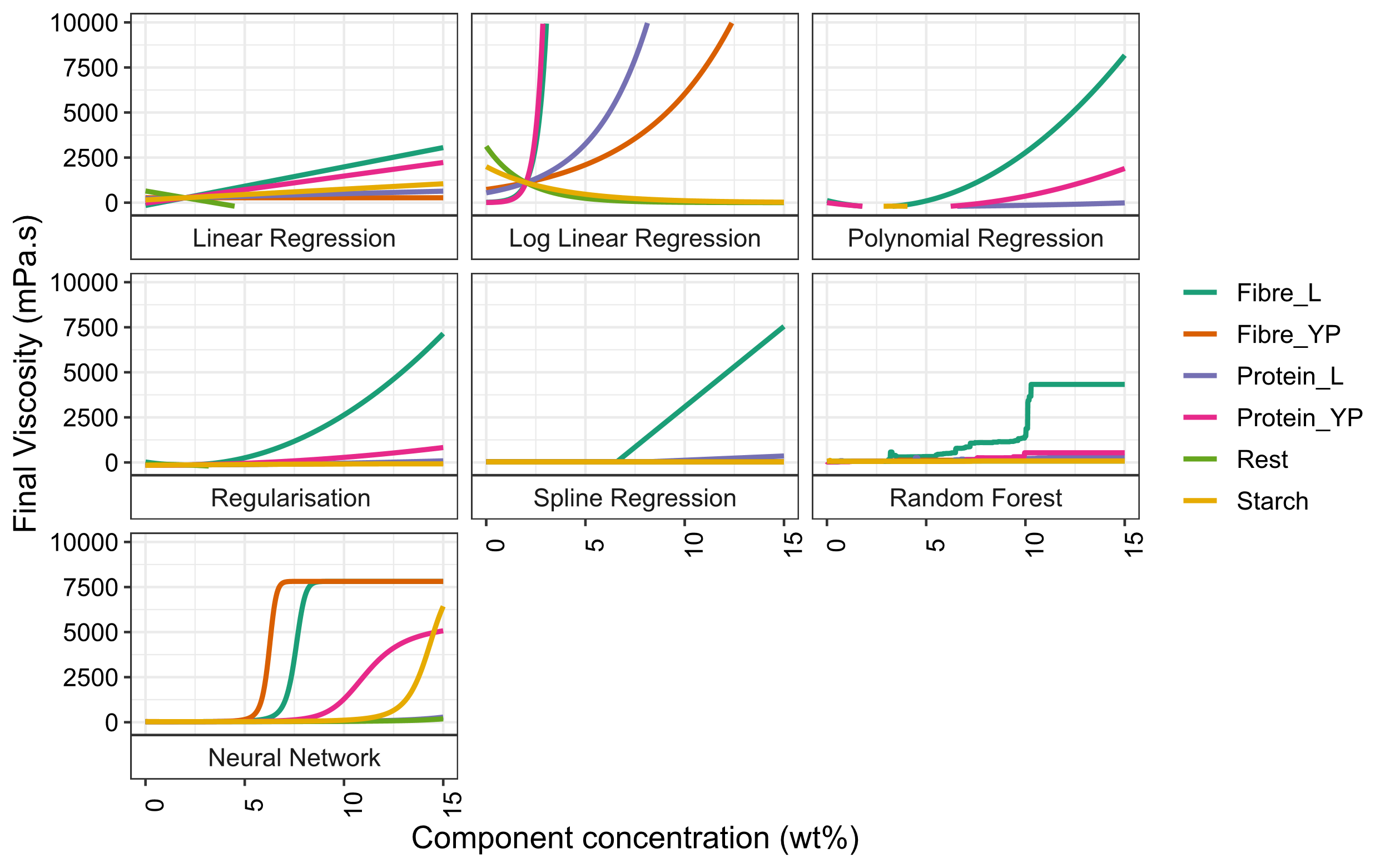


Figure 53 Scatterplot of the behaviour of each component in the evaluated models for quantifying the unheated viscosity of both yellow pea, lupine, and mixtures of those with the main macro components as independent variables. The protein and fibre is split according to crop. The composition of each component increases from 1-15 wt% while the other stay constant at 2%.


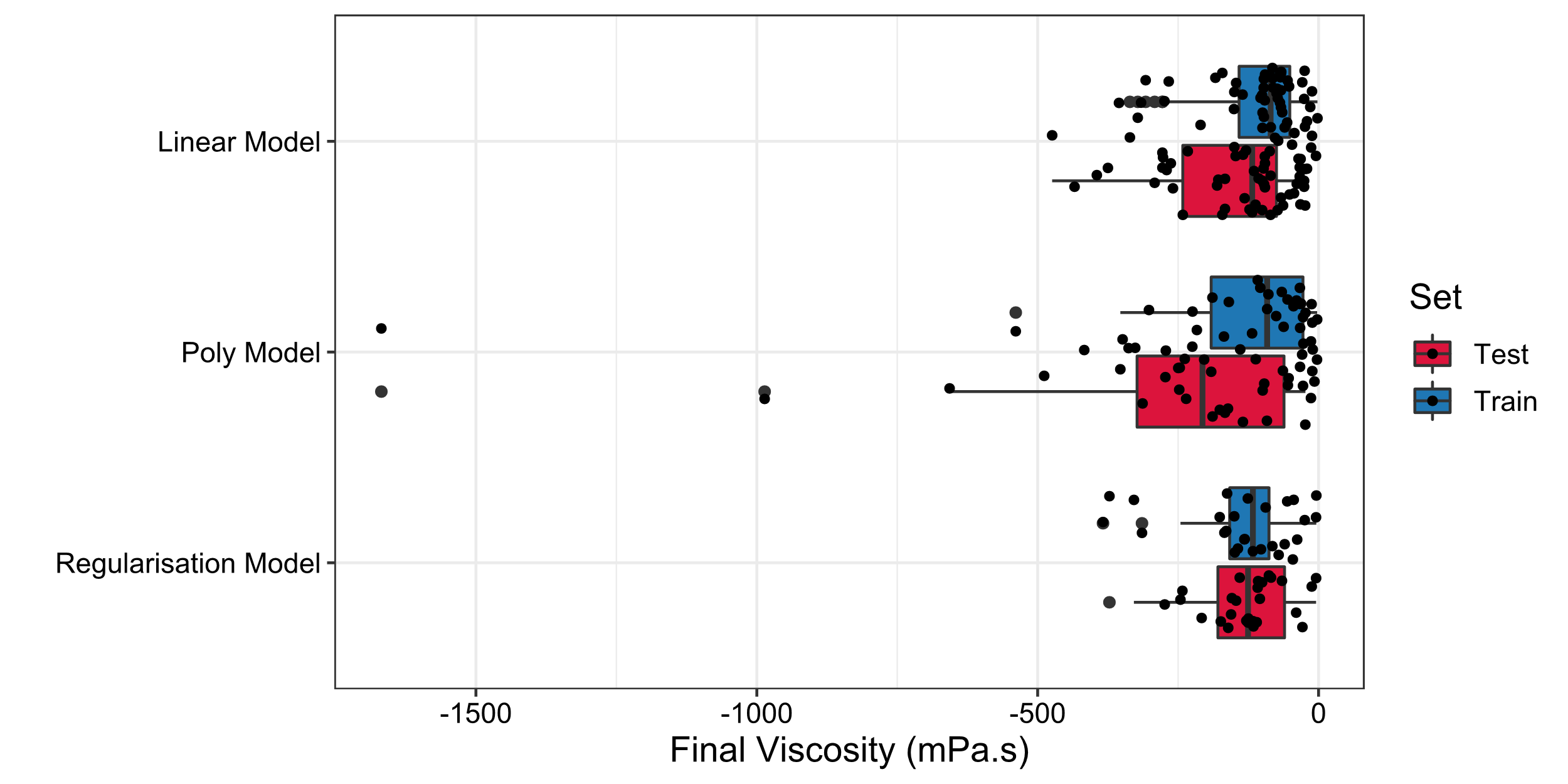


Figure 54 Boxplot of negative values predicted by the evaluated models to for quantifying the unheated viscosity both yellow pea, lupine, and mixtures of those with the main macro components as independent variables. The protein and fibre is split according to crop.
